# A Chromosome-level assembly of the Japanese eel genome, insights into gene duplication and chromosomal reorganization

**DOI:** 10.1093/gigascience/giac120

**Published:** 2022-12-08

**Authors:** Hongbo Wang, Hin Ting Wan, Bin Wu, Jianbo Jian, Alice H M Ng, Claire Yik-Lok Chung, Eugene Yui-Ching Chow, Jizhou Zhang, Anderson O L Wong, Keng Po Lai, Ting Fung Chan, Eric Lu Zhang, Chris Kong-Chu Wong

**Affiliations:** Southern Marine Science and Engineering Guangdong Laboratory (Guangzhou), China; Department of Computer Science, Hong Kong Baptist University, Hong Kong SAR; Department of Biology, Croucher Institute for Environmental Sciences, Hong Kong Baptist University, Hong Kong SAR; BGI Genomics, BGI-Shenzhen, Shenzhen 518083, China; BGI Genomics, BGI-Shenzhen, Shenzhen 518083, China; Department of Biology, Croucher Institute for Environmental Sciences, Hong Kong Baptist University, Hong Kong SAR; School of Life Sciences, State Key Laboratory of Agrobiotechnology, Hong Kong Bioinformatics Centre, the Chinese University of Hong Kong, Hong Kong SAR; School of Life Sciences, State Key Laboratory of Agrobiotechnology, Hong Kong Bioinformatics Centre, the Chinese University of Hong Kong, Hong Kong SAR; School of Life Sciences, State Key Laboratory of Agrobiotechnology, Hong Kong Bioinformatics Centre, the Chinese University of Hong Kong, Hong Kong SAR; Southern Marine Science and Engineering Guangdong Laboratory (Guangzhou), China; School of Biological Sciences, the University of Hong Kong, Hong Kong SAR; Southern Marine Science and Engineering Guangdong Laboratory (Guangzhou), China; Key Laboratory of Environmental Pollution and Integrative Omics, Guilin Medical University, Guilin, China; Southern Marine Science and Engineering Guangdong Laboratory (Guangzhou), China; School of Life Sciences, State Key Laboratory of Agrobiotechnology, Hong Kong Bioinformatics Centre, the Chinese University of Hong Kong, Hong Kong SAR; Department of Computer Science, Hong Kong Baptist University, Hong Kong SAR; Southern Marine Science and Engineering Guangdong Laboratory (Guangzhou), China; Department of Biology, Croucher Institute for Environmental Sciences, Hong Kong Baptist University, Hong Kong SAR

**Keywords:** *Anguilla japonica*, Phylogenomics, Gene Expansion, olfactory receptors, Ca^2+^channels

## Abstract

Japanese eels (*Anguilla japonica*) are commercially important species, harvested extensively for food. Currently, this and related species (American and European eels) are challenging to breed on a commercial basis. As a result, the wild stock is used for aquaculture. Moreover, climate change, habitat loss, water pollution, and altered ocean currents affect eel populations negatively. Accordingly, the International Union for Conservation of Nature lists Japanese eels as endangered and on its red list. Here we presented a high-quality genome assembly for Japanese eels and demonstrated that large chromosome reorganizations occurred in the events of third-round whole-genome duplications (3R-WRDs). Several chromosomal fusions and fissions have reduced the ancestral protochromosomal number of 25 to 19 in the Anguilla lineage. A phylogenetic analysis of the expanded gene families showed that the olfactory receptors (group δ and ζ genes) and voltage-gated Ca^2+^ channels expanded significantly. Both gene families are crucial for olfaction and neurophysiology. Additional tandem and proximal duplications occurred following 3R-WGD to acquire immune-related genes for an adaptive advantage against various pathogens. The Japanese eel assembly presented here can be used to study other Anguilla species relating to evolution and conservation.

## Introduction

Fishes are highly diverse species living in many ecological habitats, including freshwater, estuarine, and the ocean [1]. Over 99% of fish species are known to be stenohaline, inhabiting freshwater or marine environments. In contrast, euryhaline fishes are diadromous, migrating between freshwater and saltwater environments in their life cycles [2]. Catadromous fishes like eels spawn in the sea and migrate to inland freshwater to grow and mature. Eels are ecologically and economically essential, serving as indicators of the healthiness of coastal environments and resources in aquaculture. The fish are not bred in captivity [3]. In current practices, glass eels (juvenile life stage) are captured from the wild and raised on farms. Over 90% of freshwater eels consumed worldwide are farm-raised. Since the 1960s, catches of Anguillid eels, like European and Japanese eels, have declined by over 50% to 80%. In a 2014 report from the International Union for Conservation of Nature, the American, European, and Japanese eels have been listed as at high risk of extinction. The decline in eel populations is abetted by soaring demand from global markets. In addition, overfishing, habitat loss, dams [4], water pollution [5], parasites [6], eel larvae predation by mesopelagic fishes [7], climate change, and altered ocean currents [8] are known to cause population decline.

From the evolutionary perspective, eels are among the extant basal groups of teleost ray-finned fishes after the 3-round whole-genome duplication (3R-WGD) [9]. The ray-finned fishes, including holostei (bowfin, gar), chondrostei (sturgeon, paddlefish, starlet), and cladistia (bichir, ropefish), diverged from lobe-finned fishes (coelacanth, lungfish) about 450 million years ago (Mya) [10]. Comparing eels with other ray-finned fishes would shed light on fish evolution. In 2012, the first draft genome sequences of the Japanese eels (genome size 1.15 Gb, N50 of 52.8 Kbp, number of scaffolds 323,776) and European eels (0.923 Gb, N50 of 78 Kbp) were published [11, 12]. Afterward, double-digested restriction site–associated DNA sequencing was applied to construct a linkage map of the Japanese eel, generating 19 linkage groups for subsequent quantitative trait loci analysis [13]. The Japanese eel's draft genome's annotation was further enhanced using transcriptome data [14] and the phylogenetic analysis of rhodopsin genes in the Japanese eel (1.15 Gb, N50 of 472 Kbp, number of scaffolds 195,366) [15]. Moreover, the genome assembly of the European eel was improved to 0.979 Gb, N50 of 57.2 Mbp, number of scaffolds 54 [16] and to 1.03 Gb, N50 of 55.98 Mbp, number of scaffolds 1,466 [17]. A draft genome of the American eel (with a total size of 1.41 Gb, N50 of 86.6 Kbp, number of scaffolds 79,209) was published in 2017, and 26,564 genes were annotated [18]. In 2019, the assembly of a Japanese genome of 1.18 Gb [19] was improved with 256,649 contigs, 41,687 scaffolds, and a scaffold N50 of 1.03 Mbp. Currently, only the draft genome is available for Japanese eels. This study aimed to provide high-quality genome assemblies and understand karyotype evolution in early ray-finned fishes. The genome-scale data can provide ecological and conservation information by identifying adaptive and disease-resistant alleles.

## Materials and Methods

### Genome sequencing

A market-purchased female Japanese eel, *Anguilla japonica* (NCBI:txid7937; Fishbase ID: 295), was kept in a freshwater tank for a week with aeration. Blood sample was taken from the fish, snapped frozen in liquid nitrogen, and then stored at −80°C. Genomic DNA was extracted from the blood sample. DNA sequencing data were generated by different platforms, including Oxford Nanopore (ONT) long reads, PacBio continuous long reads (CLRs), Illumina short reads, Illumina mate-pair reads, 10× Chromium linked reads, DNase Hi-C (Omni-C), and Bionano optical mapping (BioNano Irys system, RRID:SCR_016754).

The library for ONT long-read sequencing was prepared using the Ligation Sequencing Kit (LSK109) and sequenced using the Nanopore PromethION P48 (Oxford Nanopore Technologies, UK) sequencer with the flow cells (R9.4.1) and the basecaller version Guppy (Guppy Project, RRID:SCR_006255) 3.2.10. For PacBio CLR sequencing, the SMRTbell templates were prepared using Sequel Binding Kit 1.0 and sequenced on the PacBio Sequel System (PacBio, USA). For Illumina short reads and mate-pair sequencing, the libraries were prepared using the TruSeq DNA PCRFree Kit (Illumina, USA) and Nextera Mate Pair Library Preparation Kit (Illumina, USA) (gel plus), respectively. They were sequenced with 2× 150-bp reads on an Illumina HiSeq X Ten (Illumina HiSeq X Ten, RRID:SCR_016385) instrument. The library for linked reads was prepared by a 10× Genomics Chromium (10xGenomics, USA) system with the Chromium Genome library (v2) and sequenced with 2× 150-bp reads on an Illumina NovaSeq 6000 (Illumina NovaSeq 6000 Sequencing System, RRID:SCR_020150) instrument. Dovetail Omni-C Kit (Dovetail, USA) was used for Hi-C library preparation, which used NEBNext Ultra (Illumina, USA) enzyme and Illumina-compatible adapters. Biotin-containing fragments were isolated using streptavidin beads before PCR enrichment. The library was sequenced with 2× 150-bp reads on an Illumina HiSeqX platform. The Bionano optical mapping was generated by 3 enzymes, 2 from Irys (Nt.BspQI and Nb.BssSI) and 1 from Saphyr (RRID:SCR_017992) (DLE1). We stretched and captured the images of fluorescently labeled DNA molecules in Irys and Saphyr G1.2 chips. The labeling distances were extracted from the images and recorded into the raw molecule files. Molecules over 150 Kbp were assembled into consensus maps using Bionano Solve for further analysis (Supplementary Table S1).

### Genome assembly on ONT long reads

MitoZ software (v2.4) [20] was used to assemble and annotate the mitochondrial genome of the Japanese eel. We assembled ONT long reads using Canu (RRID:SCR_015880) v2 [21], Wtdbg2 (WTDBG, RRID:SCR_017225) v2.5 [22], and Flye (RRID:SCR_017016) v2.71 [23] separately and merged their contigs using Quickmerge [24] to achieve a balance between contig N50 and percentage of complete genes in vertebrate species. We used Racon (RRID:SCR_017642) v1.4.16 [25] for 2 rounds and Medaka v1.6.1 for 1 round to self-correct assembly errors using ONT reads, respectively. The PacBio CLR was then incorporated for error correction using Racon for 2 rounds. As the last step, we further improved the assembly by integrating Illumina short reads and mate-pair libraries using Pilon (RRID:SCR_014731) v1.23 [26] for 2 rounds.

### Scaffolding on 10× linked-reads, Bionano, and Hi-C

We applied Tigmint v1.1.2 [27] and ARKS v1.0.3 [28] to correct misassembled contigs and linking contigs into scaffolds according to the shared barcodes from 10× linked reads. We used OMGS [29] to integrate 3 enzymes used in Bionano optical mapping for scaffolding. We further extended the scaffolds using 3-dimensional DNA (180,419) [30] based on the Hi-C data from the Dovetail Omni-C library and refined the scaffolds manually by JuiceBox (RRID:SCR_021172) v1.11.08 [31] to extend the scaffolds to the corresponding chromosome scale.

### Tandem repeats and transposable elements annotation

Tandem Repeats Finder v4.09 [32] was applied to annotate tandem repetitive sequences. We utilized homolog-based and *de novo* approaches to annotate transposable elements (TEs) in the Japanese eel genome. For the homolog-based approach, RepeatMasker v4.0.7 [33] and RepeatProteinMask v4.0.7 were used to identify the repeats by aligning the known TE sequences from RepBase (RRID:SCR_021169) v21.12 database [34] to the genome. LTR_FINDER (RRID:SCR_015247) v1.06 [35] was used to infer long terminal repeat retrotransposons. For the *de novo* approach, RepeatModeler (RRID:SCR_015027) v1.0.8 was used to detect the TE families and repeat boundaries by integrating 3 complementary *de novo* repeat finding programs. RepeatMasker collected the union of these tools' results and annotated the genome accordingly.

### Genes and their functional annotation

Three types of methods were used to annotate the protein-coding genes in the genome, including *de novo*, homology-based, and transcriptome-based annotations. Maker (RRID:SCR_005309) v2.31.8 [36] was adopted for homology annotation using the protein sequences from the 5 closely related species, including European eel (*Anguilla anguilla*), zebrafish (*Danio rerio*), Indo-Pacific tarpons (*Megalops cyprinoides*), Asian arowana (*Scleropages formosus*), and spotted gar (*Lepisosteus oculatus*), based on the phylogeny of teleost fishes [37].

*De novo* annotation was performed using Augustus (RRID:SCR_008417) v3.2.1 [38] and SNAP (SNP Annotation and Proxy Search, RRID:SCR_002127) v1 [39] by training a model using 3,000 complete genes obtained from homology prediction. Transcriptome annotation was performed by aligning RNA sequencing data (Bioproject: PRJNA578238) to the genome with HISAT2 (RRID:SCR_015530) v2.1.0 [40] and assembling transcript sequences with Trinity (RRID:SCR_013048) v2.10.0 [41]. Pasa_lite was used to correct assembly errors to obtain the final transcripts. Maker v2.31.8 was further applied to integrate the 3 annotations, followed by the second round of homology annotation to refine the final gene set.

Gene functional annotation was performed by aligning the predicted gene sequences to protein sequences using BLAST v2.2.31 [42] in the six databases, including NCBI Non-Redundant Protein Sequence (NR), KEGG [43], SwissProt [44], KOG [45], Gene Ontology [46], and TrEMBL (Uniprot version 2020–06). We further searched the secondary structure domain database for gene function prediction using InterProscan [47].

### Evaluation of genome assembly and gene annotation

BUSCO (RRID:SCR_015008) v5.1.2 [48] was used to evaluate genome assembly and gene annotation by calculating the completeness of single-copy orthologs. We selected the ray-finned fish single-copy ortholog direct homologous gene database actinopterygii_odb10 (which contains 3,640 core single-copy direct homologous gene proteins), the closest relative to the Japanese eel in the OrthoDB database (RRID:SCR_011980), to compare.

### Annotation of conserved noncoding elements

tRNAscan-SE (RRID:SCR_010835) 1.3.1 [49] was used to identify transfer RNA (tRNA) sequences in the genome families. We annotated the ribosomal RNA (rRNA) sequences by aligning the conserved rRNA sequences from the 5 closely related fish species (European eel, zebrafish, tarpons, arowana, and spotted gar) to the genome using BLASTN (RRID:SCR_001598) [50]. The microRNAs and small nuclear RNAs were annotated by aligning the corresponding sequences from Rfam (RRID:SCR_007891) v12 [51] to the genome.

### Phylogenetic analysis, gene expansion, and gene contraction

OrthoMCL (v2.0) [52] was used to identify gene families by grouping orthologous proteins. We applied the maximum likelihood method [53] and RAxML (RRID:SCR_006086) v2.2.3 [54] to reconstruct the phylogenetic tree using 4-fold degenerate sites (4dTv) in single-copy orthologs from the 12 fish species, including *Anguilla rostrata* (American eel, GenBank assembly: GCA_001606085.1), *A. anguilla* (European eel, GCA_013347855.1), *A. japonica* (Japanese eel), *M. cyprinoides* (tarpons, GCA_013368585.1), *S. formosus* (arowana, GCA_900964775.1), *Gadus morhua* (Atlantic cod, GCA_902167405.1), *Oryzias latipes* (medaka, GCA_002234675.1), *D. rerio* (zebrafish, GCA_000002035.4),*L. oculatus* (spotted gar, GCA_000242695.1), *Erpetoichthys calabaricus* (reed fish, GCA_900747795.2), *Latimeria chalumnae* (coelacanth, GCA_000225785.1), and *Callorhinchus milii* (Australian ghost shark, GCA_000165045.2). We estimated the divergence times for single-copy orthologos using mcmctree in PAML (RRID:SCR_014932) package v4.8a [55] based on the predefined times from the TimeTree (RRID:SCR_021162) website: *D. rerio* with *O. latipes* (180.0–264.0 Mya), *M. cyprinoides* with *A. anguilla* (162.2–197.3 Mya), *C. milii* with *D. rerio* (442.7–515.5 Mya), and *E. calabaricus* with *D. rerio* (381.0–407.0 Mya). To estimate gene family expansion and contraction, we used CAFÉ (RRID:SCR_005983) v4.2.1 [56] to model gene expansions and contractions, as well as the divergence times.

### Identification of olfactory receptor genes

We identified olfactory receptor (OR) genes using the pipeline described in GitHub [57], while candidate genes were filtered via the NR database. The OR gene identified in a previous study [58] was used as a query sequence. TBLASTN (RRID:SCR_011822) v2.2.26 [59] was used to identify genomic regions containing OR genes in the 10 fish species (European eel, Japanese eel, tarpons, arowana, medaka, Atlantic cod, zebrafish, spotted gar, coelacanth, and Australian ghost shark). Only the nonoverlapping BLAST hit regions were extracted. The 1-kb upstream and downstream flanking regions were used as the input to EMBOSS (RRID:SCR_008493) v6.6.0 [60]. Using EMBOSS, we generated open reading frames (ORFs), translated the ORFs into protein sequences, and then ran BLASTP (RRID:SCR_001010) v2.2.26 to remove sequences that did not match genes already known in SwissProt and NR. InterProscan was used to determine the secondary structures of the predicted OR genes. Some genes were filtered due to lacking the 7 transmembrane domains. The maximum likelihood phylogenetic tree was reconstructed using IQ-TREE (RRID:SCR_017254) v2.2.0.3 [61] based on the multiple sequencing alignments on the OR gene sequences with MAFFT (RRID:SCR_011811) v7.505 [62].

### Genome evolution analysis

MCscanX v1.5.1 [63] and macrosynteny visualization (jcvi) were used to screen for collinear blocks with at least 30 genes [64] in *A. japonica, A. anguilla, A. rostrata, M. cyprinoides*,and *Lepisosteus oculatus*. The numbers of nonsynonymous substitutions (Ka) and synonymous substitutions (Ks) were calculated using KaKs_calculator2.0 [65]. In addition, we calculated 4dTv values to estimate the WGD events in the Japanese eel genome. We identified gene duplicates in the genomes of Japanese eel, zebrafish, arowana, medaka, and Atlantic cod using the DupGen_finder pipeline [66], using spotted gars as an out-group. It classified gene duplication patterns into 5 categories: whole-genome duplications, tandem duplications, proximal duplications (nontandem duplications that are separated by 10 genes on the same chromosome), transposable duplications, and scattered duplications (duplications other than the 4 categories mentioned above).

### Ancestral chromosome reconfiguration

Ancestral eel/tarpon karyotype (AETK) was constructed using *A. japonica* (Japanese eel), *M. cyprinoides* (tarpon), and *S. formosus* (arowana, out-group). The ancestral teleosts karyotype (ATK) was constructed using zebrafish, *S. formosus* (arowana), and *L. oculatus* (spotted gar, out-group) [67]. This was implemented using BLASTP [68] to obtain homologous gene pairs between species. The default parameters of MCScanX were then applied to obtain the collinear blocks of chromosomes between species. Finally, the karyotype of the ancestor was constructed using ANGeS v1.01 [69].

## Results

### Genome assembly and annotation

**Figure 1:**
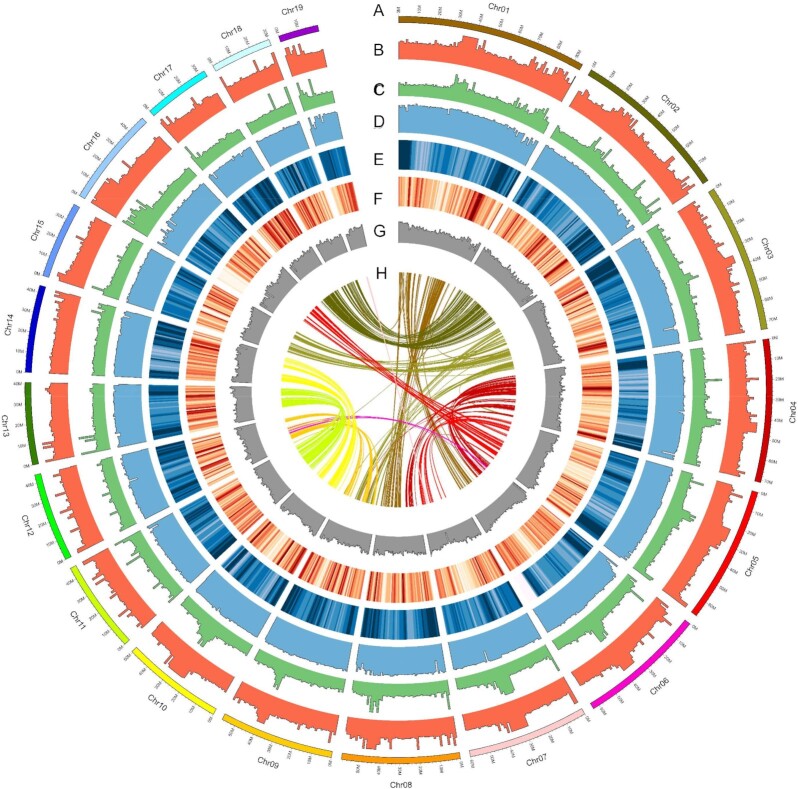
The genome landscape of the Japanese eel, *Anguilla japonica*. From outer to inner circle: (A) length of 19 chromosomes (Mb); (B) read depth of ONT long reads; (C) read depth of PacBio CLR long reads; (D) read depth of Illumina short reads; (E) distribution of transposon sequences; (F) distribution of protein-coding gene; (G) GC content; (H) Collinear blocks of at least 10 genes in the genome. The window size is 1 MB.

In this study, MitoZ software was used to assemble and annotate the mitochondrial genome (16.686 Kb) of our sample to confirm the species' identity (Materials and Methods). The data matched with the Japanese eel mitochondrial genome (GenBank ID AB038556.2) of the NR database from NCBI (Supplementary Figs. S1 and S2). We hierarchically integrated the sequencing data from different platforms to characterize their strength in *de novo* assembly and annotation (Supplementary Fig. S3). The draft genome was generated using ONT contigs followed by error correction and scaffolding based on the genomic spans of different sequencing technologies [70] (Materials and Methods). A high-quality Japanese female eel's reference genome was then obtained through the integration of ONT long reads (234×, 239.64 Gb), PacBio CLR (261×, 267 Gb), 10× Chromium linked reads (313×, 319.7 Gb), Hi-C data (48×, 48.99 Gb), Illumina short reads (148×, 151.89 Gb), and mate-pair reads (127×, 130.5 Gb). The contigs from ONT long reads resulted in a significantly improved N50 (25.82 Mb) without losing many complete genes (54.6%) (Supplementary Table S2). With reduced assembly errors, the percentage of complete genes increased from 54.6% to 90.1%, indicating a higher base quality (Supplementary Table S3). For scaffolding, 10× linked reads, Bionano, and Hi-C data were used sequentially according to fragment length to increase assembly continuity and assign scaffolds to 19 chromosomes (Fig. 1, Supplementary Fig. S3, and Supplementary Table S4). As a result, the genome size is 1.028 Gb, the contig N50 is 21.48 Mb, and the scaffold N50 is 58.7 Mb. The chromosome lengths range from 19.93 to 94.28 Mb. According to *actinopterygii_odb10* in the BUSCO database, 94% of the single-copy direct homologs in the ray-finned fishes were assembled in Japanese eels (Supplementary Table S5). The repeat elements accounted for 30.49% of the whole genome (Supplementary Table S6). The TEs were excluded from gene annotation (Supplementary Table S7). Japanese eels have a higher percentage (30.49%) of repetitive sequences, which may explain their larger genome, compared to European eels (*A. anguilla* 0.979 Gb) [16]. Even so, the Japanese and European eels have a 1:1 correspondence pattern of chromosomes and 19,325 homologous genes, demonstrating their matching structure (Supplementary Fig. S4).

By combining gene annotations from homology, *de novo*, and transcriptome annotations (Materials and Methods), we identified 29,982 coding genes (Table 1). We functionally annotated 97.44% (29,219) of these genes (Supplementary Table S8) using the publicly available databases (Materials and Methods). Additionally, 21,606 genes were annotated by all 5 major protein databases (Supplementary Fig. S5), with signal transduction pathways most abundant in KEGG (Supplementary Fig. S6) and KOG (Supplementary Fig. S7). BUSCO analysis showed that 94.7% of the single-copy orthologs could be found in the ray-finned fish single-copy direct homology gene database actinopterygii_odb10 (Supplementary Table S9). The protein-coding genes in Japanese eels have an average length of 10.2 Kbp and contain approximately 9 exons (Table 1), which have an average length of 1.6 Kbp (Supplementary Table S10). The gene structure of Japanese eels is similar to those of 4 closely related species (Supplementary Fig. S8). The genome assembly has a greater number of predicted genes (29,982 genes) than the Atlantic species, European (25,903 genes), and American (26,565 genes) eels. Additionally, 17,095 noncoding RNAs were predicted, including 1,042 tRNAs, 1,771 rRNAs, and 3,974 microRNAs in Japanese eels.

**Table 1:** Statistics of *Anguilla japonica* genome assembly and annotation

Assembly feature	*Anguilla japonica*
Genome size, Gb	1.028
No. of contigs	811
Contig N50, Mbp	21.48
Contig N90, Kbp	716.98
Longest contig, Mbp	57.08
No. of scaffolds	86
Scaffold N50, Mbp	58.71
Scaffold N90, Mbp	38.29
Longest scaffold, Mbp	94.29
Repeat portion of assembly, %	30.48
No. of genes	29,982
GC%	44
Genes average length, bp	10,265.73
Average exons per gene	9

### Phylogenomics and demographic history

**Figure 2:**
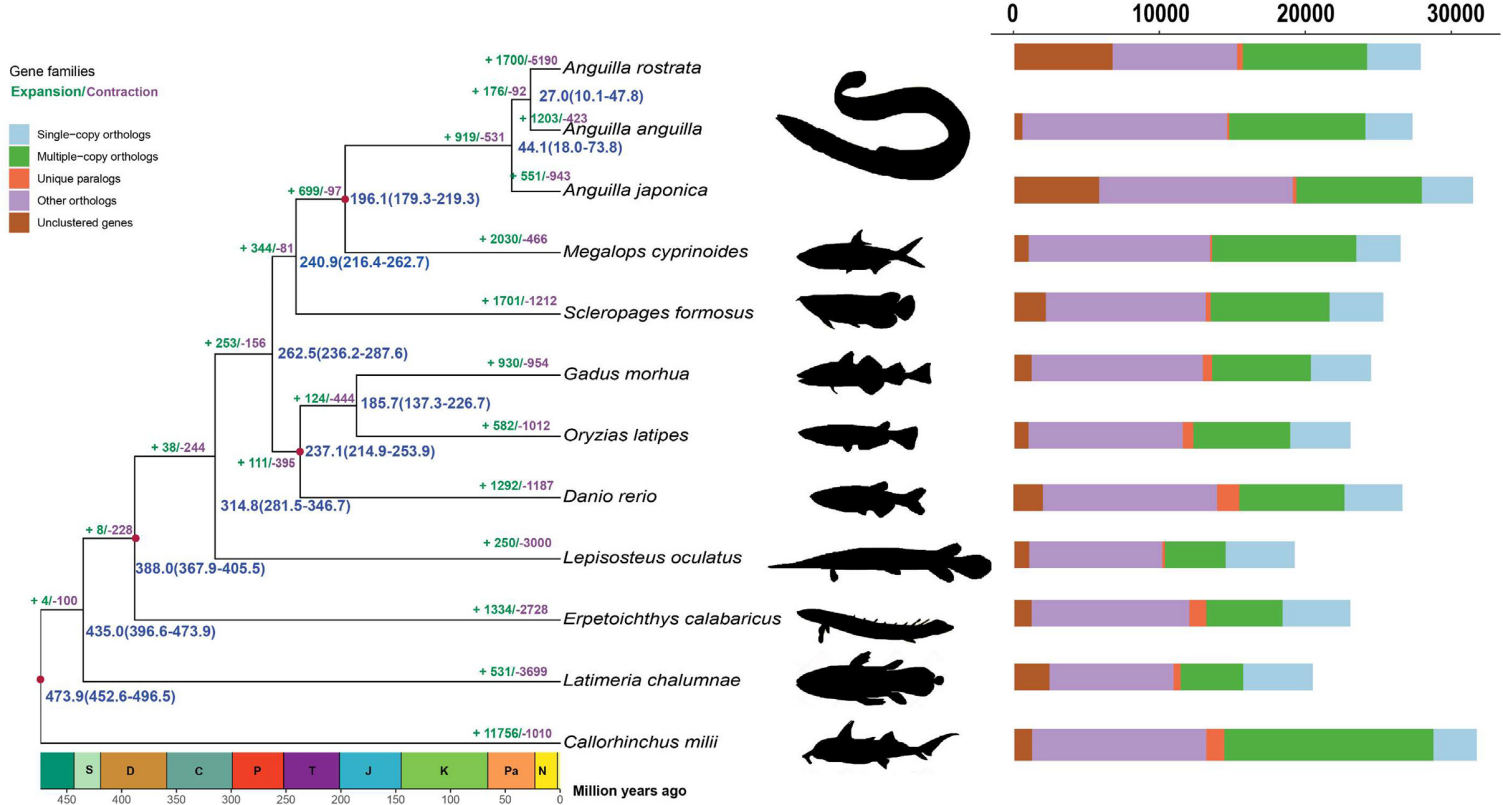
Phylogenetic relationship, divergence times, and gene families of *Anguilla* species and relevant bony and cartilaginous fishes. The gene families' expansions (numbers in green) and contractions (numbers in purple) are shown at individual lineages. Each node shows the estimated divergence times (blue numbers, Mya) and the 95% confidence intervals for these dates. Red dots indicate times taken from the TimeTree website. The orange star shows the 3R-WGD event. Geological periods from left to right: S = Silurian, D = Devonian, C = Carboniferous, P = Permian, T = Triassic, J = Jurassic, K = Cretaceous, Pa = Paleogene, N = Neogene. A comparison of gene families associated with orthologs and paralogs in Japanese eel and the 11 fish species.

The orthology analysis of 12 species' coding genes identified 21,653 gene family clusters. *A. japonica*’s genome contains 29,982 coding genes, including 3,347 single-copy orthologs, 8,204 multiple-copy orthologs, 233 unique paralogs, 12,662 other orthologs, and 5,536 unclustered genes. A phylogenetic tree was reconstructed by identifying the 4-fold synonymous third-codon transversion (4dTv) loci in the 1,131 single-copy orthologs from the 12 fish species (Fig. 2). American and European eels diverged from their ancestors about 27.0 Mya from a common ancestor. With a divergence time of approximately 44.1 Mya, the Japanese eel was distant from the Atlantic eel species. Compared with the 3 freshwater eels (Anguilliformes) and tarpons (Elopiformes), the members of the order Elopomorpha, their common ancestor, diverged 196.1 Mya. Elopomorpha and Osteoglossomorpha (i.e., arowana) are the closest evolutionary relatives at the basal branch of teleosts [17], separating 240.9 Mya. *Gadiformes* (e.g., Atlantic cod) and *Cypriniformes* (e.g., medaka, zebrafish) diverged from the Eloposteoglossocephala clade at 262.5 Mya. Above are fish groups that had undergone 3R-WGD. Compared to the out-groups, spotted gars, reed fish, coelacanths, and Australian ghost sharks underwent only 2 rounds of whole-genome duplication (2R-WGD).

### Expanded gene families and gene duplication

**Figure 3:**
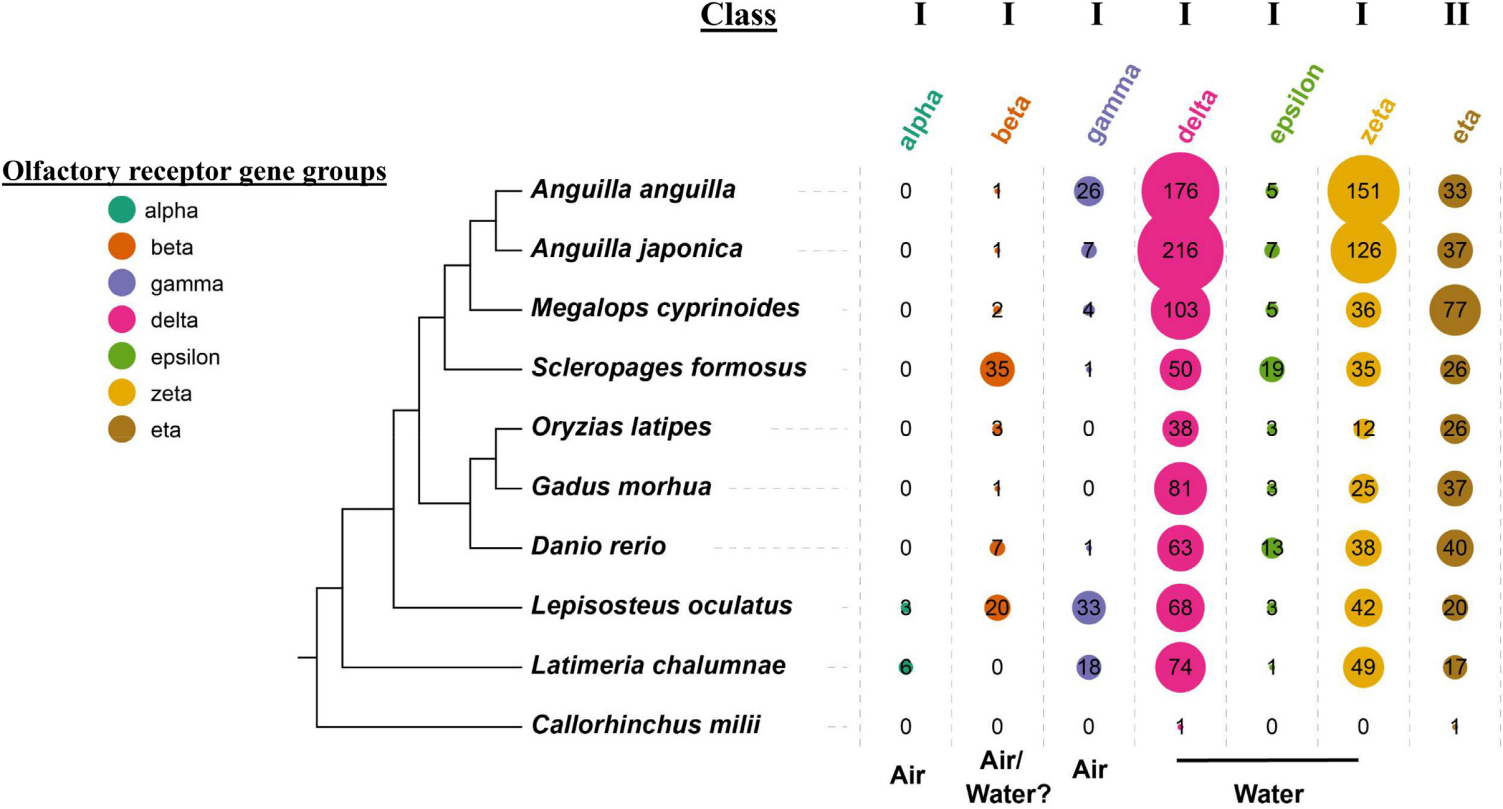
Number and classification of OR genes for 10 fish species. On the left is the phylogenetic tree of the 10 species. The number of OR genes is shown on the right. The size of the circle indicates the number of OR genes.

The expansion and contraction of gene families reflect the evolution of organisms' adaptations to their environments. Ortholog analysis of genes from the 12 species (Materials and Methods) identified 21,652 gene family clusters. By removing gene families with too many (≥200) or too few (≤2) genes, we achieved 129,862 genes to evaluate the expansion and contraction of gene families (Fig. 2). Compared to the 9 other species (Materials and Methods), the 3 freshwater eels had expanded 771 and contracted 467 gene families, resulting in an increase of 919 and loss of 531 genes, respectively (Supplementary Table S11). Among those, the 3 freshwater eel species exhibited a significant expansion in the OR gene family, which is crucial for detecting odor molecules under varying environmental conditions. A retrospective analysis of the OR receptors across 10 species' genomes was performed, and 7 types of OR receptors were identified—alpha (α), beta (β), gamma (γ), delta (δ), epsilon (ε), zeta (ζ), and eta (η)—based on a previous study [58]. Compared to other fish species, the Japanese eels had a significantly higher number of OR genes (394) (Fig. 3), located on the 4 chromosomes—Chr4 (2 genes), Chr9 (153 genes), Chr11 (1 gene), and Chr12 (238 genes). Similarly, the European eel contains 392 OR genes. The δ and ζ genes are the major OR genes in the eels.

Comparing the Japanese eel to the other 11 species, 433 gene families increased, with a total increase of 551 genes. On the other hand, a total of 943 genes were lost from 782 gene families (Supplementary Table S12). It is interesting to note that Ca^2+^ and K^+^ channel families were identified. Calcium and potassium play significant roles in neuronal excitability, muscle contraction, fertilization, and energy metabolism. Interestingly, the other expanded gene families include (i) the assembly of thick myosin filament in skeletal muscle, (ii) lipoprotein receptor–related protein (metabolic and morphogenetic pathways), and (iii) isocitrate and isopropyl malate dehydrogenases family (carbohydrate and amino acid metabolism).

**Figure 4:**
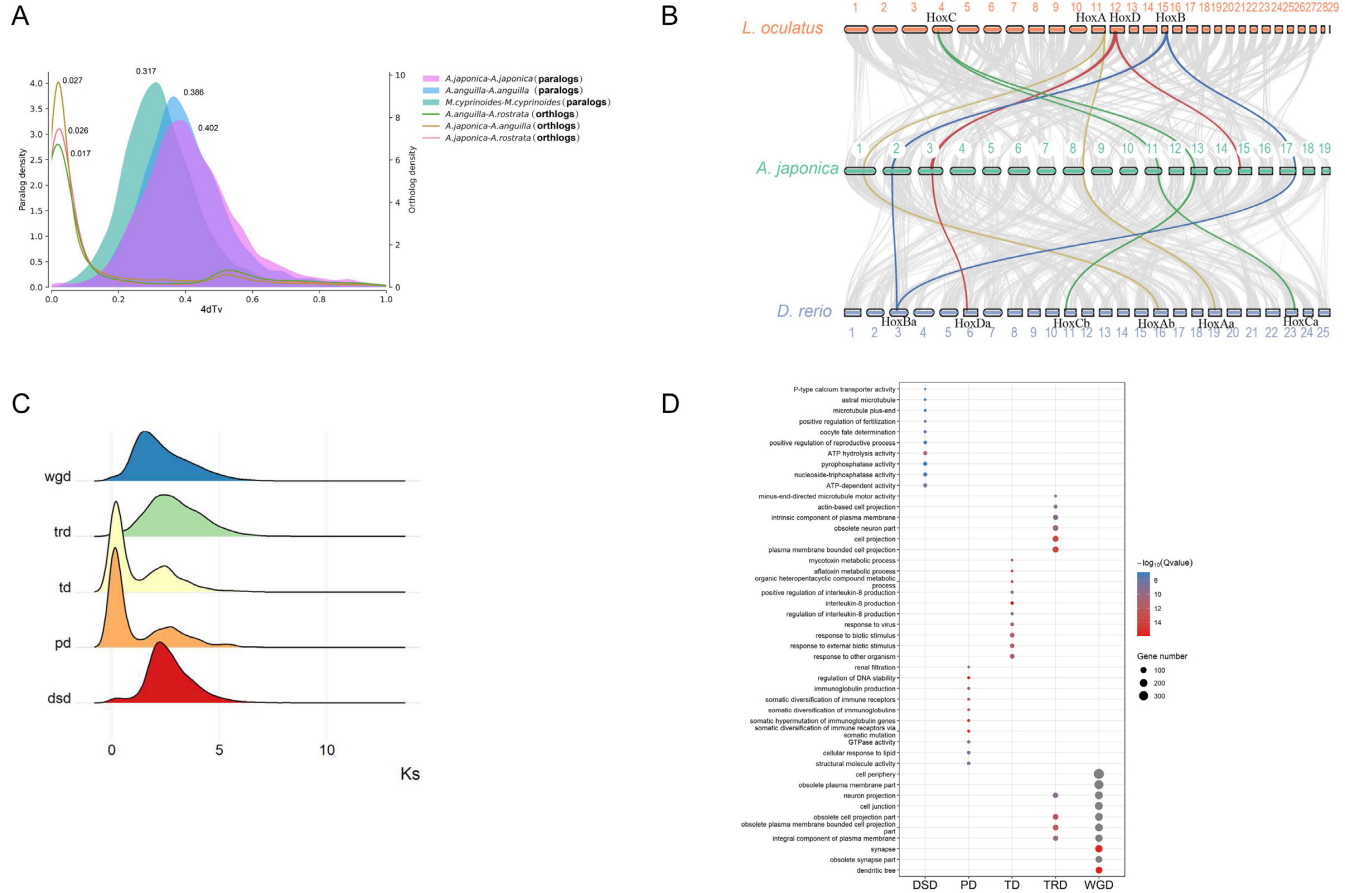
(A) Fourfold synonymous third-codon transversion rate (4dTv) distributions of homologous gene pairs for intraspecies (paralog density) and interspecies (ortholog density) comparisons. (B) The collinear relationships of syntenic blocks among *A. japonica, D. rerio*, and *L. oculatus*. The numbers indicate the corresponding chromosomes for each species. In *L. oculatus*, the 29th chromosome is 293.7 Kb long, which has no collinearity with that of *A. japonica*. Based on homologous blocks of at least 10 genes, gene links between these 2 species were identified. The 4 collinear blocks that contain Hox genes are shown in green, yellow, red, and blue. (C) Ks distributions of syntenic gene pairs from different gene duplications (wgd, whole genome duplication; trd, transposable duplication; td, tandem duplication; pd, proximal duplication; dsd, dispersed duplication). The y-axis shows the distribution of Ks values. (D) Enrichment analysis of 5 duplicated expansion gene families, with the circles' color representing the GO's statistical significance. The circle size represents the number of genes.

It was reported that freshwater eels (European and Japanese) had many paralogous pairs after splitting from the Osteoglossomorpha lineage [71]. The observation suggested 4 rounds of whole-genome duplication (4R-WGD) or lineage-specific re-diploidization in some duplicated genomic regions. We studied the distribution of 4dTv and Ks values of genome-wide direct homologous gene pairs in Japanese eels, European eels, and tarpons. There were 4dTv values of 0.402, 0.386, and 0.317 for *A. japonica, A. Anguilla, and M. cyprinoides*, respectively (Fig. 4A and Supplementary Fig. S9). Additional WGD events were not detected. We also compared the syntenic blocks at Hox A–D loci with those in spotted gar (2R-WGD) and zebrafish (3R-WGD) (Fig. 4B). By identifying ohnolog pairs using collinear blocks of 10 genes, we discovered that the Japanese eel's genome has 8 clusters of Hox loci on chromosomes 1, 2, 3, 8, 11, 13, 15, and 17. In contrast, spotted gar has 4 clusters on chromosomes 4, 11, 12, and 13. Zebrafish underwent 3R-WGD with 7 Hox gene clusters (lacking HoxDb) [72]. We found that 6 (HoxAa, HoxAb, HoxBa, HoxCa, HoxCb, and HoxDa) out of 7 Hox clusters of zebrafish exhibit ohnolog pairs with eels. Because zebrafish HoxBb gene clusters contain only 4 genes, eel HoxBb and zebrafish HoxBb did not show the ohnolog pair. Collectively, the data do not support the presence of 4R-WGD in Japanese eels.

There are 21,249 duplicated genes identified among the 29,982 coding genes in the Japanese eel genome. Based on their duplication patterns, DupGen_finder (Materials and Methods) classified the duplicated genes into 5 categories: (i) 9,890 WGDs (46.54%), (ii) 1,420 tandem duplicates (TDs, 6.68%), (iii) 768 proximal duplicates (PDs, 3.61%), (iv) 3,975 transposed duplicates (TRDs, 18.71%), and (v) 5,196 dispersed duplicates (DSD, 24.45%). We then calculated the Ks and Ka/Ks values for these 5 gene categories. Ks distribution indicates that TD and PD revealed additional duplication after 3R-WGD (Fig. 4C). In addition, both TD and PD duplicates exhibited high Ka/Ks ratios, indicating high selection pressure, which was probably related to environmental adaptation. TD and PD duplicated genes are mainly involved in immune responses (e.g., the production of interleukin 8, virus and biotic stress, somatic hypermutation of immunoglobulin genes, diversification and production of immunoglobulins and immunoreceptors) (Fig. 4D). Nonetheless, WGD was associated with 32.98% of the total number of coding genes (29,982) in Japanese eels. Gene duplications in other fish species were also analyzed using the DupGen_finder pipeline [66] and compared. Japanese eels were found to share the same level of WGD duplication of coding genes as arowana (37.60%), as both are extant members of the basal teleost group. However, it differs from the majority of teleosts, such as medaka (6.09%), zebrafish (9.51%), and Atlantic cod (4.68%). In Japanese eel, these duplicated gene functions were associated with neuronal (dendrites, synapses, neuron projections, obsolete synapses) and cell–cell junctions (cellular periphery, cell junctions, integral components of plasma membranes, obsolete plasma membranes, and cell projections). TRD shows a similar profile of changes. In DSD, duplication genes function in microtubules, reproduction (oocyte fate determination, fertilization), and ATP metabolism.

### Evolution of chromosome number in Japanese eels

**Figure 5:**
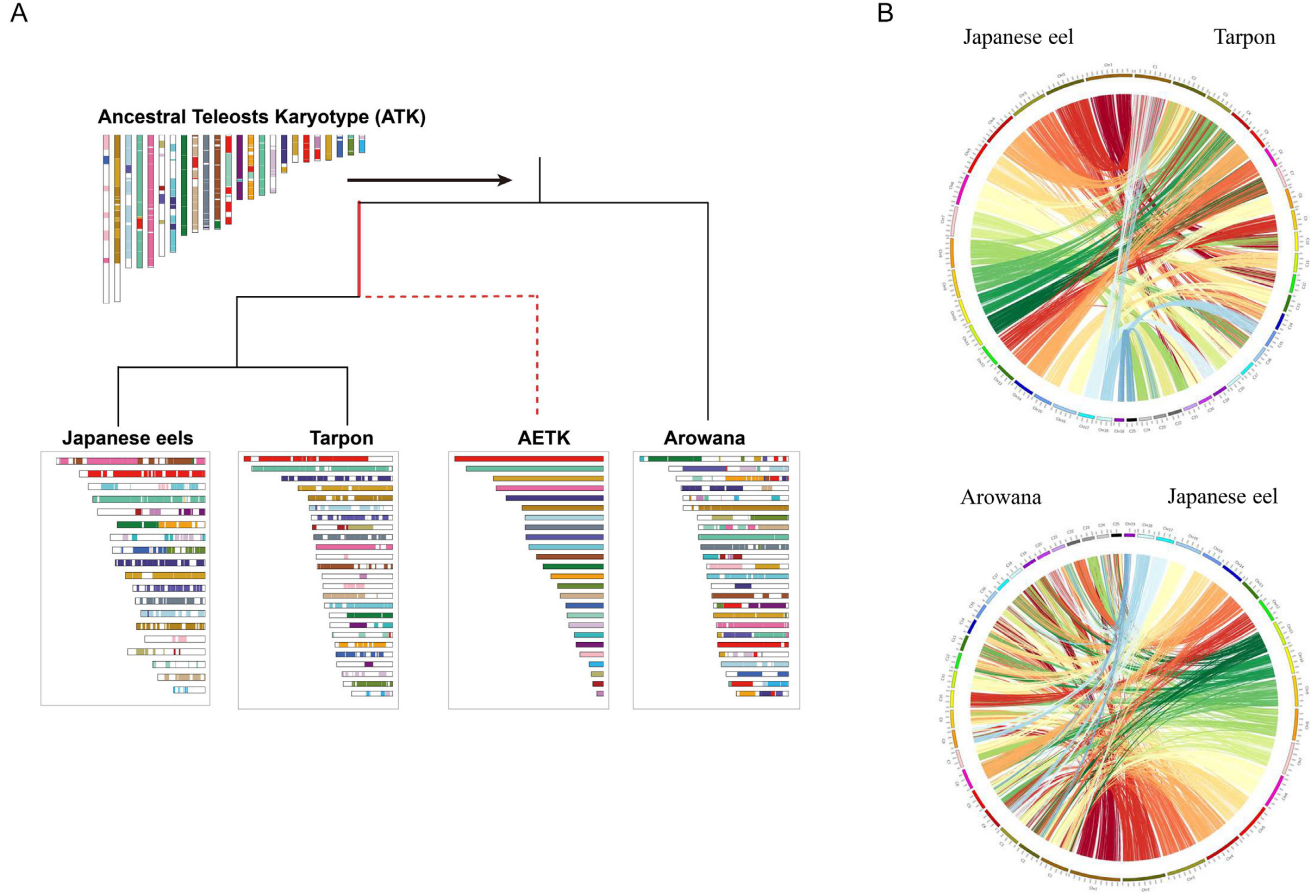
Reconstruction of protochromosomes for the common ancestor of teleosts (ATK) and eel/tarpons (AETK). (A) A model for the distribution of chromosomal segments in the genomes of ATK, arowana, AETK, Japanese eels, and tarpons. AETK is the common ancestor of tarpons and eels. The Circos plots indicate conservation of synteny between (B) Japanese eel and tarpon, as well as (C) arowana and Japanese eel.

When comparing chromosome numbers of the fishes that had all undergone 3R-WGD, the haploid chromosome number (*n*) is 25 for tarpons, arowana, and zebrafish; 24 for medaka; and 23 for Atlantic cod. Japanese eels have a lower haploid chromosome number (*n* = 19). To assess the extent of interchromosomal rearrangements in Japanese eels, we reconstructed the karyotype of the common ATK and AETK (Fig. 5A). According to our results, the ATK and AETK had 24 and 25 haploid chromosome numbers, respectively. The 14 AETK chromosomes (Chr 1, 3, 4, 6, 7, 9, 16–20, 22, 23, 24) had undergone 10-fusion and 10-fission to form the 14 tarpon chromosomes (Chr 1, 4–8, 10, 11, 18, 19, 21–23, 25) (Supplementary Table S13). The remaining 11 AETK chromosomes (Chr 2, 5, 8, 11, 25, 21, 15, 10, 12, 13, 14) correspond to those in tarpons (Chr 2, 3, 9, 12–17, 20, 24). This chromosome rearrangement resulted in the same haploid chromosome number (*n* = 25) in tarpons. Comparatively, the 19 AETK chromosomes (Chr 1, 2, 4, 7, 9–14, 16–21, 23–25) underwent 24-fusion and 18-fission to form the 13 chromosomes (Chr 1–8, 11, 13, 15–17) in Japanese eels (Supplementary Table S14). The remaining 6 AETK chromosomes (Chr 3, 5, 6, 8, 15, 22) correspond to the 6 chromosomes (Chr 9, 10, 12, 14, 18 & 19) in Japanese eels. This chromosome rearrangement resulted in the reduction of the chromosome number (*n* = 19) in Japanese eels, of which Chr 1, and Chr 3–7 rearrangements are unique to Japanese eels and might play a role in speciation. Japanese eel's chromosomes (Chr 2, 11, 15) were derived from AETK's (Chr 1, 3, 21) with slight rearrangements. The patterns of chromosome rearrangements in Chr 8, 13, and 16–17 of Japanese eels were comparable with Chr 21, 6, 8, and 19 in tarpons. Without rearrangement, Japanese eel's chromosomes 10, 14, and 19 were equivalent to AETK's chromosomes 3, 6, and 22. In addition, 3 chromosomes in the Japanese eel (Chr 9, 12, 18) derived directly from AETK's chromosomes (Chr 5, 8, 15), which also correspond to tarpon's chromosomes (Chr 3, 9, 15), respectively. Figure 5B and Supplementary Fig. 10 show the alignment of Japanese eel's chromosomes to tarpon's and arowana's chromosomes and highlight distinct conservation of orthologous segments.

## Discussion

In the past 10 years, the high-resolution whole-genome sequences of the teleosts, zebrafish [73], flatfish [74], killifish [75], salmon [76], and the nonteleost ray-finned fishes, including spotted gar [67], starlet sturgeon [77], the early ray-finned fishes (i.e., bichir, paddlefish, bowfin, and alligator gar) [78], and European eels [17, 79], were published. However, as the extant basal group of teleosts, a high-resolution genome assembly of Pacific *Anguilla* species was not achieved. Here, we report the high-quality chromosomal-level Japanese eel's genome for understanding the evolution of this extant basal group and providing the genome database for identifying adaptive and disease-resistant alleles.

The phylogenetic analysis of OR genes identified from the genome sequences of medaka, Atlantic cod, zebrafish, gar, coelacanth, and Australian ghost shark indicated that the delta (δ) and zeta (ζ) group genes in the freshwater eels expanded enormously, comprising about 86% of the entire gene family. Delta (δ) and ζ belong to the type I genes [78], which are specialized for detecting water-soluble odorants and are uniquely expressed in the water-filled lateral diverticulum of the nasal cavity [80, 81]. Consistently, a high number of δ transcripts were reported in European eels [82]. The mammalian type I (alpha group, α) and (gamma group, γ) genes detect airborne odor molecules. In teleost fishes, the group α genes are absent [78]. Interestingly, the group γ genes were found to have 26 in European and 7 in Japanese eels. Since eels can briefly live on land, they may have retained the group γ genes. The number of group β genes that detect airborne and water-soluble odor molecules was low in the freshwater eels but high in arowana (35) and spotted gar (20). The group eta (η) genes (type 2) is the third major OR gene group in the freshwater eels. The group η genes are mainly expressed in fishes and are absent in mammals [83].

The voltage-gated Ca^2+^ channels were the significantly expanded gene families in Japanese eels. Genome studies suggest that the cellular functions of voltage-gated ion channels emerged early in Metazoan evolution [84, 85] in determining physiology and behavior at the time of early divergence. It is probably associated with the physiological challenge of Japanese eels to maintain a narrow range of intrinsic Ca^2+^ during migration between waters with great variations of calcium contents. A gene expression study in marbled eel (*Anguilla marmorata*) showed high expression of voltage-gated Ca^2+^channels in brain, skin, and osmoregulatory tissues (i.e., gills, intestine, and kidneys) and its response to changes in water calcium levels [86]. Besides controlling Ca^2+^ homeostasis, Ca^2+^ signaling coordinates various physiological processes, including skeletal muscle contractions, nervous system activity, and cardiac and reproductive functions. The expanded gene families of thick myosin filament in skeletal muscle imply enhanced coordination of muscle contraction and performance [87], especially for this distinct clade of elongated bodies inhabiting a diverse range of habitats [88]. Additionally, the expanded gene families in lipoprotein receptor–related protein and the isocitrate and isopropyl malate dehydrogenases unravel the importance of these fundamental metabolic and morphogenetic functions in this lineage. Interestingly, lipoprotein receptor–related proteins first appeared during an evolutionary burst associated with the first multicellular organisms and are multifunctional receptors in the nervous system to modulate signals in brains [89, 90]. Isocitrate dehydrogenase is an important enzyme of carbohydrate metabolism, while isopropyl malate dehydrogenase is involved in leucine biosynthesis. Although Japanese eels underwent 3R-WGD, an additional TD and PD duplication was detected. These duplication events and genetic raw materials were provided to facilitate new adaptations to the changing environment [91]. The duplicated genes might have strengthened immune-related responses against different pathogens [92–94]. These evolutionary novelties could be attributed to changes in the ecological environment, challenging physiological fitness for adaptation [95]. Notably, the positive selection of immune-related genes indicates the adaptive advantages of the additional TD and PD duplication. Intriguingly, duplicated immune genes were also observed in salmon [96] and sturgeon [77].

The acquisition of evolutionary novelty by WGD duplication and the subsequent fate change of duplicated genes is necessary for phenotype alteration, environmental adaptation, and speciation [91]. The large-scale genomic reshaping after the third round of WGD affects evolutionary complexity and novelty in teleost fishes [97, 98]. It has been widely established that chromosomal numbers are the most fundamental genomic characteristic of an organism or a lineage [99]. Based on the hypothesis that genome duplication results in chromosomal rearrangements [100], understanding the rearrangement event in the eel genome may provide insight into the evolution of karyotype numbers at the base of the teleost evolutionary tree. The majority of fishes today have between 40 and 60 chromosomes (diploid number), while some commonly ancestral fishes are thought to have 48 chromosomes. Chromosome rearrangement and duplication have been the principal mechanisms involved in fish evolution, including the generation of new species and development of sex chromosomes. It is noted that freshwater fishes generally have a higher number of chromosomes (the modal diploid number = 54) than marine fishes (the modal diploid number = 48). It has been suggested that the higher number of chromosomes in freshwater fishes is related to a less stable freshwater environment with greater topographical barriers [101]. On the other hand, a large capacity for dispersal in marine environments would contribute to the homogenization of populations, reducing karyotype diversity [102]. Retrospectively, freshwater species seem to speciate more frequently than marine ones [103]. Interestingly, Japanese eels, although mostly freshwater dwellers, have a marine origin based on phylogenetic analysis of mitogenome sequences [104]. In a study of reconstructing the vertebrate ancestral genome to reveal dynamic genome reorganization, the 3R-WGD in the teleosts ancestor resulted in the number of chromosomes reaching a haploid number (*n*) of 26 [105]. Evolutionarily, chromosome numbers peak at *n* = 24 or 25 in extant teleost species. In this study, we reconstructed the ancestral protochromosomes AETK (*n* = 25) to describe the cross-species chromosome collinearity and underpin the lineage-specific genome reorganization. The chromosome number of *Anguilla*species (*n* = 19) was reduced as compared with *M. cyprinoides* (*n* = 25) and *S. formosus* (*n* = 25). The Anguilliformes is made up of 15 families with remarkable karyotypic diversity [106]. The haploid number ranges from 18 to 25, with a prevalence of *n* = 19 and 21. The *Anguilla* lineage underwent a significant structural rearrangement upon their divergence from the common ancestor of tarpons (*M. cyprinoides*). The fusion and fission of their chromosome structure were the primary drivers of reducing the haploid chromosome number to 19.

## Data Availability

The *A. japonica* whole genome sequencing and assembly are publicly available on NCBI databases under the accession number PRJNA852364. The gene models are available at Zenodo [107]. All supporting data are available in the *GigaScience* GigaDB database [108].

## Additional Files

**Supplementary Fig. S1**. The alignment plot shows our mitochondrial genomes assembled and GenBank ID AB038556.2 of Japanese eels.

**Supplementary Fig. S2**. Circos plot for the mitochondrial genome of Japanese eel. From outer to inner circles: protein-coding genes, rRNA, and tRNA; depth of Illumina short reads; and GC content.

**Supplementary Fig. S3**. Multiplatform Japanese eel genome assembly.

**Supplementary Fig. S4**. Genome comparison of Japanese (*A. japonica*) and European (*A. anguilla*) eels.

**Supplementary Fig. S5**. Venn diagram of gene annotation based on 5 databases (NR, InterPro, KEGG, SwissProt, and KOG).

**Supplementary Fig. S6**. KEGG-based gene function classification. The numbers represent how many genes are in the particular functions.

**Supplementary Fig. S7**. KOG-based gene function classification. The numbers represent how many genes are in the particular functions.

**Supplementary Fig. S8**. Length distribution of messenger RNA, CDS, exon, intron, and the number of exons in Japanese eel and the other species (*A. anguilla, A. rostrata, L. oculatus, M. cyprinoides*).

**Supplementary Fig. S9**. Ks distributions of syntenic paralogs and orthologs. Ks value distribution is used to identify genome duplication and speciation.

**Supplementary Fig. S10**. Comparative genomic analysis of Japanese eels (*A. japonica*), tarpons (*M. cyprinoides*), and arowanas (*S. formosus*).

**Supplementary Table S1**. Genome sequencing platforms for *A. japonica*.

**Supplementary Table S2**. A summary of contig statistics from the ONT long-read assembly.

**Supplementary Table S3**. A summary of contig statistics after assembly error correction.

**Supplementary Table S4**. Scaffolding by 10× linked reads, Bionano optical mapping, and Hi-C.

**Supplementary Table S5**. The completeness of *A. japonica* genome by BUSCO assessment.

**Supplementary Table S6**. Statistical results for repeat sequences.

**Supplementary Table S7**. A statistical analysis of the classification results for TE.

**Supplementary Table S8**. Functional annotation of predicted genes from *A. japonica*.

**Supplementary Table S9**. The completeness of *A. japonica* genes by BUSCO assessment.

**Supplementary Table S10**. The average length of exons in Japanese eel and the 8 related fish species.

**Supplementary Table S11**. GO enrichment analysis of the gene families expanded in the 3 freshwater eel genomes.

**Supplementary Table S12**. GO enrichment analysis of the gene families expanded in the *A. japonica* genome.

**Supplementary Table S13**. The karyotypes of *M. cyprinoides* (tarpons) and the common ancestor of eels and tarpons (AETK).

**Supplementary Table S14**. The karyotypes of *A. japonica* (Japanese eel) and the common ancestor of eels and tarpons (AETK).

giac120_GIGA-D-22-00177_Original_Submission

giac120_GIGA-D-22-00177_R1_Revision_1

giac120_GIGA-D-22-00177_Revision_2

giac120_Response_to_Reviewer_Comments_Original_Submission

giac120_Response_to_Reviewer_Comments_Revision_1

giac120_Reviewer_1_Report_Original_Submission Christiaan Henkel -- 8/9/2022 Reviewed

giac120_Reviewer_1_Report_Revision_1 Christiaan Henkel -- 10/6/2022 Reviewed

giac120_Reviewer_2_Report_Original_Submission Zhong Li -- 8/22/2022 Reviewed

giac120_Supplemental_Figures_and_Tables

## Abbreviations

AETK: ancestral eel/tarpon karyotype; ATK: ancestral teleosts karyotype; BLAST: Basic Local Alignment Search Tool; bp: base pair; BUSCO: Benchmarking Universal Single-Copy Orthologs; CLR: continuous long read; DSD: dispersed duplicate; Gb: gigabase; Kbp: kilobase pair; KEGG: Kyoto Encyclopedia of Genes and Genomes; Mb: megabase; Mbp: megabase pair; Mya: million years ago; NCBI: The National Center for Biotechnology Information; ONT: Oxford Nanopore; OR: olfactory receptor; ORF: open reading frame; PD: proximal duplicate; rRNA: ribosomal RNA; TD: tandem duplicate; TE: transposable element; TRD: transposed duplicate; tRNA: transfer RNA; WGD: whole-genome duplication; 2R-WGD: 2-round whole-genome duplication; 3R-WGD: 3-round whole-genome duplication; 4R-WGD: 4-round whole-genome duplication.

## Authors' Contributions

The experimental plan and sequencing strategy were designed by C.K.C.W., E.L.Z., A.O.L.W., K.P.L., and T.F.C. Samples were collected by A.H.M.N. and H.T.W. Bionano optical mapping and data analysis were conducted by C.Y.L.C., E.Y.C.C., and J.Z. The sequencing data for genome assembly were analyzed by E.L.Z., H.W., B.W., J.J., E.Y.C.C., and T.F.C. The manuscript was written by C.K.C.W., H.T.W., E.L.Z., H.W., and A.O.L.W.
