# Supplementary material for: A Chromosome-level assembly of the Japanese eel genome, insights into gene duplication and chromosomal reorganization
Source: Gigascience. 2022 Dec 8;11:giac120. doi: 10.1093/gigascience/giac120 (PMC9730501; doi:10.1093/gigascience/giac120)
Supplement: giac120_GIGA-D-22-00177_R1_Revision_1 [file giac120_giga-d-22-00177_r1_revision_1.pdf]

GigaScience

A Chromosome-level Assembly of the Japanese Eel Genome, Insights into Gene Duplication and Chromosomal Reorganization

--Manuscript Draft--

**Manuscript Number:**	GIGA-D-22-00177R1
**Full Title:**	A Chromosome-level Assembly of the Japanese Eel Genome, Insights into Gene Duplication and Chromosomal Reorganization
**Article Type:**	Data Note
**Funding Information:**	Southern Marine Science and Engineering Guangdong Laboratory (Guangzhou) (SMSEGL20SC02) — Not applicable<br>General Research Fund (Research Grant Council) (HKBU12162016) — Not applicable<br>Collaborative Research Fund, Research Grant Council (C4015-20E) — Prof. Chris Kong-Chu WONG
**Abstract:**	Japanese eels (Anguilla japonica) are commercially important species, harvested extensively for food. Currently, this and related species (American and European eels) are challenging to breed on a commercial basis. As a result, the wild stock is used for aquaculture. Moreover, climate change, habitat loss, water pollution, and altered ocean currents affect eel populations negatively. Accordingly, the International Union for Conservation of Nature lists Japanese eels as endangered and on its red list. Here we presented a high-quality genome assembly for Japanese eels and demonstrated that large chromosome reorganizations occurred in the events of third-round whole-genome duplications (3R-WRD). Several chromosomal fusions and fissions have reduced the ancestral protochromosomal number of 25 to 19 in the Anguilla lineage. A phylogenetic analysis of the expanded gene families showed that the olfactory receptors (group delta and zeta genes) and voltage-gated Ca2+-channels expanded significantly. Both gene families are crucial for olfaction and neurophysiology. Additional tandem and proximal duplications occurred following 3R-WGD to acquire immune-related genes for an adaptive advantage against various pathogens. The Japanese eel assembly presented here can be used to study other Anguilla species relating to evolution and conservation.
**Corresponding Author:**	Chris Kong-Chu WONG, PhD<br>Hong Kong Baptist University<br>Hong Kong, CHINA
**Corresponding Author Secondary Information:**	
**Corresponding Author's Institution:**	Hong Kong Baptist University
**Corresponding Author's Secondary Institution:**	
**First Author:**	Hongbo Wang
**First Author Secondary Information:**	
**Order of Authors:**	Hongbo Wang<br>Hin Ting WAN<br>Bin WU<br>Jianbo JIAN<br>Alice HM Ng<br>Claire Yik-Lok CHUNG<br>Eugene Yui-Ching CHOW

Powered by Editorial Manager® and ProduXion Manager® from Aries Systems Corporation

	Jizhou ZHANG
	Anderson OL Wong
	Keng Po LAI
	Ting Fung CHAN
	Eric Lu Zhang
	Chris Kong-Chu WONG
**Order of Authors Secondary Information:**	
**Response to Reviewers:**	We would like to thank the editor and both reviewers for their comments. We have addressed all the comments. New figures and revision have been made. Our reply to individual questions are listed in a document which has been attached (rebuttal letter). Thanks
**Additional Information:**	
**Question**	**Response**
Are you submitting this manuscript to a special series or article collection?	No
**Experimental design and statistics**<br><br>Full details of the experimental design and statistical methods used should be given in the Methods section, as detailed in our Minimum Standards Reporting Checklist. Information essential to interpreting the data presented should be made available in the figure legends.<br><br>Have you included all the information requested in your manuscript?	Yes
**Resources**<br><br>A description of all resources used, including antibodies, cell lines, animals and software tools, with enough information to allow them to be uniquely identified, should be included in the Methods section. Authors are strongly encouraged to cite Research Resource Identifiers (RRIDs) for antibodies, model organisms and tools, where possible.<br><br>Have you included the information requested as detailed in our Minimum Standards Reporting Checklist?	Yes

**Availability of data and materials**<br><br>All datasets and code on which the conclusions of the paper rely must be either included in your submission or deposited in publicly available repositories (where available and ethically appropriate), referencing such data using a unique identifier in the references and in the "Availability of Data and Materials" section of your manuscript.<br><br>Have you have met the above requirement as detailed in our Minimum Standards Reporting Checklist?	Yes

Powered by Editorial Manager® and ProduXion Manager® from Aries Systems Corporation

Manuscript

Click here to access/download;Manuscript;Manuscript-GigaScience-CLEAN.docx

ARTICLE (RESOURCES)

A Chromosome-level Assembly of the Japanese Eel Genome, Insights into Gene Duplication and Chromosomal Reorganization

Hongbo WANG[3], Hin Ting WAN[2], Bin WU[4], Jianbo JIAN[4], Alice HM NG[2], Claire Yik-Lok CHUNG[5], Eugene Yui-Ching CHOW[5], Jizhou ZHANG[5], Anderson OL WONG[1,6], Keng Po LAI[1,7], Ting Fung CHAN[1,5], Eric Lu Zhang[3*], Chris Kong-Chu WONG[1,2*]

[1]Southern Marine Science and Engineering Guangdong Laboratory (Guangzhou), [2]Croucher Institute for Environmental Sciences, Department of Biology, [3]Department of Computer Science, Hong Kong Baptist University, Hong Kong SAR; [4]BGI Genomics, BGI-Shenzhen, Shenzhen 518083, China; [5]School of Life Sciences, State Key Laboratory of Agrobiotechnology, Hong Kong Bioinformatics Centre, the Chinese University of Hong Kong, Hong Kong SAR; [6]School of Biological Sciences, the University of Hong Kong, Hong Kong SAR; [7]Key Laboratory of Environmental Pollution and Integrative Omics, Guilin Medical University, Guilin, PR China;

*Corresponding author,
Dr Eric Lu ZHANG
[3]Department of Computer Science,
Hong Kong Baptist University,
Hong Kong SAR
Email address: ericluzhang@comp.hkbu.edu.hk

Dr Chris KC Wong
[1]Southern Marine Science and Engineering Guangdong Laboratory (Guangzhou),
[2]Croucher Institute for Environmental Sciences,
Department of Biology,
Hong Kong Baptist University,
Hong Kong SAR
Email address: ckcwong@hkbu.edu.hk.

ABSTRACT

Japanese eels (*Anguilla japonica*) are commercially important species, harvested extensively for food. Currently, this and related species (American and European eels) are challenging to breed on a commercial basis. As a result, the wild stock is used for aquaculture. Moreover, climate change, habitat loss, water pollution, and altered ocean currents affect eel populations negatively. Accordingly, the International Union for Conservation of Nature lists Japanese eels as endangered and on its red list. Here we presented a high-quality genome assembly for Japanese eels and demonstrated that large chromosome reorganizations occurred in the events of third-round whole-genome duplications (3R-WRD). Several chromosomal fusions and fissions have reduced the ancestral protochromosomal number of 25 to 19 in the Anguilla lineage. A phylogenetic analysis of the expanded gene families showed that the olfactory receptors (group δ and ζ genes) and voltage-gated Ca^{2+}-channels expanded significantly. Both gene families are crucial for olfaction and neurophysiology. Additional tandem and proximal duplications occurred following 3R-WGD to acquire immune-related genes for an adaptive advantage against various pathogens. The Japanese eel assembly presented here can be used to study other Anguilla species relating to evolution and conservation.

KEYWORDS

Anguilla japonica, Phylogenomics, Gene Expansion, olfactory receptors, Ca^{2+}-channels

INTRODUCTION

Fishes are highly diverse species living in many ecological habitats, including freshwater, estuarine, and the ocean (Hughes *et al.* 2018). Over 99% of fish species are known to be stenohaline, inhabiting freshwater or marine environments. In contrast, euryhaline fishes are diadromous, migrating between freshwater and saltwater environments in their life cycles (Gross *et al.* 1988). Catadromous fishes like eels spawn in the sea and migrate to inland freshwater to grow and mature. Eels are ecologically and economically essential, serving as indicators of the healthiness of coastal environments and resources in aquaculture. The fish are not bred in captivity (Jehannet *et al.* 2021). In current practices, glass eels (juvenile life stage) are captured from the wild and raised on farms. Over 90 % of freshwater eels consumed worldwide are farm-raised. Since the 1960s, catches of Anguillid eels, like European and Japanese eels, have declined by over 50-80 %. In a 2014 report from the International Union for Conservation of Nature (IUCN), the American, European, and Japanese eels have been listed as at high risk of extinction. The decline in eel populations is abetted by soaring demand from global markets. In addition, overfishing, habitat loss, dams (Piper *et al.* 2015), water pollution (Geeraerts & Belpaire 2010), parasites (Hein *et al.* 2014), eel larvae predation by mesopelagic fishes (Jensen *et al.* 2018), climate change, and altered ocean currents (Chang *et al.* 2018) are known to cause population decline.

From the evolutionary perspective, eels are among the extant basal groups of teleost ray-finned fishes after the 3-round whole genome duplication (3R-WGD) (Near *et al.* 2012). The non-teleost teleost ray-finned fishes, including holostei (bowfin, gar), chondrostei (sturgeon, paddlefish, starlet), and cladistia (bichir, ropefish), diverged from lobe-finned fishes (coelacanth, lungfish) about 450 million years ago (Hurley *et al.* 2007). Comparing eels with other ray-finned fishes would shed light on fish evolution. In 2012, the first draft genome sequences of the Japanese eels (genome size 1.15 Gb, N50 of 52.8 Kbp, number of scaffolds 323,776) and European eels (0.923 Gb, N50 of 78Kbp) were published (Henkel *et al.* 2012a; Henkel *et al.* 2012b). Afterward, double-digested restriction-site associated DNA sequencing (ddRAD-seq) was applied to construct a linkage map of the

Japanese eel, generating 19 linkage groups for subsequent quantitative trait loci analysis (Kai *et al.* 2014). The Japanese eel's draft genome's annotation was further enhanced using transcriptome data (Liu *et al.* 2016) and the phylogenetic analysis of rhodopsin genes in the Japanese eel (1.15 Gb, N50 of 472 Kbp, number of scaffolds 195,366) (Nakamura *et al.* 2017). Moreover, the genome assembly of the European eel was improved to 0.979 Gb, N50 of 57.2 Mbp, number of scaffolds 54 (Jansen *et al.* 2017) and to 1.03 Gb, N50 of 55.98 Mbp, number of scaffolds 1,466 (Parey *et al.* 2022). A draft genome of the American eel (with a total size of 1.41 Gb, N50 of 86.6 Kbp, number of scaffolds 79,209) was published in 2017, and 26,564 genes were annotated (Pavey *et al.* 2017). In 2019, the assembly of a Japanese genome of 1.18 Gb (Chen *et al.* 2019) was improved with 256,649 contigs, 41,687 scaffolds, and a scaffold N50 of 1.03Mbp. Currently, only the draft genome is available for Japanese eels. This study aimed to provide high-quality genome assemblies and understand karyotype evolution in early ray-finned fishes. The genome-scale data can provide ecological and conservation information by identifying adaptive and disease-resistant alleles.

MATERIALS AND METHODS

Genome Sequencing. A market-purchased female Japanese eel, *Anguilla japonica*, was kept in a freshwater tank for a week with aeration. Blood and muscle samples were taken from the fish, snapped frozen in liquid nitrogen, and then stored at -80°C. Genomic DNA was extracted from the blood samples. DNA sequencing data were generated by different platforms, including Oxford Nanopore (ONT) long reads, PacBio continuous long reads (CLR), Illumina short reads, Illumina mate-pair reads, 10X Chromium linked-reads, DNase Hi-C (Omni-C), and Bionano optical mapping.

The library for ONT long-read sequencing was prepared using the Ligation Sequencing Kit (LSK109) and sequenced using the Nanopore PromethION P48 sequencer with the flow cells (R9.4.1) and the basecaller version Guppy 3.2.10. For PacBio CLR sequencing, the SMRTbell templates were prepared using Sequel Binding Kit 1.0 and sequenced on the PacBio Sequel System. For Illumina short reads and mate-pair sequencing, the libraries were prepared using TruSeq DNA PCRFree Kit and Nextera Mate Pair Library Preparation Kit (gel plus), respectively. They were sequenced with 2×150bp reads on an Illumina HiSeq X Ten instrument. The library for linked reads was prepared by a 10X Genomics Chromium system with Chromium Genome library (v2) and sequenced with 2×150bp reads on an Illumina NovaSeq 6000 instrument. Dovetail Omni-C Kit was used for Hi-C library preparation, which used NEBNext Ultra enzyme and Illumina-compatible adapters. Biotin-containing fragments were isolated using streptavidin beads before PCR enrichment. The library was sequenced with 2×150bp reads on an Illumina HiSeqX platform. The Bionano optical mapping was generated by three enzymes, two from Irys (Nt.BspQI and Nb.BssSI) and one from Saphyr (DLE1). We stretched and captured the images of fluorescently labeled DNA molecules in Irys and Saphyr G1.2 chips. The labeling distances were extracted from the images and recorded into the raw molecule files. Molecules over 150 kbp were assembled into consensus maps using Bionano Solve for further analysis (**Supplementary Table 1**).

Genome Assembly on ONT Long Reads. MitoZ software (v2.4) (Meng *et al.* 2019) was used to assemble and annotate the mitochondrial genome of the Japanese eel. We assembled ONT long reads using Canu (v2) (Koren *et al.* 2017), Wtdbg2 (v2.5) (Ruan & Li 2020), and Flye (v2.71) (Kolmogorov *et al.* 2019) separately and merged their contigs using Quickmerge (Chakraborty *et al.* 2016) to achieve a balance between contig N50 and percentage of complete genes (PCGs) in vertebrate species. We used Racon (v1.4.16) (Vaser *et al.* 2017) for two rounds and Medaka (v1.6.1) (https://github.com /nanoporetech/medaka) for one round to self-correct assembly errors using ONT reads, respectively. The PacBio CLR was then incorporated for error correction using Racon for two rounds. As the last step, we further improved the assembly by integrating Illumina short-reads and mate-pair libraries using Pilon (v1.23) (Walker *et al.* 2014) for two rounds.

Scaffolding on 10x linked-reads, Bionano and Hi-C. We applied Tigmint (v1.1.2) (Jackman *et al.* 2018) and ARKS (v1.0.3) (Coombe *et al.* 2018) to correct misassembled contigs and linking contigs into scaffolds according to the shared barcodes from 10x linked-reads. We used OMGS (Pan *et al.* 2020) to integrate three enzymes used in Bionano optical mapping for scaffolding. We further extended the scaffolds using 3D-DNA (180419) (Dudchenko *et al.* 2017) based on the Hi-C data from Dovetail Omni-C library and refined the scaffolds manually by JuiceBox (v1.11.08) (Durand *et al.* 2016) to extend the scaffolds to the corresponding chromosome scale.

Tandem Repeats and Transposable Elements Annotation. Tandem Repeats Finder (v4.09) (Benson 1999) was applied to annotate tandem repetitive sequences. We utilized homolog-based and *de novo* approaches to annotate transposable elements (TEs) in the Japanese eel genome. For the homolog-based approach, RepeatMasker (v4.0.7) (Tarailo-Graovac & Chen 2009) and RepeatProteinMask (v4.0.7) (http://www.repeatmasker.org/cgibin/RepeatProteinMaskRequest) were used to identify the repeats by aligning the known TE sequences from RepBase (v21.12) database (Jurka *et al.* 2005) to the genome. LTR_FINDER (v1.06) (Xu & Wang 2007) was used to infer long terminal repeat retrotransposons. For the *de novo* approach, RepeatModeler (v1.0.8) (http://www.repeatmasker.org/RepeatModeler) was used to detect the TE families and repeat

boundaries by integrating three complementary *de novo* repeat finding programs. RepeatMasker collected the union of these tools' results and annotated the genome accordingly.

Genes and their Functional Annotation. Three types of methods were used to annotate the protein-coding genes in the genome, including *de novo*, homology-based, and transcriptome-based annotations. Maker (v2.31.8) (Holt & Yandell 2011) was adopted for homology annotation using the protein sequences from the five closely related species, including European eel (*Anguilla Anguilla),* zebrafish (*Danio rerio*), Indo-Pacific tarpons (*Megalops cyprinoides*), Asian arowana (*Scleropages formosus*), and spotted gar (*Lepisosteus oculatus*), based on the phylogeny of teleost fishes (Bian *et al.* 2016).

De novo annotation was performed using Augustus (v3.2.1) (Stanke *et al.* 2006) and SNAP (v1) (Johnson *et al.* 2008) by training a model using 3,000 complete genes obtained from homology prediction. Transcriptome annotation was performed by aligning RNA-seq data (Bioproject: PRJNA578238) to the genome with HISAT2 (v2.1.0) (Kim *et al.* 2015) and assembling transcript sequences with Trinity (v2.10.0) (Haas *et al.* 2013). Pasa_lite (https:// github.com/ PASApipeline/PASA_Lite) was used to correct assembly errors to obtain the final transcripts. Maker (v2.31.8) was further applied to integrate the three annotations, followed by the second round of homology annotation to refine the final gene set.

Gene functional annotation was performed by aligning the predicted gene sequences to protein sequences using BLAST (v2.2.31) (Altschul *et al.* 1990) in the six databases, including NCBI Non-Redundant Protein Sequence (NR), Kyoto Encyclopedia of Genes and Genomes (Kanehisa & Goto 2000), SwissProt (Boeckmann *et al.* 2003), KOG (Tatusov *et al.* 2003), Gene Ontology (Ashburner *et al.* 2000), and TrEMBL (Uniprot version 2020-06). We further searched the secondary structure domain database for gene function prediction using InterProscan (Zdobnov & Apweiler 2001).

Evaluation of Genome Assembly and Gene annotation. BUSCO (v5.1.2) (Simao *et al.* 2015) was used to evaluate genome assembly and gene annotation by calculating the

completeness of single-copy orthologs. We selected the Ray-finned Fish single-copy orthologs direct homologous gene database actinopterygii_odb10 (which contains 3640 core single-copy direct homologous gene proteins), the closest relative to the Japanese eel in the OrthoDB database (https://www.orthodb.org/) to compare.

Annotation of Conserved Noncoding Elements. tRNAscan-SE 1.3.1 (Lowe & Eddy 1997) was used to identify tRNA sequences in the genome families. We annotated the rRNA sequences by aligning the conserved rRNA sequences from the five closely related fish species (European eel, zebrafish, tarpons, arowana, and spotted gar) to the genome using BLASTN (Zhang *et al.* 2000). The microRNAs and snRNAs were annotated by aligning the corresponding sequences from Rfam (v12) (Griffiths-Jones *et al.* 2005) to the genome.

Phylogenetic Analysis, Gene Expansion, and Gene Contraction. OrthoMCL (v2.0) (Li *et al.* 2003) (http://orthomcl.org/orthomcl/) was used to identify gene families by grouping orthologous proteins. We applied the maximum likelihood method (Guindon & Gascuel 2003) and RAxML (v2.2.3) (Stamatakis 2014) (http://sco.h-its.org/exelixis/web/software/raxml/index. html) to reconstruct the phylogenetic tree using four-fold degenerate sites (4DTv) in single-copy orthologs from the 12 fish species, including *Anguilla rostrata* (American eel, GenBank assembly: GCA_001606085.1), *Anguilla anguilla* (European eel, GCA_013347855.1), *Anguilla japonica* (Japanese eel), *Megalops cyprinoides* (tarpons, GCA_013368585.1), *Scleropages formosus* (arowana, GCA_900964775.1), *Gadus morhua* (Atlantic cod, GCA_902167405.1), *Oryzias latipes* (medaka, GCA_002234675.1), *Danio rerio* (zebrafish, GCA_000002035.4), *Lepisosteus oculatus* (spotted gar, GCA_000242695.1), *Erpetoichthys calabaricus* (reed fish, GCA_900747795.2), *Latimeria chalumnae* (coelacanth, GCA_000225785.1) and *Callorhinchus milii* (Australian ghost shark, GCA_000165045.2). We estimated the divergence times for single-copy orthologos using mcmctree (http://abacus.gene.ucl.ac.uk/ software/paml.html) in PAML package (v4.8a) (Yang 2007) based on the predefined times from TimeTree website (http://www.timetree.org/) [*Danio rerio* with *Oryzias latipes* (180.0 - 264.0 Mya), *Megalops cyprinoides* with *Anguilla anguilla* (162.2 - 197.3 Mya), *Callorhinchus milii* with *Danio rerio* (442.7 - 515.5 Mya) and *Erpetoichthys calabaricus* with *Danio rerio*

(381.0 - 407.0 Mya)]. To estimate gene family expansion and contraction, we used CAFÉ (v4.2.1) (De *et al.* 2006) (http://sourceforge.net/projects/cafehahnlab/) to model gene expansions and contractions, as well as the divergence times.

Identification of olfactory receptor (OR) genes. We identified OR genes using the pipeline described in Github (https://github.com/MaximePolicarpo/Olfactory_receptor_ genes) (Policarpo *et al.* 2021), while candidate genes were filtered via the NR database. The OR gene identified in a previous study (Niimura 2009) was used as a query sequence. TBLASTN (v2.2.26) (Gertz *et al.* 2006) was used to identify genomic regions containing OR genes in the 10 fish species (European eel, Japanese eel, tarpons, arowana, medaka, Atlantic cod, zebrafish, spotted gar, coelacanth, and Australian ghost shark). Only the non-overlapping BLAST hits regions were extracted. The 1kb upstream and downstream flanking regions were used as the input to EMBOSS (v6.6.0) (Rice *et al.* 2000). Using EMBOSS, we generated Open Reading Frames (ORFs), translated the ORFs into protein sequences, and then ran BlastP (v2.2.26) to remove sequences that did not match genes already known in SwissProt and NR. InterProscan was used to determine the secondary structures of the predicted OR genes. Some genes were filtered due to lacking the seven transmembrane domains. The maximum likelihood phylogenetic tree was reconstructed using IQ-TREE (v2.2.0.3) (Nguyen *et al.* 2015) based on the multiple sequencing alignments on the OR gene sequences with MAFFT (v7.505) (Katoh & Standley 2013).

Genome evolution analysis. MCscanX (v1.5.1) (Wang *et al.* 2012) and macrosynteny visualization (jcvi) were used to screen for collinear blocks with at least 30 genes (Tang *et al.* 2008) in *Anguilla japonica, Anguilla anguilla, Anguilla rostrata, Megalops cyprinoides, and Lepisosteus oculatus*. The numbers of non-synonymous substitutions (Ka) and synonymous substitutions (Ks) were calculated using KaKs_calculator2.0 (Wang *et al.* 2010). In addition, we calculated 4dTv values to estimate the WGD events in the Japanese eel genome. We identified gene duplicates in the genomes of Japanese eel, zebrafish, arowana, medaka, and Atlantic cod using the DupGen_finder pipeline (Qiao *et al.* 2019), using spotted gars as an outgroup. It classified gene duplication patterns into five categories: whole genome duplications, tandem duplications, proximal duplications (non-tandem

duplications that are separated by 10 genes on the same chromosome), transposable duplications, and scattered duplications (duplications other than the four categories mentioned above).

Ancestral Chromosome Reconfiguration. Ancestral eel/tarpon karyotype (AETK) was constructed using *Anguilla japonica* (Japanese eel), *Megalops cyprinoides* (tarpon), and *Scleropages formosus* (arowana, outgroup). The ancestral teleosts karyotype (ATK) was constructed using zebrafish, *Scleropages formosus* (arowana), and *Lepisosteus oculatus* (spotted gar, outgroup) (Braasch *et al.* 2016). This was implemented using BLASTP (States & Gish 1994) to obtain homologous gene pairs between species. The default parameters of MCScanX were then applied to obtain the collinear blocks of chromosomes between species. Finally, the karyotype of the ancestor was constructed using ANGeS (v1.01) (Chauve & Tannier 2008).

RESULTS

Genome Assembly and Annotation. In this study, MitoZ software was used to assemble and annotate the mitochondrial genome (16.686Kb) of our sample to confirm the species' identity (**Methods**). The data matched with the Japanese eel mitochondrial genome (GenBank ID AB038556.2) of the NR database from NCBI (**Supplementary Fig 1&2**). We hierarchically integrated the sequencing data from different platforms to characterize their strength in *de novo* assembly and annotation (**Supplementary Fig. 3**). The draft genome was generated using ONT contigs followed by error correction and scaffolding based on the genomic spans of different sequencing technologies (Ghurye & Pop 2019) (**Methods**). A high-quality Japanese female eel's reference genome was then obtained through the integration of ONT long reads (234x, 239.64Gb), PacBio CLR (261x, 267Gb), 10x Chromium linked-reads (313x, 319.7Gb), Hi-C data (48x, 48.99 Gb), Illumina short-reads (148x, 151.89Gb) and mate-pair reads (127x, 130.5Gb). The contigs from ONT long reads resulted in a significantly improved N50 (25.82Mb) without losing many complete genes (54.6%) (**Supplementary Table 2**). With reduced assembly errors, the percentage of complete genes increased from 54.6% to 90.1%, indicating a higher base quality (**Supplementary Table 3**). For scaffolding, 10x linked-reads, Bionano, and Hi-C data were used sequentially according to fragment length to increase assembly continuity and assign scaffolds to 19 chromosomes (**Fig. 1**, **Supplementary Fig 3,** and **Supplementary Tables 4**). As a result, the genome size is 1.028Gb, the contig N50 is 21.48Mb, and the scaffold N50 is 58.7Mb. The chromosome lengths range from 19.93Mb to 94.28Mb. According to *actinopterygii_odb10* in the BUSCO database, 94% of the single-copy direct homologs in the Ray-finned Fishes were assembled in Japanese eels (**Supplementary Table 5**). The repeat elements accounted for 30.49% of the whole genome (**Supplementary Tables 6)**. The TEs were excluded from gene annotation (**Supplementary Tables 7**). Japanese eels have a higher percentage (30.49%) of repetitive sequences, which may explain their larger genome, compared to European eels (*Anguilla anguilla* 0.979 Gb) (Jansen *et al.* 2017). Even so, the Japanese and European eels have a

1:1 correspondence pattern of chromosomes and 19,325 homologous genes, demonstrating their matching structure (**Supplementary Fig 4**).

By combining gene annotations from homology, *de novo*, and transcriptome annotations (**Methods**), we identified 29,982 coding genes (**Table 1**). We functionally annotated 97.44 % (29,219) of these genes (**Supplementary Table 8**) using the publicly available databases (**Methods**). Additionally, 21,606 genes were annotated by all five major protein databases (**Supplementary Fig 5)**, with signal transduction pathways most abundant in KEGG **(Supplementary Fig 6)** and KOG **(Supplementary Fig 7)**. BUSCO analysis showed that 94.7 % of the single-copy orthologs could be found in the ray-finned fish single-copy direct homology gene database actinopterygii_odb10 (**Supplementary Table 9**). The protein-coding genes in Japanese eels have an average length of 10.2kbp and contain approximately 9 exons (**Table 1**), which have an average length of 1.6kbp (**Supplementary Table 10**). The gene structure of Japanese eels is similar to those of four closely related species (**Supplementary Fig 8**). The genome assembly has a greater number of predicted genes (29,982 genes) than the Atlantic species, European (25,903 genes), and America (26,565 genes) eels. Additionally, 17,095 noncoding RNAs were predicted, including 1,042 transfer RNAs (tRNAs), 1,771 ribosomal RNAs (rRNAs), and 3,974 microRNAs in Japanese eels.

Phylogenomics and demographic history. The orthology analysis of 12 species' coding genes identified 21,653 gene family clusters. *Anguilla japonica*'s genome contains 29,982 coding genes, including 3347 single-copy orthologs, 8204 multiple-copy orthologs, 233 unique paralogs, 12,662 other orthologs, and 5536 unclustered genes. A phylogenetic tree was reconstructed by identifying the fourfold synonymous third-codon transversion (4dTv) loci in the 1,131 single-copy orthologs from the 12 fish species (**Fig 2**). American and European eels diverged from their ancestors about 27.0 million years ago (MYA). With a divergence time of approximately 44.1 MYA, the Japanese eel was distant from the Atlantic eel species. Compared with the three freshwater eels (Anguilliformes) and tarpons (Elopiformes), the members of the Order Elopomorpha, their common ancestor, diverged 196.1 MYA. Elopomorpha and Osteoglossomorpha (i.e., arowana) are the closest

evolutionary relatives at the basal branch of teleosts (Parey *et al.* 2022), separating 240.9 MYA. *Gadiformes* (e.g., Atlantic cod) and *Cypriniformes* (e.g., medaka, zebrafish) diverged from the Eloposteoglossocephala clade at 262.5 MYA. Above are fish groups that had undergone three rounds of whole-genome duplication (3R-WGD). Compared to the outgroups, spotted gars, reed fish, coelacanths, and Australian ghost sharks underwent only 2R-WGD.

Expanded Gene Families and Gene duplication. The expansion and contraction of gene families reflect the evolution of organisms' adaptations to their environments. Ortholog analysis of genes from the 12 species (**Methods**) identified 21,652 gene family clusters. By removing gene families with too many (≥ 200) or too few (≤ 2) genes, we achieved 129,862 genes to evaluate the expansion and contraction of gene families (**Fig 2**). Compared to the nine other species (**Methods**), the three freshwater eels had expanded 771 and contracted 467 gene families, resulting in an increase of 919 and loss of 531 genes, respectively (**Supplementary Table 11**). Among those, the three freshwater eel species exhibited a significant expansion in the olfactory receptor (OR) gene family, which is crucial for detecting odor molecules under varying environmental conditions. A retrospective analysis of the OR receptors across 10 species' genomes was performed, and seven types of OR receptors were identified [alpha (α), beta (β), gamma (γ), delta (δ), epsilon (ε), zeta (ζ) and eta (η)] based on a previous study (Niimura 2009). Compared to other fish species, the Japanese eels had a significantly higher number of OR genes (394) (**Fig 3**), located on the four chromosomes - Chr4 (2 genes), Chr9 (153 genes), Chr11 (1 gene), and Chr12 (238 genes). Similarly, the European eel contains 392 OR genes. The δ and ζ genes are the major OR genes in the eels.

Comparing the Japanese eel to the other 11 species, 433 gene families increased, with a total increase of 551 genes. On the other hand, a total of 943 genes were lost from 782 gene families (**Supplementary Table 12**). It is interesting to note that Ca^{2+} and K^{+} channel families were identified. Calcium and potassium play significant roles in neuronal excitability, muscle contraction, fertilization, and energy metabolism. Interestingly, the other expanded gene families include (i) the assembly of thick myosin filament in skeletal

muscle, (ii) lipoprotein receptor-related protein (metabolic and morphogenetic pathways), and (iii) isocitrate and isopropyl malate dehydrogenases family (carbohydrate and amino acid metabolism).

It was reported that freshwater eels (European and Japanese) had many paralogous pairs after splitting from the Osteoglossomorpha lineage (Rozenfeld *et al.* 2019). The observation suggested 4R-WGD or lineage-specific re-diploidization in some duplicated genomic regions. We studied the distribution of 4dTv and Ks values of genome-wide direct homologous gene pairs in Japanese eels, European eels, and tarpons. There were 4dTv values of 0.402, 0.386, and 0.317 for *A. japonica, A. Anguilla, and M. cyprinoides*, respectively (**Fig 4A** and **Supplementary Fig 9**). Additional WGD events were not detected. We also compared the syntenic blocks at Hox A-D loci with those in spotted gar (2R-WGD) and zebrafish (3R-WGD) (**Fig 4B**). By identifying ohnolog pairs using collinear blocks of 10 genes, the Japanese eel's genome has eight clusters of Hox loci on chromosomes 1, 2, 3, 8, 11, 13, 15, and 17. In contrast, spotted gar has four clusters on chromosomes 4, 11, 12, and 13. Zebrafish underwent 3R WGD with 7 Hox gene clusters (lacking HoxDb) (Amores *et al.* 1998). We found that six (HoxAa, HoxAb, HoxBa, HoxCa, HoxCb, and HoxDa) out of seven Hox clusters of zebrafish exhibit ohnolog pairs with eels. Because zebrafish HoxBb gene clusters contain only four genes, eel HoxBb and zebrafish HoxBb did not show the ohnolog pair. Collectively, the data do not support the presence of 4R-WGD in Japanese.

There are 21,249 duplicated genes identified among the 29,982 coding genes in the Japanese eel genome. Based on their duplication patterns, DupGen_finder (**Methods**) classified the duplicated genes into five categories, (i) 9,890 whole-genome duplicates (WGD, 46.54%), (ii) 1,420 tandem duplicates (TD, 6.68%), (iii) 768 proximal duplicates (PD, 3.61%), (iv) 3,975 transposed duplicates (TRD, 18.71%), and (v) 5,196 dispersed duplicates (DSD, 24.45%). We then calculated the Ks and Ka/Ks values for these five gene categories. Ks distribution indicates that TD and PD revealed additional duplication post-3R-WGD **(Fig 4C)**. In addition, both TD and PD duplicates exhibited high Ka/Ks ratios, indicating high selection pressure, which was probably related to environmental adaptation.

TD and PD duplicated genes are mainly involved in immune responses (e.g., the production of interleukin-8, virus and biotic stress, somatic hypermutation of immunoglobulin genes, diversification and production of immunoglobulins and immunoreceptors) **(Fig 4D)**. Nonetheless, WGD was associated with 32.98 % of the total number of coding genes (29,982) in Japanese eels. Gene duplications in other fish species were also analyzed using the DupGen_finder pipeline (Qiao et al. 2019) and compared. Japanese eels were found to share the same level of WGD duplication of coding genes as arowana (37.60%), as both are extant members of the basal teleost group. However, it differs from the majority of teleosts, such as medaka (6.09%) zebrafish (9.51%), and Atlantic cod (4.68%). In Japanese eel, these duplicated gene functions were associated with neuronal (dendrites, synapses, neuron projections, obsolete synapses) and cell-cell junctions (cellular periphery, cell junctions, integral components of plasma membranes, obsolete plasma membranes, and cell projections). TRD shows a similar profile of changes. In DSD, duplication genes function in microtubules, reproduction (oocyte fate determination, fertilization), and ATP metabolism.

Evolution of chromosome number in Japanese eels. When comparing chromosome numbers of the fishes that all undergone 3R-WGD, the haploid chromosome number (n) is 25 for tarpons, arowana, zebrafish; 24 for medaka; 23 for Atlantic cod. Japanese eels have a lower haploid chromosome number (n = 19). To assess the extent of inter-chromosomal rearrangements in Japanese eels, we reconstructed the karyotype of the common ancestral teleosts karyotype (ATK) and ancestral eel-tarpon karyotype (AETK) (**Fig 5A**). According to our results, the ATK and AETK had 24 and 25 haploid chromosome numbers, respectively. The 14 AETK’s chromosomes (Chr1, 3, 4, 6, 7, 9, 16-20, 22, 23, 24) undergone 10-fusion and 10-fission to form the 14 tarpon’s chromosomes (Chr1, 4-8, 10, 11, 18, 19, 21-23, 25) (**Supplementary Table 13**). The remaining 11 AETK's chromosomes (Chr2, 5, 8, 11, 25, 21, 15, 10, 12, 13, 14) correspond to those in tarpons (Chr2, 3, 9, 12-17, 20, 24). This chromosome rearrangement resulted in the same haploid chromosome number (n = 25) in tarpons. Comparatively, the 19 AETK’s chromosomes (Chr1, 2, 4, 7, 9-14, 16-21, 23-25) underwent 24-fusion and 18-fission to form the 13

chromosomes (Chr1-8, 11, 13, 15-17) in Japanese eels (**Supplementary Table 14**). The remaining 6 AETK's chromosomes (Chr3, 5, 6, 8, 15, 22) correspond to the 6 chromosomes (Chr9, 10, 12, 14, 18 & 19) in Japanese eels. This chromosome rearrangement resulted in the reduction of the chromosome number (n = 19) in Japanese eels. Of which, Chr1, and Chr3-7 rearrangements are unique to Japanese eels and might play a role in speciation. Japanese eel's chromosomes (Chr2, 11, 15) were derived from AETK's (Chr1, 3, 21) with slight rearrangements. The patterns of chromosome rearrangements in Chr8, Chr13, and Chr16-17 of Japanese eels were comparable with Chr21, Chr6, Chr8, and Chr19 in tarpons. Without rearrangement, Japanese eel's chromosomes 10, 14 & 19 were equivalent to AETK's chromosomes 3, 6, and 22. In addition, there were three chromosomes in the Japanese eel (Chr9, Chr12, & Chr18) derived directly from AETK's chromosomes (Chr5, Chr8, & Chr15), those also corresponding to tarpon's chromosomes (Chr3, Chr9, & Chr15), respectively. **Figure 5B** and **Supplementary Fig 10** show the alignment of Japanese eel's chromosomes to tarpon's and arowana's chromosomes and highlight distinct conservation of orthologous segments.

DISCUSSION

In the past 10 years, the high-resolution whole-genome sequences of the teleosts, flatfish, zebrafish (Howe *et al.* 2013), flatfish (Chen *et al.* 2014), killifish (Valenzano *et al.* 2015), salmon (Lien *et al.* 2016), and the non-teleost ray-finned fishes, including spotted gar (Braasch *et al.* 2016), starlet sturgeon (Du *et al.* 2020), the early ray-finned fishes (i.e., bichir, paddlefish, bowfin & alligator gar)(Bi *et al.* 2021) and European eels (Rhie *et al.* 2021; Parey *et al.* 2022) were published. However, as the extant basal group of teleosts, a high-resolution genome assembly of Pacific Anguilla species was not achieved. Here, we report the high-quality chromosomal-level Japanese eel's genome for understanding the evolution of this extant basal group and providing the genome database for identifying adaptive and disease-resistant alleles.

The phylogenetic analysis of olfactory receptor (OR) genes identified from the genome sequences of medaka, Atlantic cod, zebrafish, gar, coelacanth, and Australian ghost shark indicated that the delta (δ) and zeta (ζ) group genes in the freshwater eels expanded enormously, comprising about 86% of the entire gene family. Delta (δ) and ζ belong to the type I genes (Bi *et al.* 2021), which are specialized for detecting water-soluble odorants and are uniquely expressed in the water-filled lateral diverticulum of the nasal cavity (Freitag *et al.* 1995; Glusman *et al.* 2000). Consistently, a high number of δ transcripts was reported in European eels (Churcher *et al.* 2015). The mammalian type I (alpha group, α) and (gamma group, γ) genes detect airborne odor molecules. In teleost fishes, the group α genes are absent (Bi *et al.* 2021). Interestingly, the group γ genes were found to have 26 in European and 7 in Japanese eels. Since eels can briefly live on land, they may have retained the group γ genes. The number of group β genes which detect airborne and water-soluble odor molecules, were low in the freshwater eels but high in arowana (35) and spotted gar (20). The group eta (η) genes (type 2) is the third major OR gene group in the freshwater eels. The group η genes are mainly expressed in fishes and are absent in mammals (Niimura & Nei 2005).

The voltage-gated Ca^{2+} channels were the significantly expanded gene families in Japanese eels. Genome studies suggest that the cellular functions of voltage-gated ion channels emerged early in Metazoan evolution (Moran & Zakon 2014; Senatore *et al.* 2016) in determining physiology and behavior at the time of early divergence. It is probably associated with the physiological challenge of Japanese eels to maintain a narrow range of intrinsic Ca^{2+} during migration between waters with great variations of calcium contents. A gene expression study in marbled eel (*Anguilla marmorata*) showed the high expression of voltage-gated Ca^{2+}-channels in brain, skin, and osmoregulatory tissues (i.e., gills, intestine, and kidneys) and its response to changes in water calcium levels (Cao *et al.* 2020). Besides controlling Ca^{2+} homeostasis, Ca^{2+}-signaling coordinates various physiological processes, including skeletal muscle contractions, nervous system activity, and cardiac and reproductive functions. The expanded gene families of thick myosin filament in skeletal muscle imply enhanced coordination of muscle contraction and performance (Schneider & Chandler 1973), especially for this distinct clade of elongated bodies inhabiting a diverse range of habitats (Pfaff *et al.* 2016). Additionally, the expanded gene families in lipoprotein receptor-related protein and the isocitrate and isopropyl malate dehydrogenases unravel the importance of these fundamental metabolic and morphogenetic functions in this lineage. Interestingly, lipoprotein receptor-related proteins first appeared during an evolutionary burst associated with the first multicellular organisms, and are multifunctional receptors in nervous system to modulate signals in brains (Herz & Bock 2002; Dieckmann *et al.* 2010). Isocitrate dehydrogenase is an important enzyme of carbohydrate metabolism, while isopropyl malate dehydrogenase is involved in leucine biosynthesis. Although Japanese eels underwent 3R-WGD, an additional TD and PD duplication was detected. These duplication events, genetic raw materials were provided to facilitate new adaptations to the changing environment (Moriyama & Koshiba-Takeuchi 2018). The duplicated genes might have strengthened immune-related responses against different pathogens, contributing to the decline of eel populations (Danne *et al.* 2022; Bandin *et al.* 2014; Kennedy 2007). Presumably, physiological fitness for adaptation might have been weakened by changes in the ecological environment, causing these evolutionary novelties (Belyayev 2014). Notably, the positive selection of immune-related genes

indicates the adaptive advantages of the additional TD and PD duplication. Intriguingly, duplicated immune genes were also observed in salmon (Kjaerner-Semb *et al.* 2016) and sturgeon (Du *et al.* 2020).

The acquisition of evolutionary novelty by WGD duplication and the subsequent fate change of duplicated genes is necessary for phenotype alteration, environmental adaptation, and speciation (Moriyama & Koshiba-Takeuchi 2018). The large-scale genomic reshaping after the third round of WGD affects evolutionary complexity and novelty in teleost fishes (Inoue *et al.* 2015; Glasauer & Neuhauss 2014). It has been widely established that chromosomal numbers are the most fundamental genomic characteristic of an organism or a lineage (Mayrose & Lysak 2021). Based on the hypothesis that genome duplication results in chromosomal rearrangements (Jaillon *et al.* 2004), understanding the rearrangement event in the eel genome may provide insight into the evolution of karyotype numbers at the base of the teleost evolutionary tree. The majority of fishes today have between 40 and 60 chromosomes (diploid number), while some commonly ancestral fishes are thought to have 48 chromosomes. Chromosome rearrangement and duplication have been the principal mechanisms involved in fish evolution, including the generation of new species and development of sex chromosomes. It is noted that freshwater fishes generally have higher number of chromosomes (the modal diploid number = 54) than marine fishes (the modal diploid number = 48). It has been suggested that the higher number of chromosomes in freshwater fishes is related to a less stable freshwater environment with greater topographical barriers (Nikolsky 1976). On the other hand, a large capacity for dispersal in marine environments would contribute to the homogenization of populations, reducing karyotype diversity (Artoni *et al.* 2015). Retrospectively, freshwater species seem to speciate more frequently than marine ones (Bloom *et al.* 2013). Interestingly, Japanese eels, although mostly freshwater dwellers have a marine origin based on phylogenetic analysis of mitogenome sequences (Inoue *et al.* 2010). In a study of reconstructing the vertebrate ancestral genome to reveal dynamic genome reorganization, the 3R-WGD in the teleosts ancestor resulted in the number of chromosomes reaching haploid number (n) 26 (Nakatani *et al.* 2007). Evolutionarily, chromosome numbers peak at n=24 or 25 in extant

teleost species. In this study, we reconstructed the ancestral proto-chromosomes AETK (n = 25) to describe the cross-species chromosome collinearity and underpin the lineage-specific genome reorganization. The chromosome number of *Anguilla species* (n=19) was reduced as compared with *Megalops cyprinoides* (n=25) and *Scleropages formosus* (n=25). The Anguilliformes is made up of 15 families with remarkable karyotypic diversity (Vasconcelos & Molina 2009). The haploid number ranges from 18 to 25, with a prevalence of n = 19 and 21. The *Anguilla* lineage underwent a significant structural rearrangement upon their divergence from the common ancestor of tarpons (*Megalops cyprinoides*). The fusion and fission of their chromosome structure were the primary drivers of reducing the haploid chromosome number to 19.

ACKNOWLEDGEMENTS

This work was supported by the Southern Marine Science and Engineering Guangdong Laboratory (Guangzhou) (SMSEGL20SC02) to CKCW, AOLW, TFC & KPL; General Research Fund (Research Grant Council, HKBU12162016) to CKCW and the Collaborative Research Fund - Earth BioGenome Project Hong Kong (Research Grant Council, C4015-20E) to TFC and CKCW.

AUTHOR CONTRIBUTIONS

The experimental plan and sequencing strategy were designed by Chris KC Wong, Eric L Zhang, Anderson OL Wong, Keng P Lai, and Ting Fung Chan. Samples were collected by Alice HM Ng and Hing Ting Wan. Bionano optical mapping and data analysis were conducted by Claire YL Chung, Eugene YCg Chow, and Jizhou Zhang. The sequencing data for genome assembly was analyzed by Eric L Zhang, Hongbo Wang, Bin Wu, Jianbo Jian, Eugene YC Chow, and Ting Fung Chan. The manuscript was written by Chris KC Wong, HT Wan, Eric L Zhang, Hongbo Wang, and Anderson OL Wong.

DATA AVAILABILITY STATEMENT

The Anguilla japonica whole genome sequencing and assembly are publicly available on NCBI databases under the accession number PRJNA852364. The gene models are available at https://zenodo.org/record/7099450.

ADDITIONAL FILES

Supplementary Fig S1. The alignment plot shows our mitochondrial genomes assembled and GenBank ID AB038556.2 of Japanese eels.

Supplementary Fig S2. Circos plot for the mitochondrial genome of Japanese eel. From outer to inner circles: protein-coding genes, rRNA, and tRNA; depth of Illumina short-reads; GC content.

Supplementary Fig S3. Multi-platform Japanese eel genome assembly.

Supplementary Fig S4. Genome comparison of Japanese (*A. japonica*) and European (*A. anguilla*) eels.

Supplementary Fig S5. Venn diagram of gene annotation based on five databases (NR, InterPro, KEGG, SwissProt, and KOG).

Supplementary Fig S6. KEGG-based gene function classification. The numbers represent how many genes are in the particular functions.

Supplementary Fig S7. KOG-based gene function classification. The numbers represent how many genes are in the particular functions.

Supplementary Fig S8. Length distribution of mRNA, CDS, exon, intron, and the number of exon in Japanese eel and the other species (*A. anguilla, A. rostrata, L. oculatus, M. cyprinoides*).

Supplementary Fig S9. Ks distributions of syntenic paralogs and orthologs. Ks value distribution is used to identify genome duplication and speciation.

Supplementary Fig S10. Comparative genomic analysis of the Japanese eel (*A. japonica*), tarpons (*Megalops cyprinoides*), and arowanas (*Scleropages formosus*).

Supplementary Table S1. Genome sequencing platforms for *Anguilla japonica*.

Supplementary Table S2. A summary of contig statistics from the ONT long read assembly.

Supplementary Table S3. A summary of contig statistics after assembly error correction.

Supplementary Table S4. Scaffolding by 10x linked-reads, Bionano optical mapping, and Hi-C.

Supplementary Table S5. The completeness of *Anguilla japonica* genome by BUSCO assessment.

Supplementary Table S6. Statistical results for repeat sequences.

Supplementary Table S7. A statistical analysis of the classification results for TE.

Supplementary Table S8. Functional annotation of predicted genes from *Anguilla japonica.*

Supplementary Table S9. The completeness of *Anguilla japonica* genes by BUSCO assessment.

Supplementary Table S10. The average length of exons in Japanese eel and the eight related fish species.

Supplementary Table S11. GO enrichment analysis of the gene families expanded in the three freshwater eel genomes.

Supplementary Table S12. GO enrichment analysis of the gene families expanded in the Anguilla japonica genome.

Supplementary Table S13. The karyotypes of *M. cyprinoides* (tarpons) and the common ancestor of eels and tarpons (AETK).

Supplementary Table S14. The karyotypes of *A. japonica* (Japanese eel) and the common ancestor of eels and tarpons (AETK).

FIGURE LEGENDS

Figure 1. The genome landscape of Japanese eel, *Anguilla japonica*. From outer to inner circle: **(A)** length of 19 chromosomes (Mb); **(B)** Read-depth of ONT long-reads; **(C)** Read-depth of PacBio CLR long-reads; **(D)** Read-depth of Illumina short-reads; **(E)** Distribution of transposon sequences; **(F)** Distribution of protein-coding gene; **(G)** GC content; **(H)** Collinear blocks of at least 10 genes in the genome. The window size is 1MB.

Figure 2. Phylogenetic relationship, divergence times and gene families of Anguilla species, relevant bony and cartilaginous fishes. The gene families' expansions (numbers in green) and contractions (numbers in purple) are shown at individual lineages. Each node shows the estimated divergence times (blue numbers, millions of years ago, Mya) and the 95% confidence intervals for these dates. Red dots indicate times taken from the TimeTree website (http://www.timetree.org/). The orange star shows the 3R-WGD event. Geological periods from left to right: S= Silurian, D= Devonian, C= Carboniferous, P= Permian, T= Triassic, J= Jurassic, K= Cretaceous, Pa= Paleogene, N= Neogene. A comparison of gene families associated with orthologs and paralogs in Japanese eel and the 11 fish species.

Figure 3. Number and classification of olfactory receptor (OR) genes for 10 fish species. On the left is the phylogenetic tree of the 10 species. The number of OR genes is shown on the right. The size of the circle indicates the number of OR genes.

Figure 4. (A) Four-fold synonymous third-codon transversion rate (4dTv) distributions of homologous gene pairs for intra-species (paralogs density) and inter-species (orthologs density) comparisons. **(B)** The collinear relationships of syntenic blocks among *Anguilla japonica, Danio rerio,* and *Lepisosteus oculatus*. The numbers indicate the corresponding chromosomes for each species. In *Lepisosteus oculatus*, the 29th chromosome is 293.7 Kb long, which has no collinearity with that of *Anguilla japonica*. Based on homologous blocks of at least 10 genes, gene links between these two species were identified. The four collinear blocks that contain Hox genes are shown in green, yellow, red, and blue. **(C)** Ks distributions of syntenic gene pairs from different gene duplications (wgd: whole genome duplication, trd: transposable duplication, td: tandem duplication, pd: proximal duplication, dsd: dispersed duplication). The y-axis shows the distribution of Ks values. **(D)** Enrichment analysis of five duplicated expansion gene families, with the circles' color representing the GO's statistical significance. The circle size represents the number of genes.

Figure 5. Reconstruction of proto-chromosomes for the common ancestor of teleosts (ATK) and eel/tarpons (AETK). (A) A model for the distribution of chromosomal segments in the genomes of ATK, arowana, AETK, Japanese eels, and tarpons. AETK is the common ancestor of tarpons and eels. The Circos plots indicate conservation of synteny between **(B)** Japanese eel and tarpon, as well as **(C)** arowana and Japanese eel.

Reference List

Altschul SF, Gish W, Miller W, Myers EW & Lipman DJ 1990 Basic local alignment search tool. *J Mol.Biol* **215** 403-410.

Amores A, Force A, Yan YL, Joly L, Amemiya C, Fritz A, Ho RK, Langeland J, Prince V, Wang YL, Westerfield M, Ekker M & Postlethwait JH 1998 Zebrafish hox clusters and vertebrate genome evolution. *Science* **282** 1711-1714.

Artoni RF, Castro JP, Jacobina UP, Lima-Filho PA, da Costa GW & Molina WF 2015 Inferring Diversity and Evolution in Fish by Means of Integrative Molecular Cytogenetics. *ScientificWorldJournal.* **2015** 365787.

Ashburner M, Ball CA, Blake JA, Botstein D, Butler H, Cherry JM, Davis AP, Dolinski K, Dwight SS, Eppig JT, Harris MA, Hill DP, Issel-Tarver L, Kasarskis A, Lewis S, Matese JC, Richardson JE, Ringwald M, Rubin GM & Sherlock G 2000 Gene ontology: tool for the unification of biology. The Gene Ontology Consortium. *Nat.Genet.* **25** 25-29.

Bandin I, Souto S, Cutrin JM, Lopez-Vazquez C, Olveira JG, Esteve C, Alcaide E & Dopazo CP 2014 Presence of viruses in wild eels Anguilla anguilla L, from the Albufera Lake (Spain). *J Fish Dis.* **37** 597-607.

Belyayev A 2014 Bursts of transposable elements as an evolutionary driving force. *J Evol.Biol* **27** 2573-2584.

Benson G 1999 Tandem repeats finder: a program to analyze DNA sequences. *Nucleic Acids Res.* **27** 573-580.

Bi X, Wang K, Yang L, Pan H, Jiang H, Wei Q, Fang M, Yu H, Zhu C, Cai Y, He Y, Gan X, Zeng H, Yu D, Zhu Y, Jiang H, Qiu Q, Yang H, Zhang YE, Wang W, Zhu M, He S & Zhang G 2021 Tracing the genetic footprints of vertebrate landing in non-teleost ray-finned fishes. *Cell* **184** 1377-1391.

Bian C, Hu Y, Ravi V, Kuznetsova IS, Shen X, Mu X, Sun Y, You X, Li J, Li X, Qiu Y, Tay BH, Thevasagayam NM, Komissarov AS, Trifonov V, Kabilov M, Tupikin A, Luo J, Liu Y, Song H, Liu C, Wang X, Gu D, Yang Y, Li W, Polgar G, Fan G, Zeng P, Zhang H, Xiong Z, Tang Z, Peng C, Ruan Z, Yu H, Chen J, Fan M, Huang Y, Wang M, Zhao X, Hu G, Yang H, Wang J, Wang J, Xu X, Song L, Xu G, Xu P, Xu J, O'Brien SJ, Orban L, Venkatesh B & Shi Q 2016 The Asian arowana (Scleropages formosus) genome provides new insights into the evolution of an early lineage of teleosts. *Sci.Rep* **6** 24501.

Bloom DD, Weir JT, Piller KR & Lovejoy NR 2013 Do freshwater fishes diversify faster than marine fishes? A test using state-dependent diversification analyses and molecular phylogenetics of new world silversides (atherinopsidae). *Evolution* **67** 2040-2057.

Boeckmann B, Bairoch A, Apweiler R, Blatter MC, Estreicher A, Gasteiger E, Martin MJ, Michoud K, O'Donovan C, Phan I, Pilbout S & Schneider M 2003 The SWISS-PROT protein knowledgebase and its supplement TrEMBL in 2003. *Nucleic Acids Res.* **31** 365-370.

Braasch I, Gehrke AR, Smith JJ, Kawasaki K, Manousaki T, Pasquier J, Amores A, Desvignes T, Batzel P, Catchen J, Berlin AM, Campbell MS, Barrell D, Martin KJ, Mulley JF, Ravi V, Lee AP, Nakamura T, Chalopin D, Fan S, Wcisel D, Canestro C, Sydes J, Beaudry FE, Sun Y, Hertel J, Beam MJ, Fasold M, Ishiyama M, Johnson J, Kehr S, Lara M, Letaw JH, Litman GW, Litman RT, Mikami M, Ota T, Saha NR, Williams L, Stadler PF, Wang H, Taylor JS, Fontenot Q, Ferrara A, Searle SM, Aken B, Yandell M, Schneider I, Yoder JA, Volff JN, Meyer A, Amemiya CT, Venkatesh B, Holland PW, Guiguen Y, Bobe J, Shubin NH, Di PF, Alfoldi J, Lindblad-Toh K & Postlethwait JH 2016 The spotted gar genome illuminates vertebrate evolution and facilitates human-teleost comparisons. *Nat.Genet.* **48** 427-437.

Cao Q, Chu P, Gu J, Zhang H, Feng R, Wen X, Wang D, Xiong W, Wang T & Yin S 2020 The influence of Ca(2+) concentration on voltage-dependent L-type calcium channels' expression in the marbled eel (Anguilla marmorata). *Gene* **722** 144101.

Chakraborty M, Baldwin-Brown JG, Long AD & Emerson JJ 2016 Contiguous and accurate de novo assembly of metazoan genomes with modest long read coverage. *Nucleic Acids Res.* **44** e147.

Chang YK, Miyazawa Y, Miller MJ & Tsukamoto K 2018 Potential impact of ocean circulation on the declining Japanese eel catches. *Sci.Rep.* **8** 5496.

Chauve C & Tannier E 2008 A methodological framework for the reconstruction of contiguous regions of ancestral genomes and its application to mammalian genomes. *PLoS.Comput.Biol* **4** e1000234.

Chen S, Zhang G, Shao C, Huang Q, Liu G, Zhang P, Song W, An N, Chalopin D, Volff JN, Hong Y, Li Q, Sha Z, Zhou H, Xie M, Yu Q, Liu Y, Xiang H, Wang N, Wu K, Yang C, Zhou Q, Liao X, Yang L, Hu Q, Zhang J, Meng L, Jin L, Tian Y, Lian J, Yang J, Miao G, Liu S, Liang Z, Yan F, Li Y, Sun B, Zhang H, Zhang J, Zhu Y, Du M, Zhao Y, Schartl M, Tang Q & Wang J 2014 Whole-genome sequence of a flatfish provides insights into ZW sex chromosome evolution and adaptation to a benthic lifestyle. *Nat.Genet.* **46** 253-260.

Chen W, Bian C, You X, Li J, Ye L, Wen Z, Lv Y, Zhang X, Xu J, Yang S, Gu R, Lin X & Shi Q 2019 Genome Sequencing of the Japanese Eel (Anguilla japonica) for Comparative Genomic Studies on tbx4 and a tbx4 Gene Cluster in Teleost Fishes. *Mar Drugs* **17**.

Churcher AM, Hubbard PC, Marques JP, Canario AV & Huertas M 2015 Deep sequencing of the olfactory epithelium reveals specific chemosensory receptors are expressed at sexual maturity in the European eel Anguilla anguilla. *Mol.Ecol* **24** 822-834.

Coombe L, Zhang J, Vandervalk BP, Chu J, Jackman SD, Birol I & Warren RL 2018 ARKS: chromosome-scale scaffolding of human genome drafts with linked read kmers. *BMC.Bioinformatics.* **19** 234.

Danne L, Horn L, Feldhaus A, Fey D, Emde S, Schutze H, Adamek M & Hellmann J 2022 Virus infections of the European Eel in North Rhine Westphalian rivers. *J Fish Dis.* **45** 69-76.

De BT, Cristianini N, Demuth JP & Hahn MW 2006 CAFE: a computational tool for the study of gene family evolution. *Bioinformatics.* **22** 1269-1271.

Dieckmann M, Dietrich MF & Herz J 2010 Lipoprotein receptors--an evolutionarily ancient multifunctional receptor family. *Biol Chem.* **391** 1341-1363.

Du K, Stock M, Kneitz S, Klopp C, Woltering JM, Adolfi MC, Feron R, Prokopov D, Makunin A, Kichigin I, Schmidt C, Fischer P, Kuhl H, Wuertz S, Gessner J, Kloas W, Cabau C, Iampietro C, Parrinello H, Tomlinson C, Journot L, Postlethwait JH, Braasch I, Trifonov V, Warren WC, Meyer A, Guiguen Y & Schartl M 2020 The sterlet sturgeon genome sequence and the mechanisms of segmental rediploidization. *Nat.Ecol Evol.* **4** 841-852.

Dudchenko O, Batra SS, Omer AD, Nyquist SK, Hoeger M, Durand NC, Shamim MS, Machol I, Lander ES, Aiden AP & Aiden EL 2017 De novo assembly of the Aedes aegypti genome using Hi-C yields chromosome-length scaffolds. *Science* **356** 92-95.

Durand NC, Robinson JT, Shamim MS, Machol I, Mesirov JP, Lander ES & Aiden EL 2016 Juicebox Provides a Visualization System for Hi-C Contact Maps with Unlimited Zoom. *Cell Syst.* **3** 99-101.

Freitag J, Krieger J, Strotmann J & Breer H 1995 Two classes of olfactory receptors in Xenopus laevis. *Neuron* **15** 1383-1392.

Geeraerts C & Belpaire C 2010 The effects of contaminants in European eel: a review. *Ecotoxicology.* **19** 239-266.

Gertz EM, Yu YK, Agarwala R, Schaffer AA & Altschul SF 2006 Composition-based statistics and translated nucleotide searches: improving the TBLASTN module of BLAST. *BMC.Biol* **4** 41.

Ghurye J & Pop M 2019 Modern technologies and algorithms for scaffolding assembled genomes. *PLoS.Comput.Biol* **15** e1006994.

Glasauer SM & Neuhauss SC 2014 Whole-genome duplication in teleost fishes and its evolutionary consequences. *Mol.Genet.Genomics* **289** 1045-1060.

Glusman G, Bahar A, Sharon D, Pilpel Y, White J & Lancet D 2000 The olfactory receptor gene superfamily: data mining, classification, and nomenclature. *Mamm.Genome* **11** 1016-1023.

Griffiths-Jones S, Moxon S, Marshall M, Khanna A, Eddy SR & Bateman A 2005 Rfam: annotating non-coding RNAs in complete genomes. *Nucleic Acids Res.* **33** D121-D124.

Gross MR, Coleman RM & McDowall RM 1988 Aquatic productivity and the evolution of diadromous fish migration. *Science* **239** 1291-1293.

Guindon S & Gascuel O 2003 A simple, fast, and accurate algorithm to estimate large phylogenies by maximum likelihood. *Syst.Biol* **52** 696-704.

Haas BJ, Papanicolaou A, Yassour M, Grabherr M, Blood PD, Bowden J, Couger MB, Eccles D, Li B, Lieber M, Macmanes MD, Ott M, Orvis J, Pochet N, Strozzi F, Weeks N, Westerman R, William T, Dewey CN, Henschel R, Leduc RD, Friedman N & Regev A 2013 De novo transcript sequence reconstruction from RNA-seq using the Trinity platform for reference generation and analysis. *Nat.Protoc.* **8** 1494-1512.

Hein JL, Arnott SA, Roumillat WA, Allen DM & de B, I 2014 Invasive swimbladder parasite Anguillicoloides crassus: infection status 15 years after discovery in wild populations of American eel Anguilla rostrata. *Dis.Aquat.Organ* **107** 199-209.

Henkel CV, Burgerhout E, de Wijze DL, Dirks RP, Minegishi Y, Jansen HJ, Spaink HP, Dufour S, Weltzien FA, Tsukamoto K & van den Thillart GE 2012a Primitive duplicate Hox clusters in the European eel's genome. *PLoS.ONE.* **7** e32231.

Henkel CV, Dirks RP, de Wijze DL, Minegishi Y, Aoyama J, Jansen HJ, Turner B, Knudsen B, Bundgaard M, Hvam KL, Boetzer M, Pirovano W, Weltzien FA, Dufour S, Tsukamoto K, Spaink HP & van den Thillart GE 2012b First draft genome sequence of the Japanese eel, Anguilla japonica. *Gene* **511** 195-201.

Herz J & Bock HH 2002 Lipoprotein receptors in the nervous system. *Annu Rev Biochem.* **71** 405-434.

Holt C & Yandell M 2011 MAKER2: an annotation pipeline and genome-database management tool for second-generation genome projects. *BMC.Bioinformatics.* **12** 491.

Howe K, Clark MD, Torroja CF, Torrance J, Berthelot C, Muffato M, Collins JE, Humphray S, McLaren K, Matthews L, McLaren S, Sealy I, Caccamo M, Churcher C, Scott C, Barrett JC, Koch R, Rauch GJ, White S, Chow W, Kilian B, Quintais LT, Guerra-Assuncao JA, Zhou Y, Gu Y, Yen J, Vogel JH, Eyre T, Redmond S, Banerjee R, Chi J, Fu B, Langley E, Maguire SF, Laird GK, Lloyd D, Kenyon E, Donaldson S, Sehra H,

Almeida-King J, Loveland J, Trevanion S, Jones M, Quail M, Willey D, Hunt A, Burton J, Sims S, McLay K, Plumb B, Davis J, Clee C, Oliver K, Clark R, Riddle C, Elliot D, Threadgold G, Harden G, Ware D, Begum S, Mortimore B, Kerry G, Heath P, Phillimore B, Tracey A, Corby N, Dunn M, Johnson C, Wood J, Clark S, Pelan S, Griffiths G, Smith M, Glithero R, Howden P, Barker N, Lloyd C, Stevens C, Harley J, Holt K, Panagiotidis G, Lovell J, Beasley H, Henderson C, Gordon D, Auger K, Wright D, Collins J, Raisen C, Dyer L, Leung K, Robertson L, Ambridge K, Leongamornlert D, McGuire S, Gilderthorp R, Griffiths C, Manthravadi D, Nichol S, Barker G, Whitehead S, Kay M, Brown J, Murnane C, Gray E, Humphries M, Sycamore N, Barker D, Saunders D, Wallis J, Babbage A, Hammond S, Mashreghi-Mohammadi M, Barr L, Martin S, Wray P, Ellington A, Matthews N, Ellwood M, Woodmansey R, Clark G, Cooper J, Tromans A, Grafham D, Skuce C, Pandian R, Andrews R, Harrison E, Kimberley A, Garnett J, Fosker N, Hall R, Garner P, Kelly D, Bird C, Palmer S, Gehring I, Berger A, Dooley CM, Ersan-Urun Z, Eser C, Geiger H, Geisler M, Karotki L, Kirn A, Konantz J, Konantz M, Oberlander M, Rudolph-Geiger S, Teucke M, Lanz C, Raddatz G, Osoegawa K, Zhu B, Rapp A, Widaa S, Langford C, Yang F, Schuster SC, Carter NP, Harrow J, Ning Z, Herrero J, Searle SM, Enright A, Geisler R, Plasterk RH, Lee C, Westerfield M, de Jong PJ, Zon LI, Postlethwait JH, Nusslein-Volhard C, Hubbard TJ, Roest CH, Rogers J & Stemple DL 2013 The zebrafish reference genome sequence and its relationship to the human genome. *Nature* **496** 498-503.

Hughes LC, Orti G, Huang Y, Sun Y, Baldwin CC, Thompson AW, Arcila D, Betancur R, Li C, Becker L, Bellora N, Zhao X, Li X, Wang M, Fang C, Xie B, Zhou Z, Huang H, Chen S, Venkatesh B & Shi Q 2018 Comprehensive phylogeny of ray-finned fishes (Actinopterygii) based on transcriptomic and genomic data. *Proc.Natl.Acad.Sci.U.S.A* **115** 6249-6254.

Hurley IA, Mueller RL, Dunn KA, Schmidt EJ, Friedman M, Ho RK, Prince VE, Yang Z, Thomas MG & Coates MI 2007 A new time-scale for ray-finned fish evolution. *Proc.Biol Sci.* **274** 489-498.

Inoue J, Sato Y, Sinclair R, Tsukamoto K & Nishida M 2015 Rapid genome reshaping by multiple-gene loss after whole-genome duplication in teleost fish suggested by mathematical modeling. *Proc.Natl.Acad.Sci.U.S.A* **112** 14918-14923.

Inoue JG, Miya M, Miller MJ, Sado T, Hanel R, Hatooka K, Aoyama J, Minegishi Y, Nishida M & Tsukamoto K 2010 Deep-ocean origin of the freshwater eels. *Biol Lett.* **6** 363-366.

Jackman SD, Coombe L, Chu J, Warren RL, Vandervalk BP, Yeo S, Xue Z, Mohamadi H, Bohlmann J, Jones SJM & Birol I 2018 Tigmint: correcting assembly errors using linked reads from large molecules. *BMC.Bioinformatics.* **19** 393.

Jaillon O, Aury JM, Brunet F, Petit JL, Stange-Thomann N, Mauceli E, Bouneau L, Fischer C, Ozouf-Costaz C, Bernot A, Nicaud S, Jaffe D, Fisher S, Lutfalla G, Dossat C, Segurens

B, Dasilva C, Salanoubat M, Levy M, Boudet N, Castellano S, Anthouard V, Jubin C, Castelli V, Katinka M, Vacherie B, Biemont C, Skalli Z, Cattolico L, Poulain J, De B, V, Cruaud C, Duprat S, Brottier P, Coutanceau JP, Gouzy J, Parra G, Lardier G, Chapple C, McKernan KJ, McEwan P, Bosak S, Kellis M, Volff JN, Guigo R, Zody MC, Mesirov J, Lindblad-Toh K, Birren B, Nusbaum C, Kahn D, Robinson-Rechavi M, Laudet V, Schachter V, Quetier F, Saurin W, Scarpelli C, Wincker P, Lander ES, Weissenbach J & Roest CH 2004 Genome duplication in the teleost fish Tetraodon nigroviridis reveals the early vertebrate proto-karyotype. *Nature* **431** 946-957.

Jansen HJ, Liem M, Jong-Raadsen SA, Dufour S, Weltzien FA, Swinkels W, Koelewijn A, Palstra AP, Pelster B, Spaink HP, Thillart GEVD, Dirks RP & Henkel CV 2017 Rapid de novo assembly of the European eel genome from nanopore sequencing reads. *Sci.Rep* **7** 7213.

Jehannet P, Palstra AP, Heinsbroek LTN, Kruijt L, Dirks RP, Swinkels W & Komen H 2021 What Goes Wrong during Early Development of Artificially Reproduced European Eel Anguilla anguilla? Clues from the Larval Transcriptome and Gene Expression Patterns. *Animals.(Basel)* **11**.

Jensen MR, Knudsen SW, Munk P, Thomsen PF & Moller PR 2018 Tracing European eel in the diet of mesopelagic fishes from the Sargasso Sea using DNA from fish stomachs. *Marine Biology* **165** 130.

Johnson AD, Handsaker RE, Pulit SL, Nizzari MM, O'Donnell CJ & de Bakker PI 2008 SNAP: a web-based tool for identification and annotation of proxy SNPs using HapMap. *Bioinformatics.* **24** 2938-2939.

Jurka J, Kapitonov VV, Pavlicek A, Klonowski P, Kohany O & Walichiewicz J 2005 Repbase Update, a database of eukaryotic repetitive elements. *Cytogenet.Genome Res.* **110** 462-467.

Kai W, Nomura K, Fujiwara A, Nakamura Y, Yasuike M, Ojima N, Masaoka T, Ozaki A, Kazeto Y, Gen K, Nagao J, Tanaka H, Kobayashi T & Ototake M 2014 A ddRAD-based genetic map and its integration with the genome assembly of Japanese eel (Anguilla japonica) provides insights into genome evolution after the teleost-specific genome duplication. *BMC.Genomics* **15** 233.

Kanehisa M & Goto S 2000 KEGG: kyoto encyclopedia of genes and genomes. *Nucleic Acids Res.* **28** 27-30.

Katoh K & Standley DM 2013 MAFFT multiple sequence alignment software version 7: improvements in performance and usability. *Mol.Biol Evol.* **30** 772-780.

Kennedy CR 2007 The pathogenic helminth parasites of eels. *J Fish Dis.* **30** 319-334.

Kim D, Langmead B & Salzberg SL 2015 HISAT: a fast spliced aligner with low memory requirements. *Nat.Methods* **12** 357-360.

Kjaerner-Semb E, Ayllon F, Furmanek T, Wennevik V, Dahle G, Niemela E, Ozerov M, Vaha JP, Glover KA, Rubin CJ, Wargelius A & Edvardsen RB 2016 Atlantic salmon populations reveal adaptive divergence of immune related genes - a duplicated genome under selection. *BMC.Genomics* **17** 610.

Kolmogorov M, Yuan J, Lin Y & Pevzner PA 2019 Assembly of long, error-prone reads using repeat graphs. *Nat.Biotechnol.* **37** 540-546.

Koren S, Walenz BP, Berlin K, Miller JR, Bergman NH & Phillippy AM 2017 Canu: scalable and accurate long-read assembly via adaptive k-mer weighting and repeat separation. *Genome Res.* **27** 722-736.

Li L, Stoeckert CJ, Jr. & Roos DS 2003 OrthoMCL: identification of ortholog groups for eukaryotic genomes. *Genome Res.* **13** 2178-2189.

Lien S, Koop BF, Sandve SR, Miller JR, Kent MP, Nome T, Hvidsten TR, Leong JS, Minkley DR, Zimin A, Grammes F, Grove H, Gjuvsland A, Walenz B, Hermansen RA, von SK, Rondeau EB, Di GA, Samy JK, Olav VJ, Vigeland MD, Caler L, Grimholt U, Jentoft S, Vage DI, de JP, Moen T, Baranski M, Palti Y, Smith DR, Yorke JA, Nederbragt AJ, Tooming-Klunderud A, Jakobsen KS, Jiang X, Fan D, Hu Y, Liberles DA, Vidal R, Iturra P, Jones SJ, Jonassen I, Maass A, Omholt SW & Davidson WS 2016 The Atlantic salmon genome provides insights into rediploidization. *Nature* **533** 200-205.

Liu YC, Hsu SD, Chou CH, Huang WY, Chen YH, Liu CY, Lyu GJ, Huang SZ, Aganezov S, Alekseyev MA, Hsiao CD & Huang HD 2016 Transcriptome sequencing based annotation and homologous evidence based scaffolding of Anguilla japonica draft genome. *BMC.Genomics* **17 Suppl 1** 13.

Lowe TM & Eddy SR 1997 tRNAscan-SE: a program for improved detection of transfer RNA genes in genomic sequence. *Nucleic Acids Res.* **25** 955-964.

Mayrose I & Lysak MA 2021 The Evolution of Chromosome Numbers: Mechanistic Models and Experimental Approaches. *Genome Biol Evol.* **13**.

Meng G, Li Y, Yang C & Liu S 2019 MitoZ: a toolkit for animal mitochondrial genome assembly, annotation and visualization. *Nucleic Acids Res.* **47** e63.

Moran Y & Zakon HH 2014 The evolution of the four subunits of voltage-gated calcium channels: ancient roots, increasing complexity, and multiple losses. *Genome Biol Evol.* **6** 2210-2217.

Moriyama Y & Koshiba-Takeuchi K 2018 Significance of whole-genome duplications on the emergence of evolutionary novelties. *Brief.Funct.Genomics* **17** 329-338.

Nakamura Y, Yasuike M, Mekuchi M, Iwasaki Y, Ojima N, Fujiwara A, Chow S & Saitoh K 2017 Rhodopsin gene copies in Japanese eel originated in a teleost-specific genome duplication. *Zoological.Lett.* **3** 18.

Nakatani Y, Takeda H, Kohara Y & Morishita S 2007 Reconstruction of the vertebrate ancestral genome reveals dynamic genome reorganization in early vertebrates. *Genome Res.* **17** 1254-1265.

Near TJ, Eytan RI, Dornburg A, Kuhn KL, Moore JA, Davis MP, Wainwright PC, Friedman M & Smith WL 2012 Resolution of ray-finned fish phylogeny and timing of diversification. *Proc.Natl.Acad.Sci.U.S.A* **109** 13698-13703.

Nguyen LT, Schmidt HA, von HA & Minh BQ 2015 IQ-TREE: a fast and effective stochastic algorithm for estimating maximum-likelihood phylogenies. *Mol.Biol Evol.* **32** 268-274.

Niimura Y 2009 On the origin and evolution of vertebrate olfactory receptor genes: comparative genome analysis among 23 chordate species. *Genome Biol Evol.* **1** 34-44.

Niimura Y & Nei M 2005 Evolutionary dynamics of olfactory receptor genes in fishes and tetrapods. *Proc.Natl.Acad.Sci.U.S.A* **102** 6039-6044.

Nikolsky G 1976 THE INTERRELATION BETWEEN VARIABILITY OF CHARACTERS, EFFECTIVENESS OF ENERGY UTILISATION, AND KARYOTYPE STRUCTURE IN FISHES. *Evolution* **30** 180-185.

Pan W, Jiang T & Lonardi S 2020 OMGS: Optical Map-Based Genome Scaffolding. *J Comput.Biol* **27** 519-533.

Parey E, Louis A, Montfort J, Bouchez O, Roques C, Iampietro C, Lluch J, Castinel A, Donnadieu C, Desvignes T, Bucao CF, Jouanno E, Wen M, Mejri S, Dirks R, Jansen H, Henkel C, Chen W, Zahm M, Cabau C, Klopp C, Thompson AW, Robinson-Rechavi M, Braasch I, Lecointre G, Bobe J, Postlethwait JH, Berthelot C, Crollius HR & Guiguen Y 2022 Genome strcutures resolve the early diversification of teleost fishes. *bioRxiv doi:https://doi.org/10.1101/2022.04.07.487469*.

Pavey SA, Laporte M, Normandeau E, Gaudin J, Letourneau L, Boisvert S, Corbeil J, Audet C & Bernatchez L 2017 Draft genome of the American Eel (Anguilla rostrata). *Mol.Ecol.Resour.* **17** 806-811.

Pfaff C, Zorzin R & Kriwet J 2016 Evolution of the locomotory system in eels (Teleostei: Elopomorpha). *BMC.Evol.Biol* **16** 159.

Piper AT, Manes C, Siniscalchi F, Marion A, Wright RM & Kemp PS 2015 Response of seaward-migrating European eel (Anguilla anguilla) to manipulated flow fields. *Proc.Biol.Sci.* **282**.

Policarpo M, Bemis KE, Tyler JC, Metcalfe CJ, Laurenti P, Sandoz JC, Retaux S & Casane D 2021 Evolutionary Dynamics of the OR Gene Repertoire in Teleost Fishes: Evidence of an Association with Changes in Olfactory Epithelium Shape. *Mol.Biol Evol.* **38** 3742-3753.

Qiao X, Li Q, Yin H, Qi K, Li L, Wang R, Zhang S & Paterson AH 2019 Gene duplication and evolution in recurring polyploidization-diploidization cycles in plants. *Genome Biol* **20** 38.

Rhie A, McCarthy SA, Fedrigo O, Damas J, Formenti G, Koren S, Uliano-Silva M, Chow W, Fungtammasan A, Kim J, Lee C, Ko BJ, Chaisson M, Gedman GL, Cantin LJ, Thibaud-Nissen F, Haggerty L, Bista I, Smith M, Haase B, Mountcastle J, Winkler S, Paez S, Howard J, Vernes SC, Lama TM, Grutzner F, Warren WC, Balakrishnan CN, Burt D, George JM, Biegler MT, Iorns D, Digby A, Eason D, Robertson B, Edwards T, Wilkinson M, Turner G, Meyer A, Kautt AF, Franchini P, Detrich HW, III, Svardal H, Wagner M, Naylor GJP, Pippel M, Malinsky M, Mooney M, Simbirsky M, Hannigan BT, Pesout T, Houck M, Misuraca A, Kingan SB, Hall R, Kronenberg Z, Sovic I, Dunn C, Ning Z, Hastie A, Lee J, Selvaraj S, Green RE, Putnam NH, Gut I, Ghurye J, Garrison E, Sims Y, Collins J, Pelan S, Torrance J, Tracey A, Wood J, Dagnew RE, Guan D, London SE, Clayton DF, Mello CV, Friedrich SR, Lovell PV, Osipova E, Al-Ajli FO, Secomandi S, Kim H, Theofanopoulou C, Hiller M, Zhou Y, Harris RS, Makova KD, Medvedev P, Hoffman J, Masterson P, Clark K, Martin F, Howe K, Flicek P, Walenz BP, Kwak W, Clawson H, Diekhans M, Nassar L, Paten B, Kraus RHS, Crawford AJ, Gilbert MTP, Zhang G, Venkatesh B, Murphy RW, Koepfli KP, Shapiro B, Johnson WE, Di PF, Marques-Bonet T, Teeling EC, Warnow T, Graves JM, Ryder OA, Haussler D, O'Brien SJ, Korlach J, Lewin HA, Howe K, Myers EW, Durbin R, Phillippy AM & Jarvis ED 2021 Towards complete and error-free genome assemblies of all vertebrate species. *Nature* **592** 737-746.

Rice P, Longden I & Bleasby A 2000 EMBOSS: the European Molecular Biology Open Software Suite. *Trends Genet.* **16** 276-277.

Rozenfeld C, Blanca J, Gallego V, Garcia-Carpintero V, Herranz-Jusdado JG, Perez L, Asturiano JF, Canizares J & Penaranda DS 2019 De novo European eel transcriptome provides insights into the evolutionary history of duplicated genes in teleost lineages. *PLoS.ONE.* **14** e0218085.

Ruan J & Li H 2020 Fast and accurate long-read assembly with wtdbg2. *Nat.Methods* **17** 155-158.

Schneider MF & Chandler WK 1973 Voltage dependent charge movement of skeletal muscle: a possible step in excitation-contraction coupling. *Nature* **242** 244-246.

Senatore A, Raiss H & Le P 2016 Physiology and Evolution of Voltage-Gated Calcium Channels in Early Diverging Animal Phyla: Cnidaria, Placozoa, Porifera and Ctenophora. *Front Physiol* **7** 481.

Simao FA, Waterhouse RM, Ioannidis P, Kriventseva EV & Zdobnov EM 2015 BUSCO: assessing genome assembly and annotation completeness with single-copy orthologs. *Bioinformatics.* **31** 3210-3212.

Stamatakis A 2014 RAxML version 8: a tool for phylogenetic analysis and post-analysis of large phylogenies. *Bioinformatics.* **30** 1312-1313.

Stanke M, Keller O, Gunduz I, Hayes A, Waack S & Morgenstern B 2006 AUGUSTUS: ab initio prediction of alternative transcripts. *Nucleic Acids Res.* **34** W435-W439.

States DJ & Gish W 1994 Combined use of sequence similarity and codon bias for coding region identification. *J Comput.Biol* **1** 39-50.

Tang H, Bowers JE, Wang X, Ming R, Alam M & Paterson AH 2008 Synteny and collinearity in plant genomes. *Science* **320** 486-488.

Tarailo-Graovac M & Chen N 2009 Using RepeatMasker to identify repetitive elements in genomic sequences. *Curr.Protoc.Bioinformatics.* **Chapter 4** Unit.

Tatusov RL, Fedorova ND, Jackson JD, Jacobs AR, Kiryutin B, Koonin EV, Krylov DM, Mazumder R, Mekhedov SL, Nikolskaya AN, Rao BS, Smirnov S, Sverdlov AV, Vasudevan S, Wolf YI, Yin JJ & Natale DA 2003 The COG database: an updated version includes eukaryotes. *BMC.Bioinformatics.* **4** 41.

Valenzano DR, Benayoun BA, Singh PP, Zhang E, Etter PD, Hu CK, Clement-Ziza M, Willemsen D, Cui R, Harel I, Machado BE, Yee MC, Sharp SC, Bustamante CD, Beyer A, Johnson EA & Brunet A 2015 The African Turquoise Killifish Genome Provides Insights into Evolution and Genetic Architecture of Lifespan. *Cell* **163** 1539-1554.

Vasconcelos AJ & Molina WF 2009 Cytogenetical studies in five Atlantic Anguilliformes fishes. *Genet.Mol.Biol* **32** 83-90.

Vaser R, Sovic I, Nagarajan N & Sikic M 2017 Fast and accurate de novo genome assembly from long uncorrected reads. *Genome Res.* **27** 737-746.

Walker BJ, Abeel T, Shea T, Priest M, Abouelliel A, Sakthikumar S, Cuomo CA, Zeng Q, Wortman J, Young SK & Earl AM 2014 Pilon: an integrated tool for comprehensive microbial variant detection and genome assembly improvement. *PLoS.ONE.* **9** e112963.

Wang D, Zhang Y, Zhang Z, Zhu J & Yu J 2010 KaKs_Calculator 2.0: a toolkit incorporating gamma-series methods and sliding window strategies. *Genomics Proteomics.Bioinformatics.* **8** 77-80.

Wang Y, Tang H, Debarry JD, Tan X, Li J, Wang X, Lee TH, Jin H, Marler B, Guo H, Kissinger JC & Paterson AH 2012 MCScanX: a toolkit for detection and evolutionary analysis of gene synteny and collinearity. *Nucleic Acids Res.* **40** e49.

Xu Z & Wang H 2007 LTR_FINDER: an efficient tool for the prediction of full-length LTR retrotransposons. *Nucleic Acids Res.* **35** W265-W268.

Yang Z 2007 PAML 4: phylogenetic analysis by maximum likelihood. *Mol.Biol Evol.* **24** 1586-1591.

Zdobnov EM & Apweiler R 2001 InterProScan--an integration platform for the signature-recognition methods in InterPro. *Bioinformatics.* **17** 847-848.

Zhang Z, Schwartz S, Wagner L & Miller W 2000 A greedy algorithm for aligning DNA sequences. *J Comput.Biol* **7** 203-214.

 Click here to access/download;Table;Table1.docx

Table 1.

Statistics of *Anguilla japonica* genome assembly and annotation

Assembly feature	*Anguilla japonica*
Genome size, Gb	1.028
No. of contigs	811
Contig N50, Mbp	21.48
Contig N90, Kbp	716.98
Longest contig, Mbp	57.08
No. of scaffolds	86
Scaffold N50, Mbp	58.71
Scaffold N90, Mbp	38.29
Longest scaffold, Mbp	94.29
Repeat portion of assembly, %	30.48
No. of genes	29,982
GC%	44
Genes average length, bp	10265.73
Average exons per gene	9

Click here to access/download;Figure;Figures-updated.pptx

Figure 1

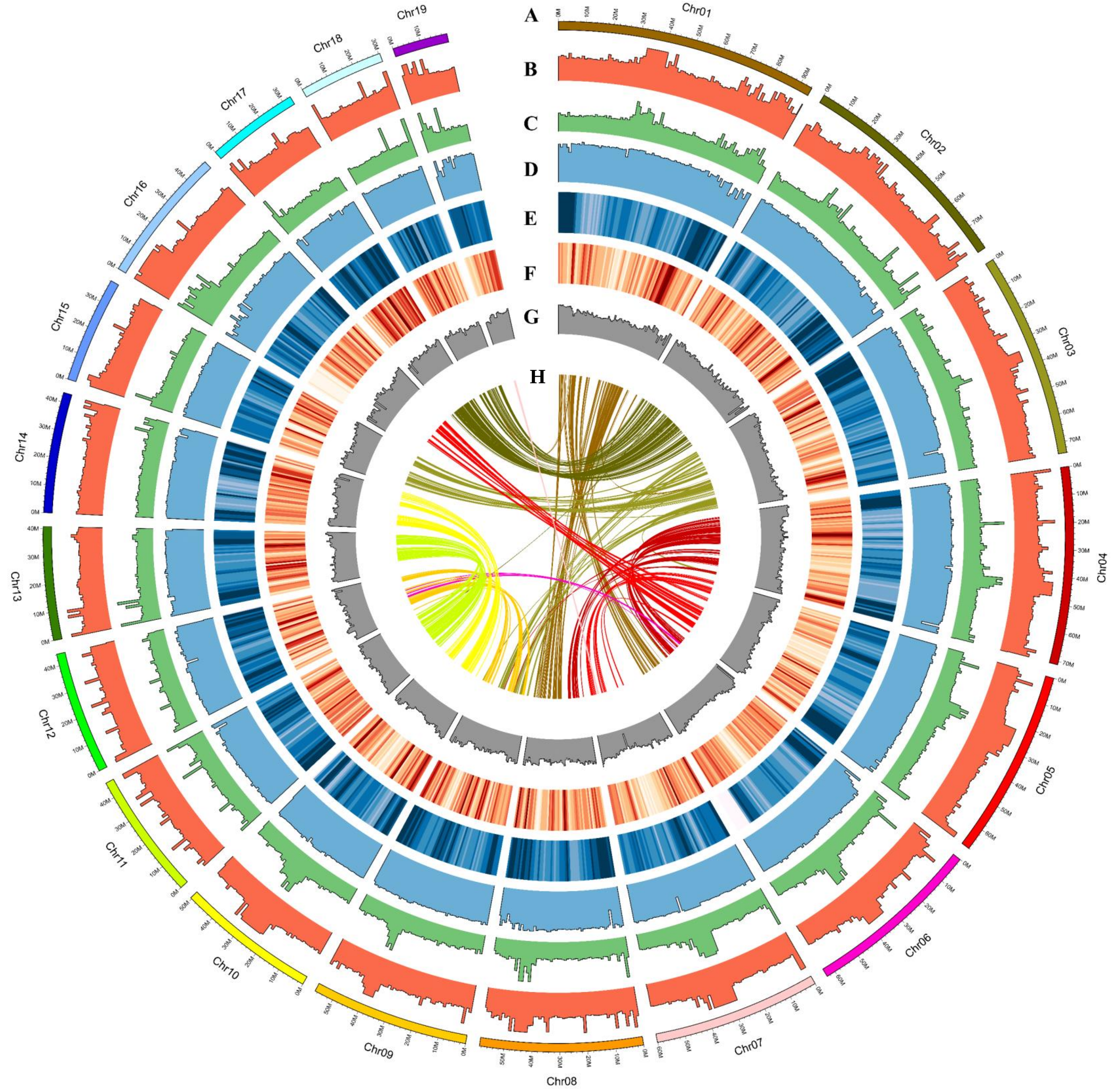

Figure 2

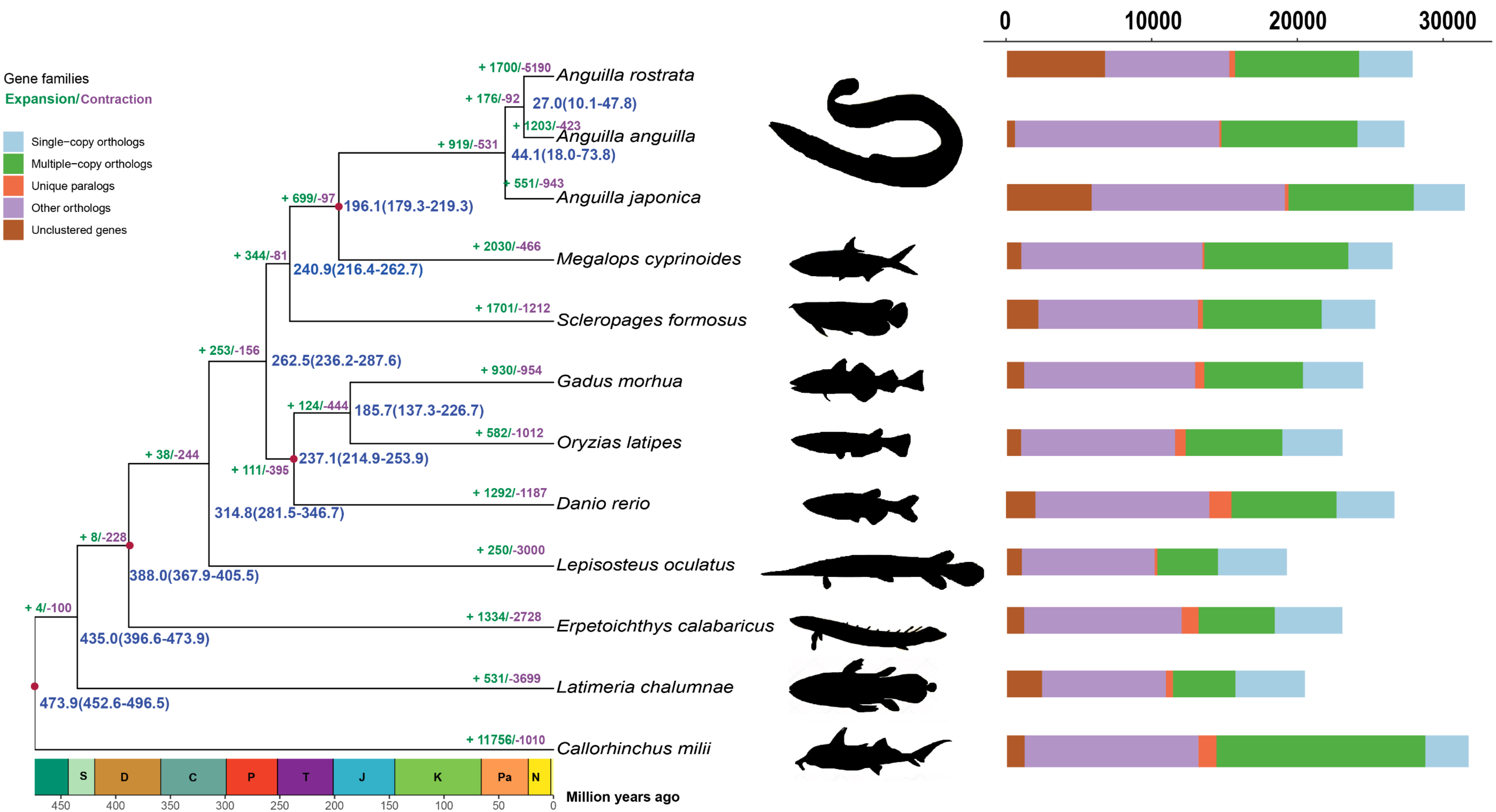

Gene families
Expansion/Contraction
Single−copy orthologs
Multiple−copy orthologs
Unique paralogs
Other orthologs
Unclustered genes
+ 1700/-5190
Anguilla rostrata
+ 176/-92
27.0(10.1-47.8)
+ 1203/-423
Anguilla anguilla
+ 919/-531
44.1(18.0-73.8)
+ 551/-943
Anguilla japonica
+ 699/-97
196.1(179.3-219.3)
+ 2030/-466
Megalops cyprinoides
+ 344/-81
240.9(216.4-262.7)
+ 1701/-1212
Scleropages formosus
+ 253/-156
262.5(236.2-287.6)
+ 930/-954
Gadus morhua
+ 124/-444
185.7(137.3-226.7)
+ 582/-1012
Oryzias latipes
+ 38/-244
237.1(214.9-253.9)
+ 111/-395
+ 1292/-1187
Danio rerio
314.8(281.5-346.7)
+ 8/-228
+ 250/-3000
Lepisosteus oculatus
388.0(367.9-405.5)
+ 4/-100
+ 1334/-2728
Erpetoichthys calabaricus
435.0(396.6-473.9)
+ 531/-3699
Latimeria chalumnae
473.9(452.6-496.5)
+ 11756/-1010
Callorhinchus milii
S D C P T J K Pa N
450 400 350 300 250 200 150 100 50 0
Million years ago
0 10000 20000 30000

Figure 3

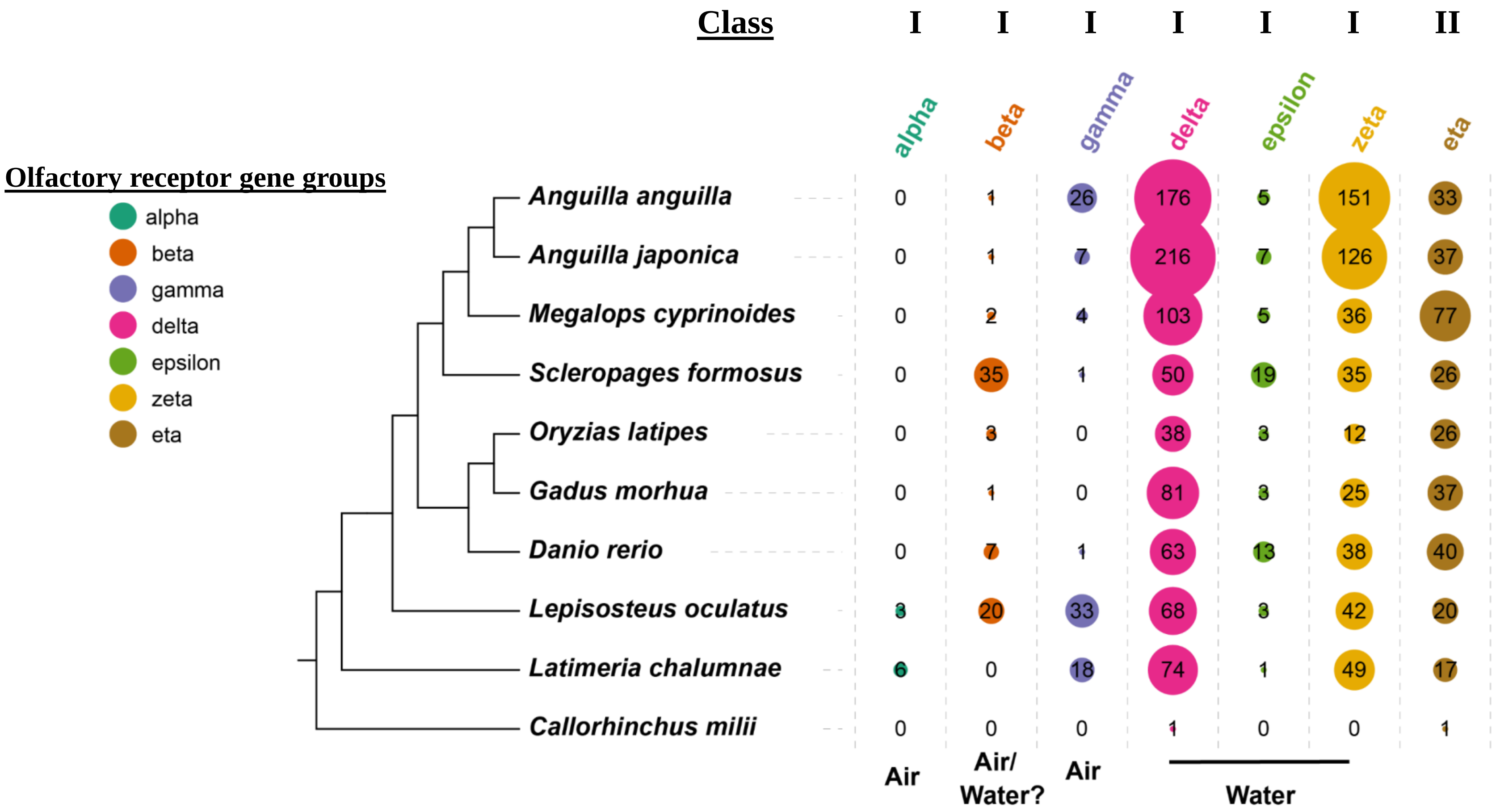

Class
I
I
I
I
I
I
II
alpha
beta
gamma
delta
epsilon
zeta
eta
Olfactory receptor gene groups
alpha
beta
gamma
delta
epsilon
zeta
eta
Anguilla anguilla 0 1 26 176 5 151 33
Anguilla japonica 0 1 7 216 7 126 37
Megalops cyprinoides 0 2 4 103 5 36 77
Scleropages formosus 0 35 1 50 19 35 26
Oryzias latipes 0 3 0 38 3 12 26
Gadus morhua 0 1 0 81 3 25 37
Danio rerio 0 7 1 63 13 38 40
Lepisosteus oculatus 3 20 33 68 3 42 20
Latimeria chalumnae 6 0 18 74 1 49 17
Callorhinchus milii 0 0 0 1 0 0 1
Air
Air/
Water?
Air
Water

Figure 4

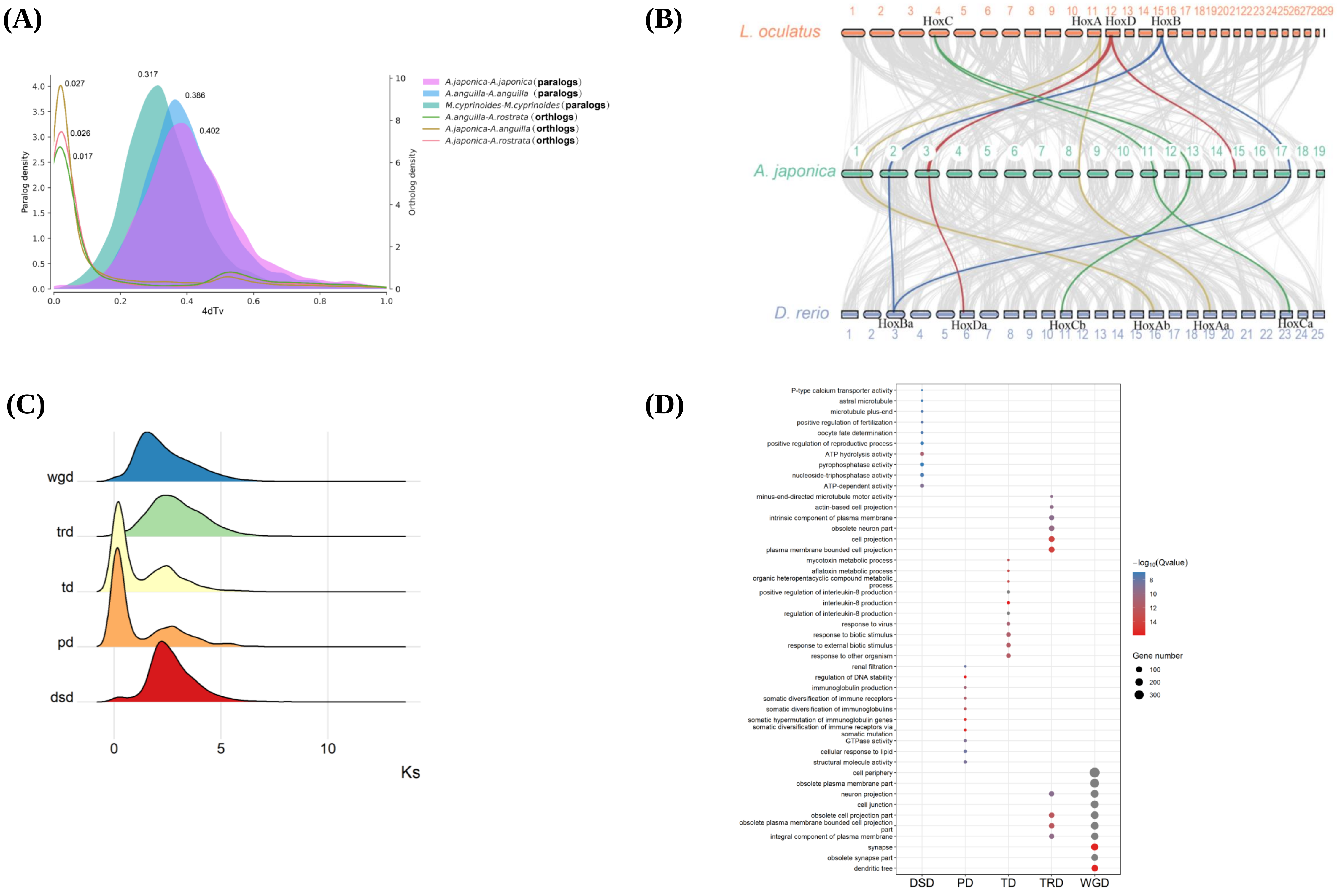
(A)
(B)
(C)
(D)
0.027
0.026
0.017
0.317
0.386
0.402
Paralog density
Ortholog density
4dTv
A.japonica-A.japonica (paralogs)
A.anguilla-A.anguilla (paralogs)
M.cyprinoides-M.cyprinoides (paralogs)
A.anguilla-A.rostrata (orthlogs)
A.japonica-A.anguilla (orthlogs)
A.japonica-A.rostrata (orthlogs)
L. oculatus
A. japonica
D. rerio
HoxC
HoxA
HoxD
HoxB
HoxBa
HoxDa
HoxCb
HoxAb
HoxAa
HoxCa
wgd
trd
td
pd
dsd
Ks
P-type calcium transporter activity
astral microtubule
microtubule plus-end
positive regulation of fertilization
oocyte fate determination
positive regulation of reproductive process
ATP hydrolysis activity
pyrophosphatase activity
nucleoside-triphosphatase activity
ATP-dependent activity
minus-end-directed microtubule motor activity
actin-based cell projection
intrinsic component of plasma membrane
obsolete neuron part
cell projection
plasma membrane bounded cell projection
mycotoxin metabolic process
aflatoxin metabolic process
organic heteropentacyclic compound metabolic process
positive regulation of interleukin-8 production
interleukin-8 production
regulation of interleukin-8 production
response to virus
response to biotic stimulus
response to external biotic stimulus
response to other organism
renal filtration
regulation of DNA stability
immunoglobulin production
somatic diversification of immune receptors
somatic diversification of immunoglobulins
somatic hypermutation of immunoglobulin genes
somatic diversification of immune receptors via somatic mutation
GTPase activity
cellular response to lipid
structural molecule activity
cell periphery
obsolete plasma membrane part
neuron projection
cell junction
obsolete cell projection part
obsolete plasma membrane bounded cell projection part
integral component of plasma membrane
synapse
obsolete synapse part
dendritic tree
DSD
PD
TD
TRD
WGD
−log10(Qvalue)
Gene number
100
200
300

Figure 5

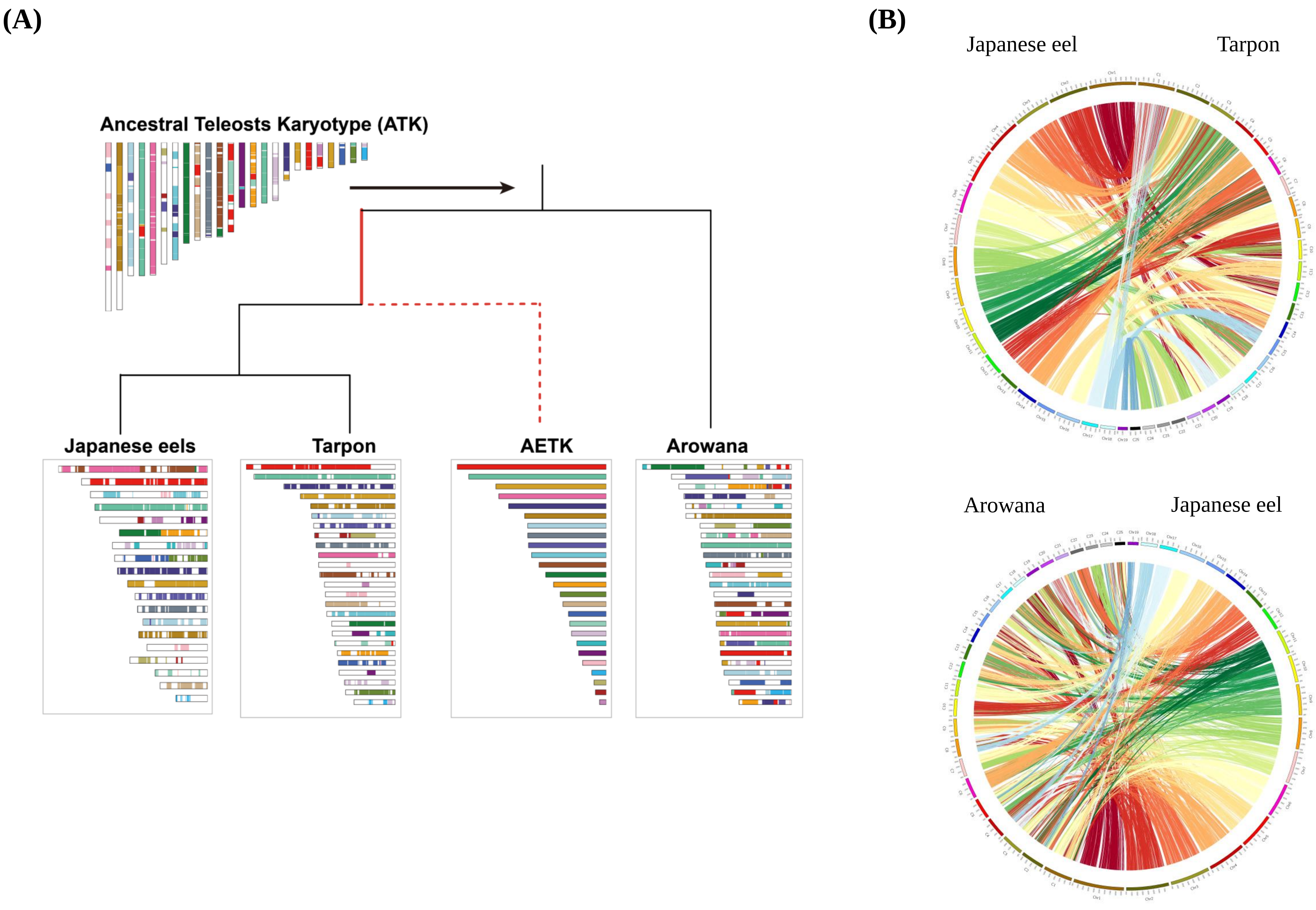
(A)
Ancestral Teleosts Karyotype (ATK)
Japanese eels
Tarpon
AETK
Arowana
(B)
Japanese eel
Tarpon
Arowana
Japanese eel

Supplementary Figures 1-10

Click here to access/download
Supplementary Material
Supplementary Figures 1-10.pptx

Supplementary Tables 1-10

Click here to access/download

Supplementary Material

SupplementaryTables 1-10.docx

Click here to access/download
Supplementary Material
Supplementary Tables 11-12.xlsx

Click here to access/download
Supplementary Material
SupplementaryTables 13-14.docx

Rebuttal Letter

Click here to access/download;Personal Cover;Response_To_Reviewers.docx

Response to Reviewers
GIGA-D-22-00177R1
"A Chromosome-level Assembly of the Japanese Eel Genome, Insights into Gene Duplication and Chromosomal Reorganization"

We would like to thank both reviewers for their comments. Our reply and address each point are detailed below:

<u>Reviewer #1:</u>

This paper describes a new chromosome-level assembly of the Japanese eel, which could finally supersede the various more fragmented assemblies. The assembly process is perhaps overly complex (many data sources and assembly steps, suppl. figure 3), but the result in general appears to be of high quality, as demonstrated by BUSCO (twice) and alignment to a closely related genome (Anguilla anguilla, suppl. figure 4). Figures 1 and 2, however, contain some inconsistencies:

1. QUERY: Figure 1: track B (nanopore coverage) shows a clear bimodal signal, with large blocks of high (double) coverage. These appear possibly correlated with areas low in gene content (track E). Are these possibly collapsed duplicate regions? That would have a strong effect on the analyses of genome duplication. Do other somewhat comparable data sources, for example PacBio CLR, show this feature?

RESPONSE: A new track C (PacBio CLR coverage) has been added in the revised fig 1, showing this comparable pattern. We believe the high-depth regions of tracks B and C are centromeres, not collapsed duplicates. Therefore, the centromere regions contain a low number of genes (track E).

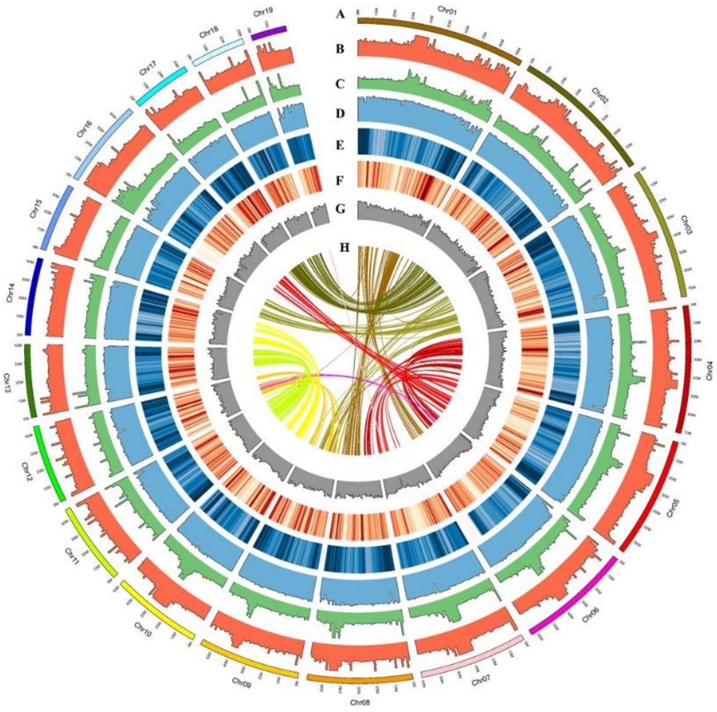

To further verify this point, we have created a Circos plot (below) using Nanopore long reads of European eels (SRR13496139-SRR13496140) and found that the chromosomal level genomes of Japanese eels and European eels were similar. As it is not collapsed duplicate regions, it does not affect genome duplication analysis.

<u>*Anguilla anguilla*</u>

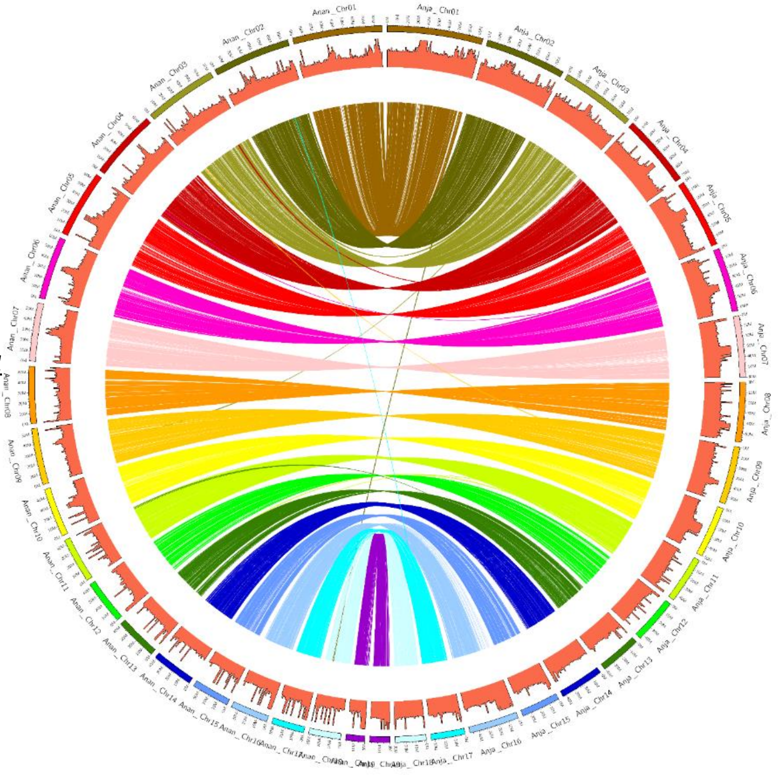

<u>*Anguilla japonica*</u>

The genome landscape (ONT long-reads and Collinear blocks) of Japanese and European Eels.

Even at the T2T level, not all centromere regions are positioned in the middle of the human chromosomes. (**The complete sequence of a human genome.** Science. 2022 376(6588):44-53. doi: 10.1126/science.abj6987).

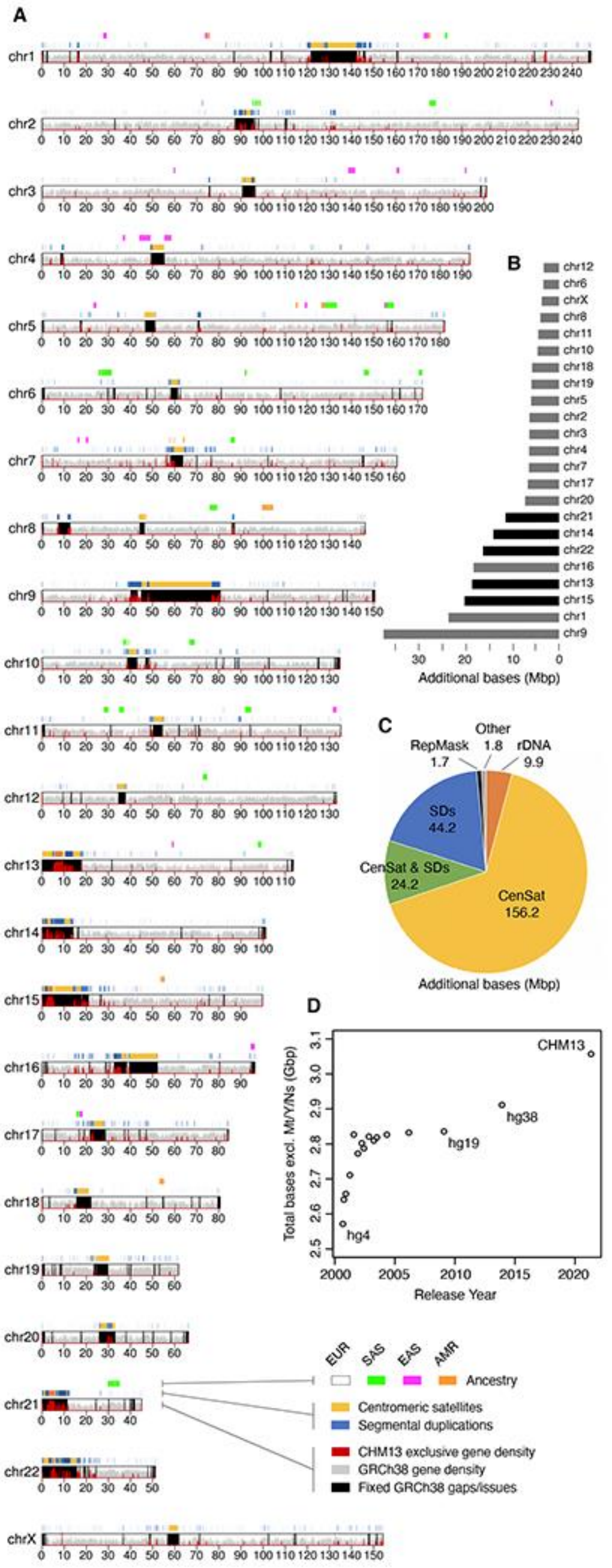

2. ***QUERY 1:*** Figure 2, right panel: the new A. japonica assembly appears to have many unclustered genes (brown), similar to the fragmented draft assembly of A. rostrata and unlike the other included chromosome-level assemblies. This appears to be related to the annotation process? Or are there other problems that preclude orthology assignment for these genes?

RESPONSE: Thank you for this question. We also noticed this question during the annotation process, so we did two ways to evaluate the *annotation process*

1. Busco 94.7 % (assessment mode: proteins)

2. We compared the clustering of the new genome annotation with that of a high-quality close relative, the European eel.

Comparison of clustered and uncluttered gene distributions between *A. Anguilla* and *A. japonica:*

Sample	Single-copy orthologs	Multiple-copy orthologs	Unique paralogs	Other orthologs	Unclustered genes
A. anguilla	3078	8913	142	13374	515
A. japonica	3347	8204	233	12662	5536

We found that the numbers of clustered genes are similar between the two species. These results showed the new *A. japonica* genome annotation is high quality.

Additionally, we used five different tissues of Japanese eel transcriptome (brain, gill, liver, muscle, gonad) [SRA ID: SRR3308117-SRR3308121] to examine these genes and found that most of the unclustered genes were expressed in at least one tissue (92.4%).

We attempted to re-annotate these unclustered sequences using an alternative pipeline. We extracted the unclustered genes and their 1,000 bps flanking regions (500bps from upstream and 500bps from downstream) as input sequences and applied exonerate (https://www.ebi.ac.uk/about/vertebrate-genomics/software/exonerate) to align them to the proteins of European eel.

We deeply investigated the alignments of 5,536 unclustered genes and found 1.there were 3,523 genes can be aligned to the proteins of European eel, but they were still unclustered, suggesting different gene structures may exist between European eel and Japanese eel; 396 genes were less than 100 amino acids, which were too short to be clustered; of the remaining 1,644 genes, 1,505 were supported by at least two of the three annotation methods (homologous, transcriptomic, de novo), that could be new genes or the problems of clustering tools.

We suggest to keep these genes in the manuscript for further investigation. Notice these genes would not affect evolutionary analysis, because only clustered genes were considered in the subsequent analysis.

QUERY 2: And how does A. rostrata get its gain of 11756 genes in this analysis?

RESPONSE: Thanks for the question. Yes, it was our typo. We have corrected the number in the revised manuscript.

QUERY3: (By the way, line 323 has genus Anguilla as +919/-531, the figure +919/-631).

RESPONSE: Thanks for the question. We have double checked and the numbers are consistent in the revised manuscript.

3. ***QUERY 1:*** The discussion of previous and current eel sequencing efforts in the Introduction is not complete. For example, I miss the assemblies by Kai et al (2014) and Nakamura et al (2017) of the Japanese eel genome.

RESPONSE: We have added the references by Kai et al (2014) on the ddRAD and Nakamura et al (2017) on 454FLX-HiSeq in the revised manuscript.

QUERY 2: In addition, the Introduction and Discussion (lines 415-417) present the current assembly as the first chromosome-scale Anguilla genome, which is not the case. At least two high-quality assemblies of Anguilla anguilla (European eel) are available, and should be acknowledged: one is by the Vertebrate Genome Project, and this assembly is even used in the manuscript for comparative purposes (line 199). The other has been described in a preprint (Parey et al 2022). Some of the mentioned papers include similar analyses (mostly on evolution after genome duplication and ancestral genome reconstruction, see figure 5).

RESPONSE: We have quoted the references (Parey 2022, & Rhie et al 2021, Pubmed ID 33911273)

4. ***QUERY****:* The different statistics listed for each alternative assembly in the Introduction make comparisons difficult.

RESPONSE: We have added the assembled information with (i) genome size, (ii) N50 and number of scaffolds extracted from the papers and the link https://www.ncbi.nlm.nih.gov/genome/browse#!/eukaryotes/Anguilla, in the revised manuscript.

5. ***QUERY***: The statement in line 79, that eels as the most basal teleost group are 'close' to non-teleosts, is incorrect. They are just as close to non-teleosts as any other teleost. (The rest of the sentence, up to line 82, could use rephrasing).

RESPONSE: We have rephrased the sentence.

"*From the evolutionary perspective, eels are among the most basal extant groups of teleost fishes after the 3-round of whole genome duplication (WGD). The non-teleost ray-finned fishes, including holostei (bowfin, gar), chondrostei (sturgeon, paddlefish, starlet), and cladistia (bichir, ropefish), diverged from lobe-finned fishes (coelacanth, lungfish) about 450 million years ago (Hurley et al. 2007).*"

6. ***QUERY***: The statement in line 307 that 'Japanese eels are phylogenetically closer to American than European eels' contradicts the phylogeny presented (fig. 2), or is this based on some additional analysis (a density plot not shown), or even on figure 2 right panel (see comment earlier)? Even if they are incrementally 'closer' by some metric, I would not interpret this a phylogenetic

distance, given the inferred divergence dates. In any case, the American eel assembly is still highly fragmented, and not the best basis for inferences which otherwise rely on chromosome-scale assemblies.

RESPONSE: We agree that the American eel assembly is still highly fragmented, and not the best basis for inferences which otherwise rely on chromosome-scale assemblies. So we have removed this statement.

7. ***QUERY***: Similarly, the statements on divergence between teleost groups in lines 495-500 need rephrasing. Anguilla species did not diverge from Megalops etc.

RESPONSE: We have rephrased the sentence.

"The chromosome number of Anguilla species (n=19) was reduced as compared with Megalops cyprinoides (n=25) and Scleropages formosus (n=25)."

8. ***QUERY 1***: Figure 2 & lines 205-213/310-313: These divergence times are calibrated using a few intervals taken from TimeTree.org (red dots). I wonder how reliable this is, as I get quite different intervals when checking now: for Anguilla-Megalops it is 162.2-197.3 (the paper has 179.3-219.3).

RESPONSE: Our apologies for the error in the description of "divergence times" in the method section. In the original manuscript, the "divergence times" in the method section were calculated by us, not directly adopted from TimeTree.org. We accidentally put our calculated data instead of the data from TimeTree.org in the method.

Therefore, in the revised manuscript, we have corrected the "divergence time" in the method section, using values from Timetree.org as the direct input data for subsequent analysis. From the input data 162.2 - 197.3 Mya of Anguilla-Megalops (from TimeTree.org) and our phylogenetic tree analysis, we calculated the divergence time 179.3-219.3 Mya (Anguilla-Megalops) in Figure 2.

QUERY 2: Also TimeTree appears to have arowana (Scleropages) as the most basal branch among the teleosts, the paper has a combined Osteoglossomorpha (arowana) /Elopomorpha(eels) branch. Has the phylogenetic tree topology been inferred or imposed? Why have the specific calibration points been chosen? The early branching among teleosts (see line 310-312) is somewhat controversial, see the preprint by Parey et al.

RESPONSE: Our topology is in agreement with Parey et al's conclusions, supporting Elopomorpha and Osteoglossomorpha as sister groups (the Eloposteoglossocephala clade). We have quoted this paper in the revised manuscript.

9. ***QUERY***: Line 346-348: This uses the eel genome size (~1 Gbp) and the further (4R) duplicated salmon genome (3 Gbp) to argue against a such further genome duplication in eels. Although I agree that the eel 4R probably did not occur, comparing genome sizes presents no evidence in this case. Genome size changes by other processes as well, and more dramatically (e.g. transposon proliferation). In addition, salmon and eel are not closely related, at all. Compare this to the genomes of the (much more closely related) common carp and zebrafish, both ~1.5 Gbp: the

carp genome, but not zebrafish, has experienced an additional duplication, but the zebrafish genome contains a higher transposon density.

RESPONSE: Thanks for your advice. Since this argument is not affirmative, we have removed this sentence.

10. ***QUERY***: The second argument against 4R (lines 352-356, figure 4b) also does not really work. With 8 Hox clusters, the eel genome appears duplicated with respect to the gar (4 clusters), and not quadruplicated. However, with 8 clusters and 70+ genes, eels actually have more than all established 3R teleost genomes (max. 7 clusters, 42-50 genes). So the question is then whether these 8 clusters form nice 3R WGD ohnolog pairs, or if some clusters have been lost (as in nearly all other teleosts) and re-duplicated. The former hypothesis is consistent with the high level of retained WGD genes (line 369), the latter with the inferred high level of local duplication (line 363). The observation of duplicate eel Hox clusters goes back to the initial European eel genome assembly (Henkel et al 2012), but there the draft status precluded confident assignment to 3R for some clusters.

RESPONSE: The revised manuscript includes a genome comparison (highlighting Hox gene clusters) between Japanese eel and zebrafish in Figure 4B. The zebrafish has undergone 3R WGD with 7 Hox gene clusters (lacking HoxDb). By identifying ohnolog pairs using collinear blocks of 10 genes, we found that six of the seven Hox clusters of zebrafish exhibit ohnolog pairs with eels. Because zebrafish HoxBb gene clusters only contain four genes, eel HoxBb and zebrafish HoxBb did not show the ohnolog pair. Comparing Japanese eel to gar, however, showed that 8 Hox clusters form nice 3R WGD ohnolog pairs. As a result, we assume that the eight Hox clusters in Japanese eel are evidence of 3R WGD.

11. ***QUERY***: The eel olfactory receptors have previously been identified using an assembled transcriptome (Churcher et al. 2015, not cited). How do the analyses of line 214-229/324-333/420-434/figure 3 compare?

a. Churcher et al (2015) Deep sequencing of the olfactory epithelium reveals specific chemosensory receptors are expressed at sexual maturity in the European eel *Anguilla anguilla*. Molecular Ecology 24, 822-834. https://doi.org/10.1111/mec.13065

RESPONSE: In 2015 Churcher et al. used transcriptomic data to obtain 112 full-length olfactory receptor (OR) transcripts in European eels, while our study identified 392 OR genes based on the whole genome sequencing data. After reading this paper, we found that the transcript sequences for the olfactory receptor were not included. We then contacted the corresponding authors, who provided us 278 OR transcripts that they had identified in their transcriptome assembly (but many are only partially assembled). The paper shows 112 transcripts for olfactory receptors that cover at least six of the seven predicted transmembrane structural domains. However, these 112 full-length OR transcripts, lacked specific identification. To identify structural domains, we used NCBI's conserved domain database. The 107 transcripts with the full seven predicted transmembrane domains were obtained. The 107 full-length olfactory receptor transcripts were then categorized and compared (below).

	alpha	beta	gamma	delta	epsilon	zeta	eta	Total
In 2015 Churcher et al	0	2	5	74	11	13	2	107
This study	0	1	26	176	5	151	33	392

In the transcriptome assembly (Churcher's paper), the identification of OR transcripts were restricted to three experimental conditions, in which the two conditions (freshwater & seawater, sex is not specified) and sexually matured stage (male fish only). The study identified the differential expression of OR genes in certain developmental windows of the fish. However, the study is interesting to characterize the expression of OR and other chemosensory receptors. We have quoted this reference in the revised manuscript.

12. ***QUERY***: Lines 460-467 state eels have retained duplicates of immune genes, which have been under positive selection. So how does this translate to a (very recent) negative effect on eel fitness (line 460-462)?

RESPONSE: We assume this is the issue of cause-and-effects. The increase of environmental pathogens increased the positive selection pressure of retaining duplicated immune genes.

13. ***QUERY***: The discussion of line 482-502 on chromosome numbers invokes ecological explanations (freshwater vs. marine habitats, 482-489), but subsequently does not translate this to the low Anguilla chromosome numbers. As these ecological factors are highly applicable to Anguillidae, this connection should be explored here - including their evolutionary history (e.g. Inoue et al, 2010, Deep-ocean origin of the freshwater eels. Biology Letters 6, https://doi.org/10.1098/rsbl.2009.0989)

RESPONSE. We have studied the paper and added the information of the oceanic origin of freshwater eels and the reference.

14. ***QUERY***: In this discussion: how do the numbers of line 482/3 (modal 2n 54/48 chromosomes in fish) correspond to those of line 492 (peak chromosome number n = 24/25 in extant teleosts)?

RESPONSE: The modal number is the most frequent number identified from 103 species of fishes. The chromosome numbers range from 24 -100 (2n). The numbers do not correspond exactly to the peak chromosome number.

15. ***QUERY***: The supplementary figures/tables lack legends (just mentions in the main text).

RESPONSE: Thanks. The legends of supplementary tables and figures have been added in the main text of the revised manuscript.

16. ***QUERY***: *Line 109: which ONT flowcell, kit, and basecaller versions have been used?*

RESPONSE: The type of ONT flowcell (R9.4.1), the kits (LSK109) and the basecaller vesions is (Gyppy 3.2.10). We have added the information to the revised manuscript.

Custom Review Question(s):

QUERY: I am a co-author on a preprint that I think the authors should have cited (Parey et al. (2022) Genome structures resolve the early diversification of teleost fishes. BioRxiv https://doi.org/10.1101/2022.04.07.487469). Other than that, I declare that I have no competing interests.

RESPONSE: The paper has been cited in the revised manuscript.

Reviewer #2:

This manuscript by WANG et al. titled "A Chromosome-level Assembly of the Japanese Eel Genome, Insights into Gene Duplication and Chromosomal Reorganization" provides a high quality genome assembly of Japanese Eel, and economically important fish. The authors have used for kinds of sequencing technologies, and assembling strategies, and provided well annotated genomes. This genome provides useful information for the genome organization and evolution and other fields of this species.

Overall, the manuscript is sufficiently descriptive and easy to follow. I have three major concerns:

1. ***QUERY***: The genome annotation rely on the transcriptome. No detailed information was given the method section.

RESPONSE: We are sorry for this missing part. The ID of the data source (Bioproject: PRJNA578238) has been added in the revised manuscript.

2. ***QUERY***: *The analyses do not include command lines or software versions and thus are not repeatable easily. A document that include these information is higly recommended included as a supplementary file.*

RESPONSE: Thanks for your advice. We have added the software versions in the Method part of the revised manuscript.

QUERY: The genome assembly seems has not been released on NCBI database (https://www.ncbi.nlm.nih.gov/bioproject/?term=PRJNA852364). Besides, the gene models (nucleotide, protein, and GFF files) should also be made available and included in the Data Availability section when the manuscript is accepted.

RESPONSE: The genome assembly and raw sequencing reads are now publicly available on NCBI databases under the accession number PRJNA852364. The gene models are available at https://zenodo.org/record/7099450.
