# Supplementary material for: A Chromosome-level assembly of the Japanese eel genome, insights into gene duplication and chromosomal reorganization
Source: Gigascience. 2022 Dec 8;11:giac120. doi: 10.1093/gigascience/giac120 (PMC9730501; doi:10.1093/gigascience/giac120)
Supplement: giac120_Supplemental_Figures_and_Tables [file giac120_supplemental_figures_and_tables.zip › Supplementary Figures 1-10.pptx]

## Slide 1
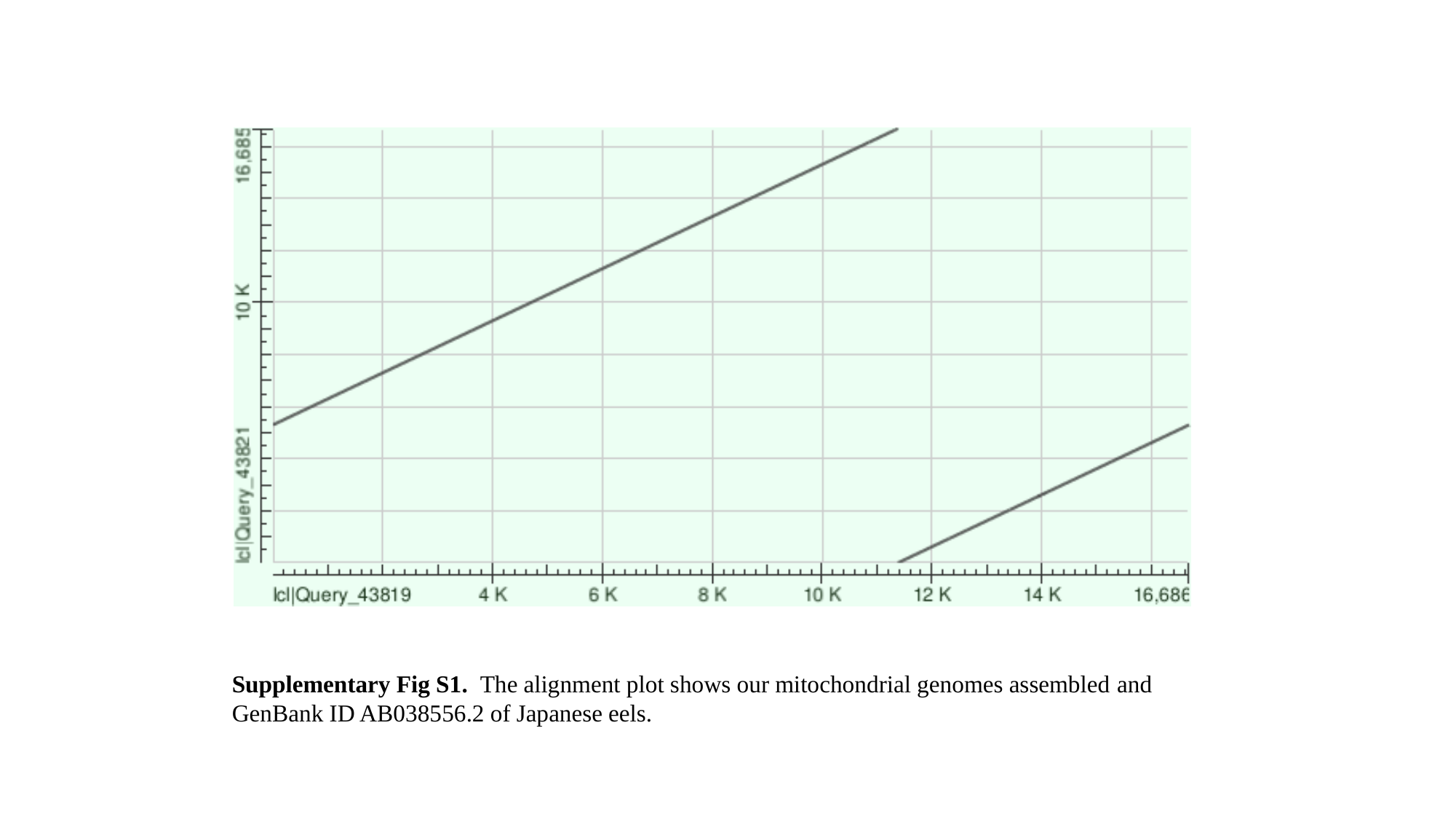

Supplementary Fig S1. The alignment plot shows our mitochondrial genomes assembled and GenBank ID AB038556.2 of Japanese eels.

## Slide 2
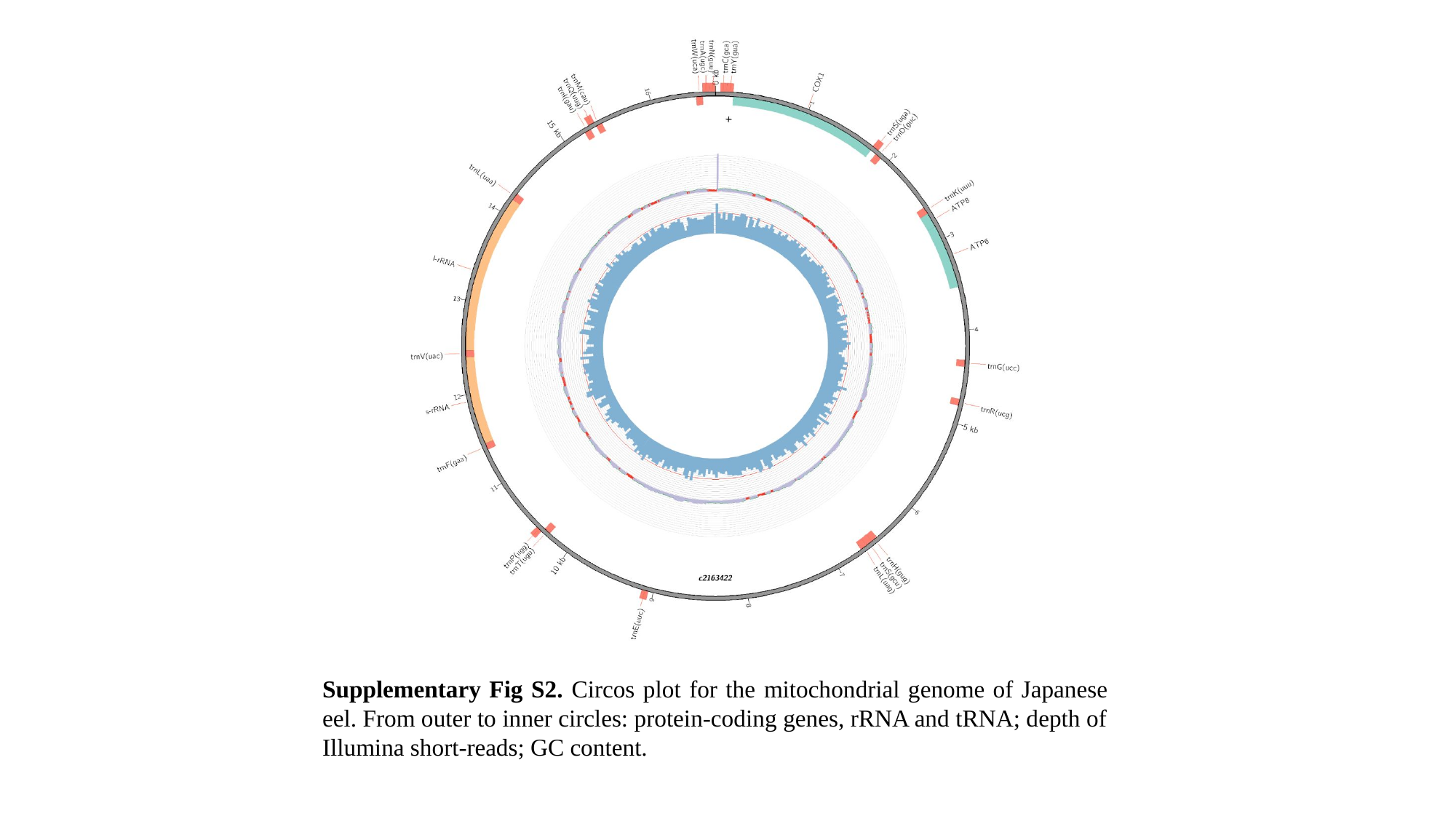

Supplementary Fig S2. Circos plot for the mitochondrial genome of Japanese eel. From outer to inner circles: protein-coding genes, rRNA and tRNA; depth of Illumina short-reads; GC content.

## Slide 3
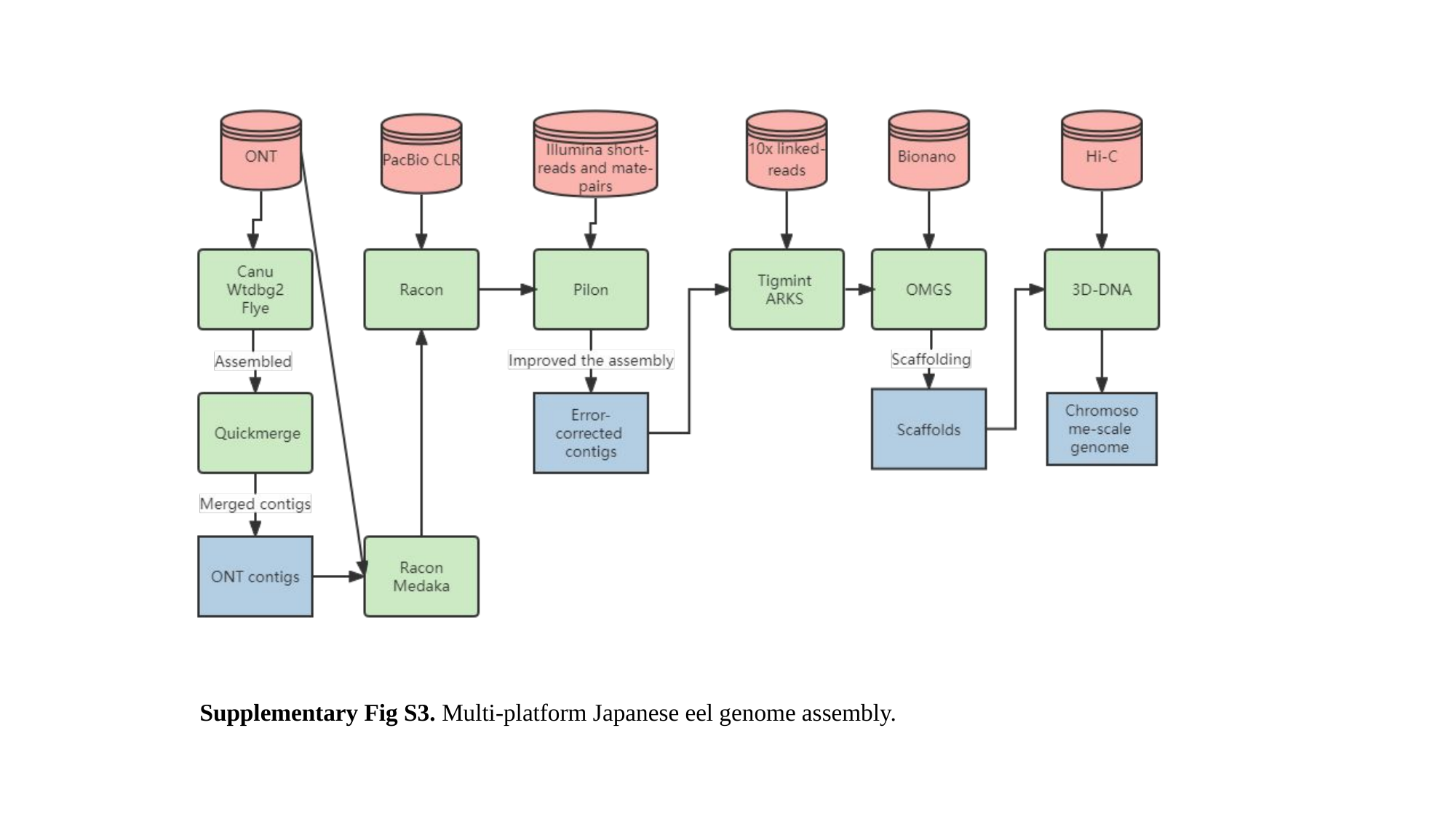

Supplementary Fig S3. Multi-platform Japanese eel genome assembly.

## Slide 4
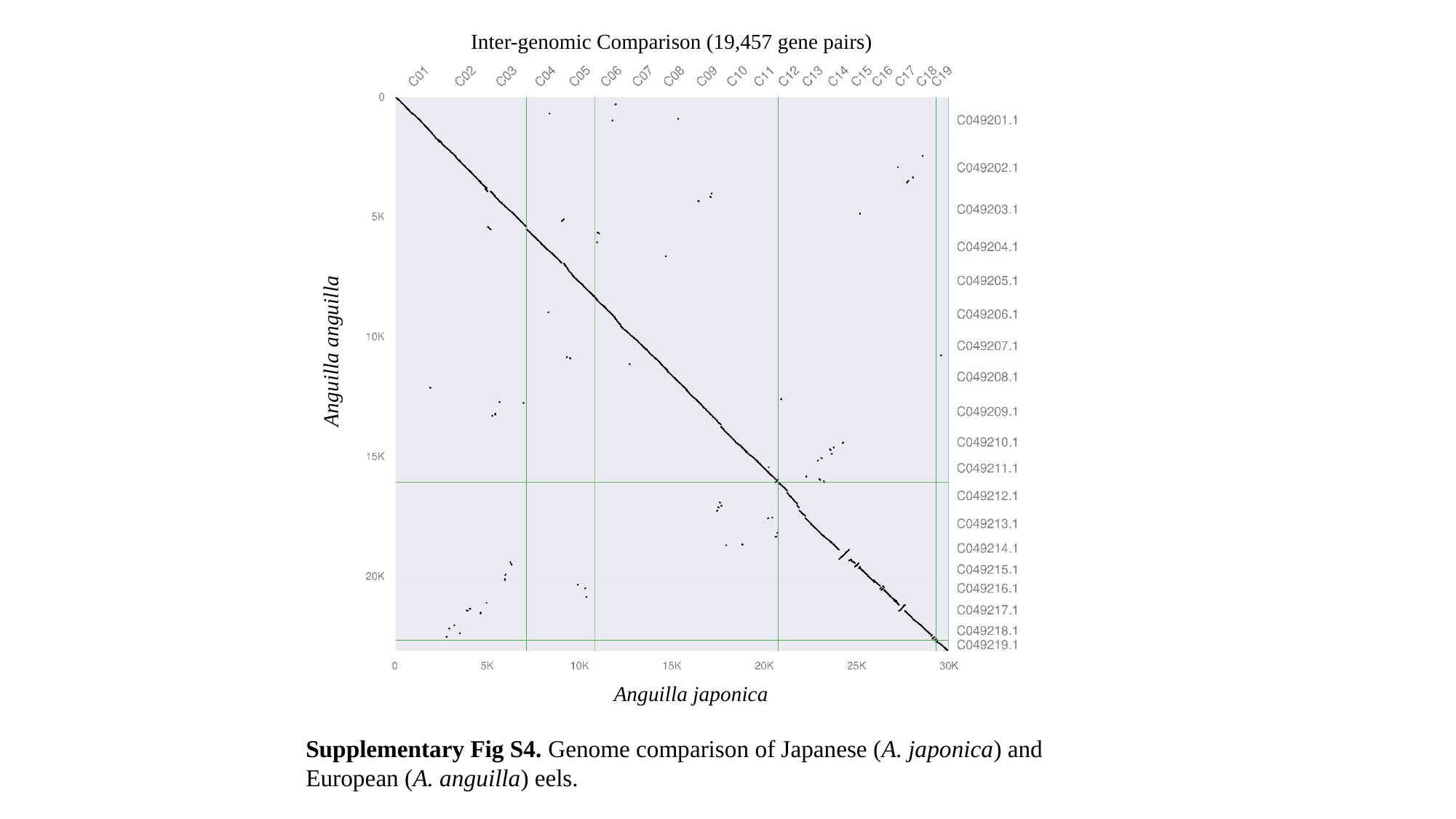

Inter-genomic Comparison (19,457 gene pairs)
Anguilla anguilla
Anguilla japonica
Supplementary Fig S4. Genome comparison of Japanese (A. japonica) and European (A. anguilla) eels.

## Slide 5
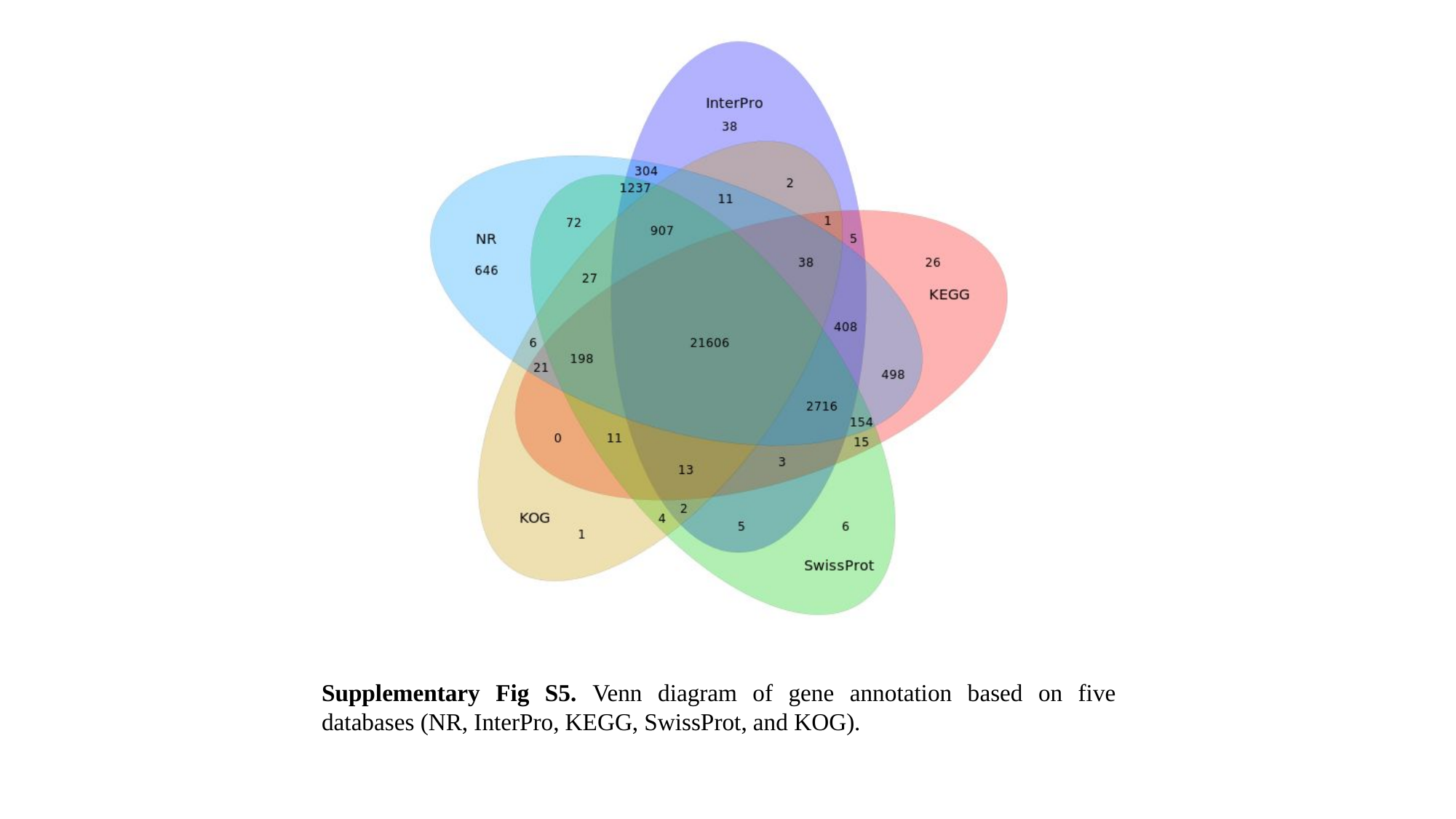

Supplementary Fig S5. Venn diagram of gene annotation based on five databases (NR, InterPro, KEGG, SwissProt, and KOG).

## Slide 6
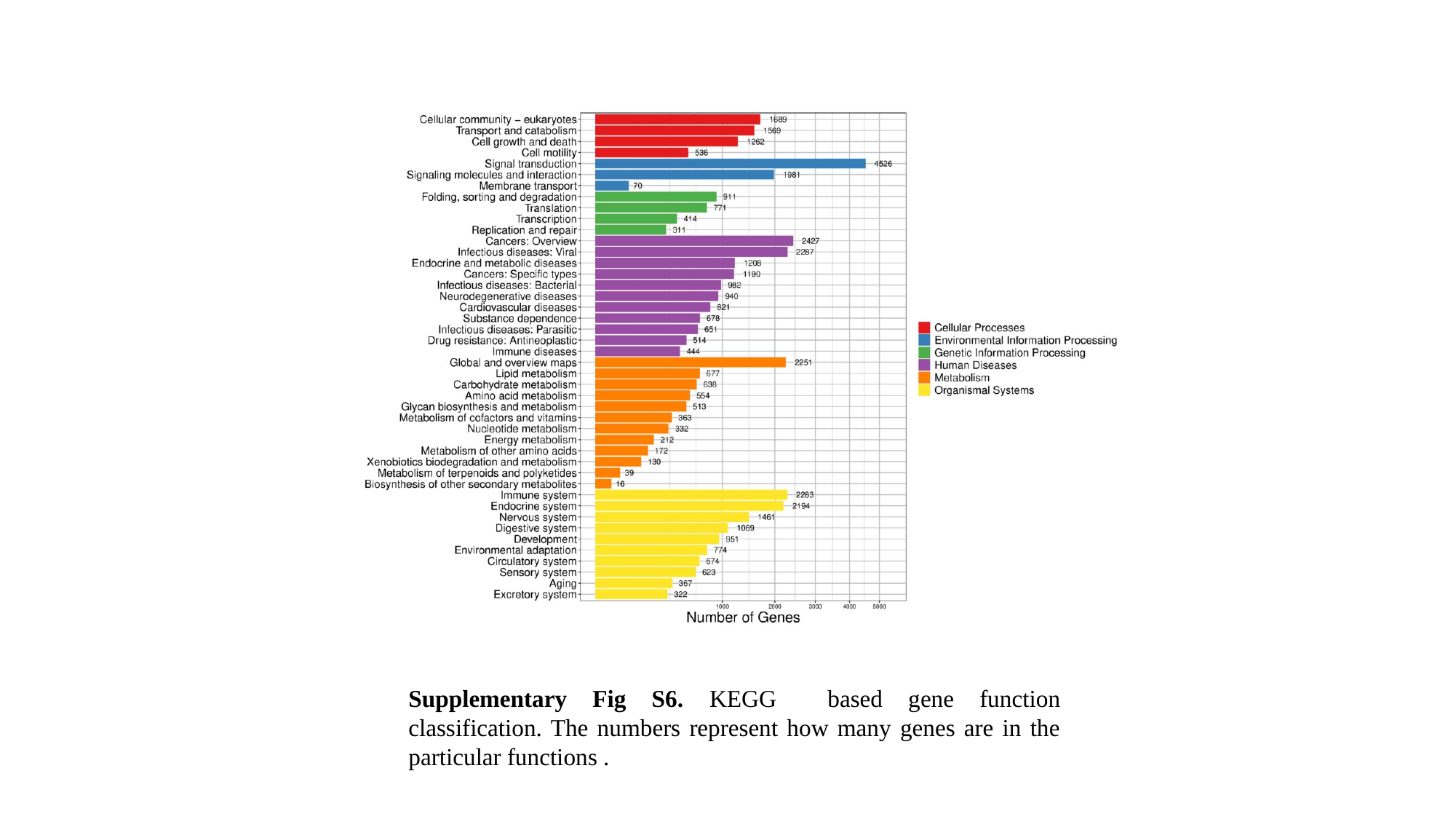

Supplementary Fig S6. KEGG based gene function classification. The numbers represent how many genes are in the particular functions .

## Slide 7
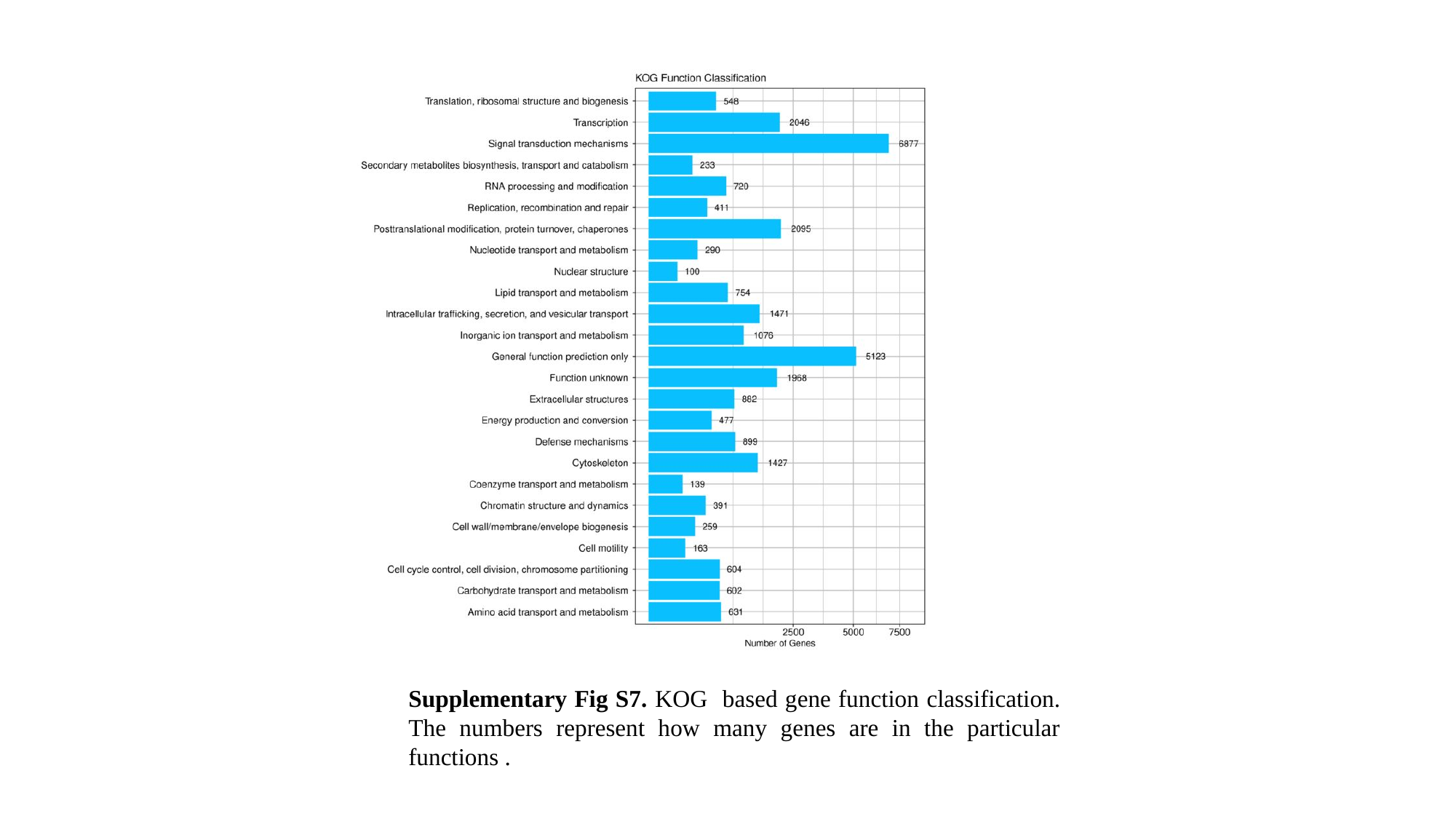

Supplementary Fig S7. KOG based gene function classification. The numbers represent how many genes are in the particular functions .

## Slide 8
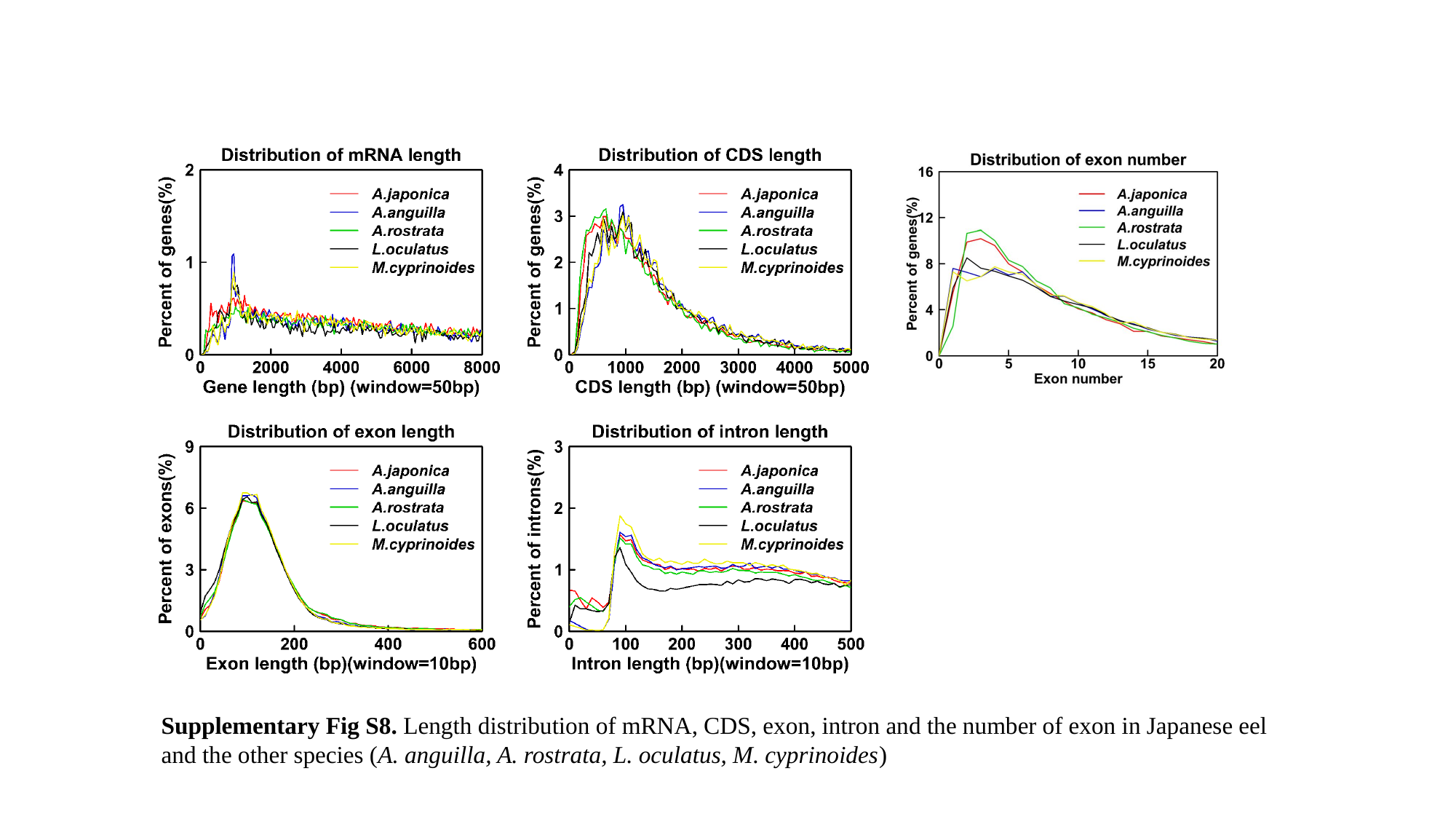

Supplementary Fig S8. Length distribution of mRNA, CDS, exon, intron and the number of exon in Japanese eel and the other species (A. anguilla, A. rostrata, L. oculatus, M. cyprinoides)

## Slide 9
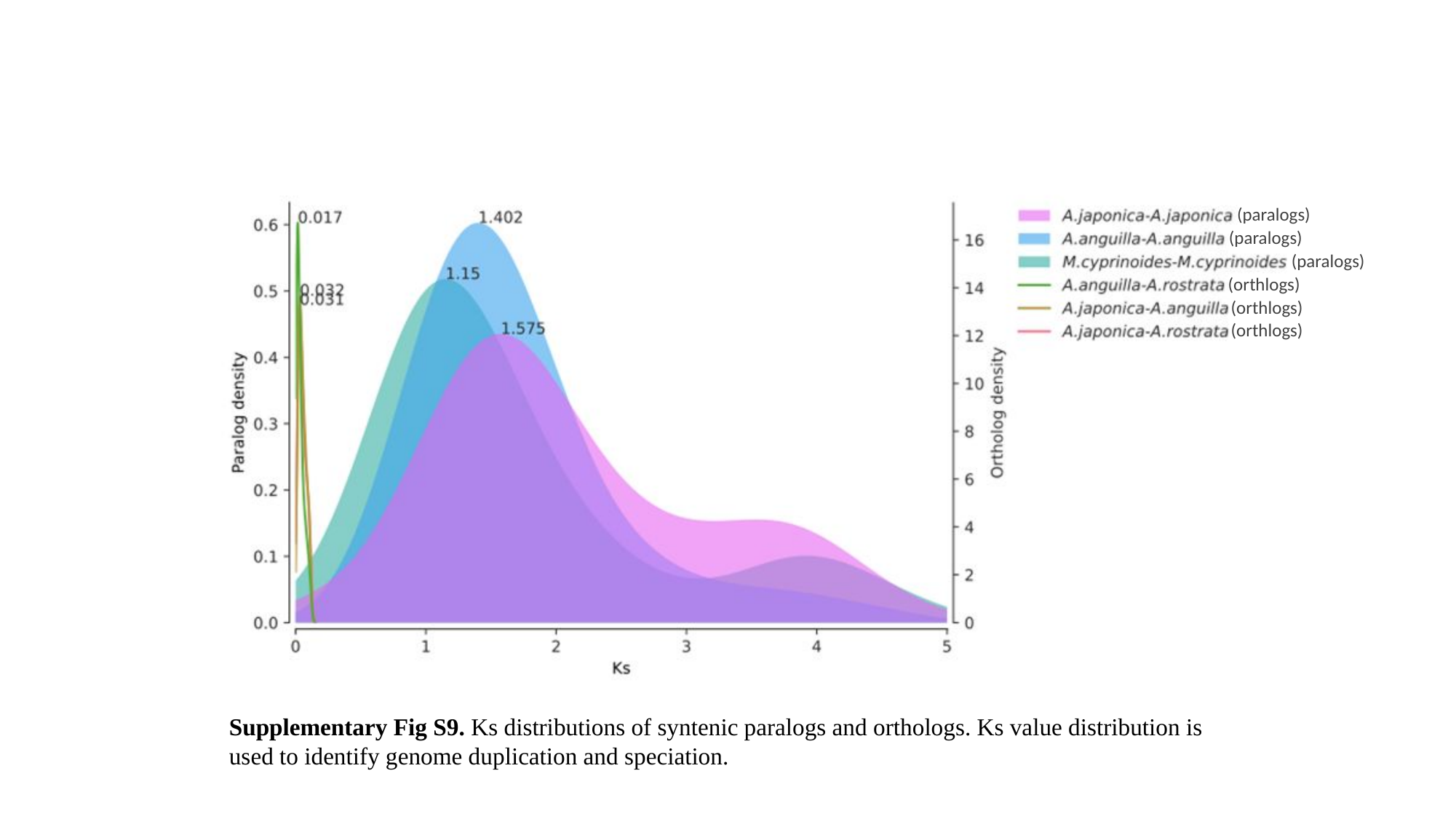

(paralogs)
(paralogs)
(paralogs)
(orthlogs)
(orthlogs)
(orthlogs)
Supplementary Fig S9. Ks distributions of syntenic paralogs and orthologs. Ks value distribution is used to identify genome duplication and speciation.

## Slide 10
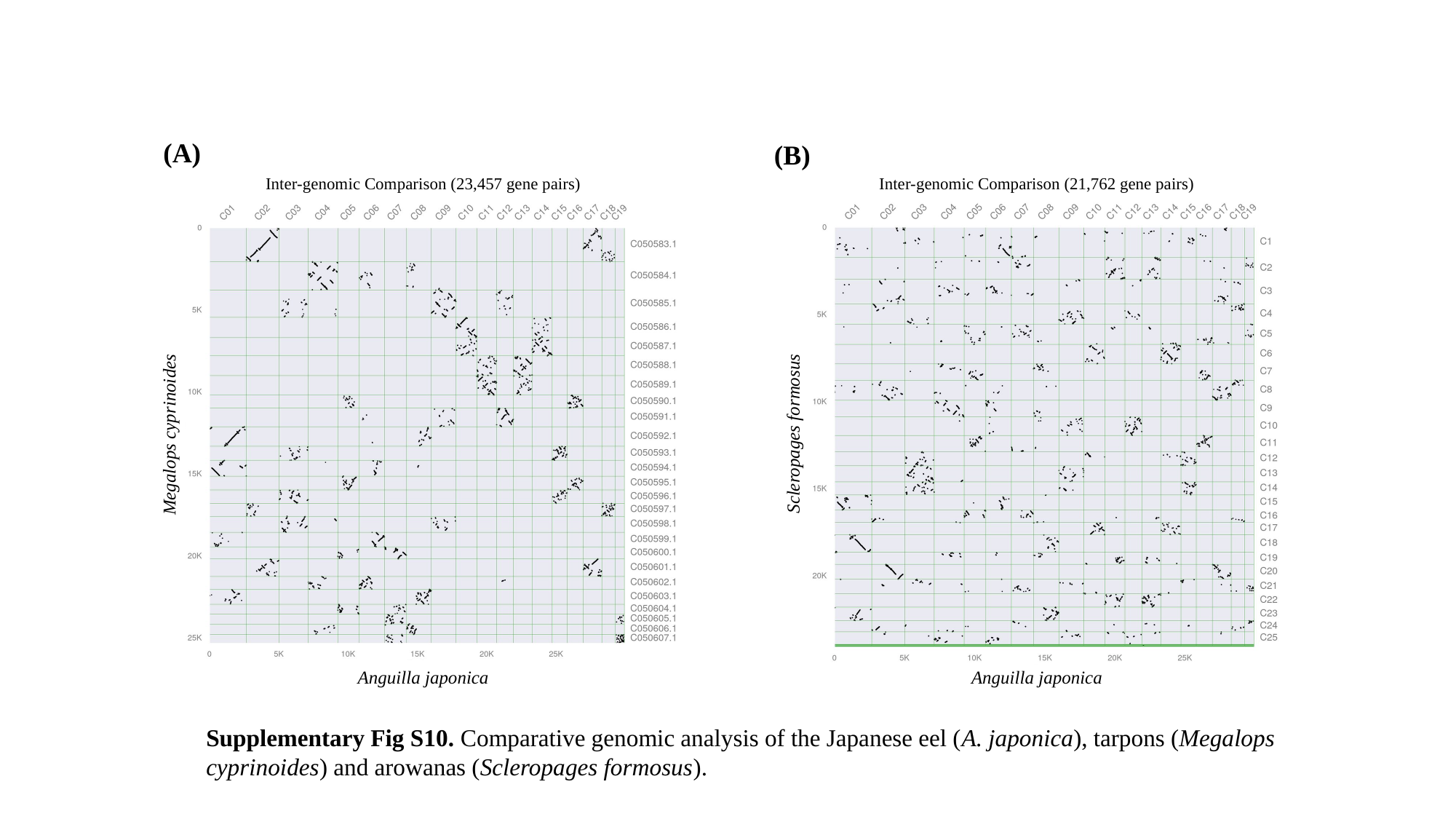

(A)
(B)
Inter-genomic Comparison (23,457 gene pairs)
Megalops cyprinoides
Anguilla japonica
Inter-genomic Comparison (21,762 gene pairs)
Scleropages formosus
Anguilla japonica
Supplementary Fig S10. Comparative genomic analysis of the Japanese eel (A. japonica), tarpons (Megalops cyprinoides) and arowanas (Scleropages formosus).
