# Supplementary material for: A Chromosome-level assembly of the Japanese eel genome, insights into gene duplication and chromosomal reorganization
Source: Gigascience. 2022 Dec 8;11:giac120. doi: 10.1093/gigascience/giac120 (PMC9730501; doi:10.1093/gigascience/giac120)
Supplement: giac120_Supplemental_Figures_and_Tables [file giac120_supplemental_figures_and_tables.zip › SupplementaryTables 13-14.docx]

**Supplementary Table S13.** The karyotypes of *M. cyprinoides* (tarpons) and the common ancestor of eels and tarpons (AETK).

| *M. cyprinoides* | AETK |
| --- | --- |
| Chr01 | AETK_pChr1 |
| Chr02 | AETK_pChr2 |
| Chr03 | AETK_pChr5 |
| Chr04 | AETK_pChr3 |
| Chr05 | AETK_pChr6,AETK_pChr3,AETK_pChr6 |
| Chr06 | AETK_pChr7,AETK_pChr9,AETK_pChr7 |
| Chr07 | AETK_pChr9 |
| Chr08 | AETK_pChr23,AETK_pChr24 |
| Chr09 | AETK_pChr8 |
| Chr10 | AETK_pChr4 |
| Chr11 | AETK_pChr21 |
| Chr12 | AETK_pChr11 |
| Chr13 | AETK_pChr25 |
| Chr14 | AETK_pChr21 |
| Chr15 | AETK_pChr15 |
| Chr16 | AETK_pChr10 |
| Chr17 | AETK_pChr12 |
| Chr18 | AETK_pChr19,AETK_pChr20 |
| Chr19 | AETK_pChr1,AETK_pChr17 |
| Chr20 | AETK_pChr13 |
| Chr21 | AETK_pChr16,AETK_pChr4,AETK_pChr16 |
| Chr22 | AETK_pChr20 |
| Chr23 | AETK_pChr18 |
| Chr24 | AETK_pChr14 |
| Chr25 | AETK_pChr18,AETK_pChr22 |

**Supplementary Table S14.** The karyotypes of *A. japonica* (Japanese eel) and the common ancestor of eels and tarpons (AETK).

| ***A. japonica*** | **AETK** |
| --- | --- |
| Chr01 | AETK_pChr4,AETK_pChr12,AETK_pChr11,AETK_pChr4,AETK_pChr11,AETK_pChr4,AETK_pChr11 |
| Chr02 | AETK_pChr1 |
| Chr03 | AETK_pChr10,AETK_pChr21,AETK_pChr10 |
| Chr04 | AETK_pChr2,AETK_pChr13,AETK_pChr2 |
| Chr05 | AETK_pChr20,AETK_pChr25,AETK_pChr24 |
| Chr06 | AETK_pChr13,AETK_pChr12, |
| Chr07 | AETK_pChr19,AETK_pChr18,AETK_pChr19,AETK_pChr18 |
| Chr08 | AETK_pChr14,AETK_pChr16,AETK_pChr4,AETK_pChr16 |
| Chr09 | AETK_pChr5 |
| Chr10 | AETK_pChr3 |
| Chr11 | AETK_pChr9 |
| Chr12 | AETK_pChr8 |
| Chr13 | AETK_pChr7,AETK_pChr9,AETK_pChr7 |
| Chr14 | AETK_pChr6 |
| Chr15 | AETK_pChr21 |
| Chr16 | AETK_pChr24,AETK_pChr23 |
| Chr17 | AETK_pChr17,AETK_pChr1,AETK_pChr17 |
| Chr18 | AETK_pChr15 |
| Chr19 | AETK_pChr22 |
