# Supplementary material for: A Chromosome-level assembly of the Japanese eel genome, insights into gene duplication and chromosomal reorganization
Source: Gigascience. 2022 Dec 8;11:giac120. doi: 10.1093/gigascience/giac120 (PMC9730501; doi:10.1093/gigascience/giac120)
Supplement: giac120_Response_to_Reviewer_Comments_Original_Submission [file giac120_response_to_reviewer_comments_original_submission.pdf]

Author's Response To Reviewer Comments

Close

We would like to thank the editor and both reviewers for their comments. We have addressed all the comments. New figures and revision have been made. Our reply to individual questions are listed in a document which has been attached (rebuttal letter). Thanks

Close
