# Supplementary material for: A Chromosome-level assembly of the Japanese eel genome, insights into gene duplication and chromosomal reorganization
Source: Gigascience. 2022 Dec 8;11:giac120. doi: 10.1093/gigascience/giac120 (PMC9730501; doi:10.1093/gigascience/giac120)
Supplement: giac120_Response_to_Reviewer_Comments_Revision_1 [file giac120_response_to_reviewer_comments_revision_1.pdf]

Author's Response To Reviewer Comments

Close

Thanks for the additional comments.
We have made the corresponding or clarification as per your suggestion.

Query: Line 79: 'The non-teleost teleost ray-finned fishes, including holostei (bowfin, gar), chondrostei (sturgeon, paddlefish, starlet), and cladistia (bichir, ropefish), diverged from lobe-finned fishes (coelacanth, lungfish) about 450 million years ago (Hurley et al. 2007).'
I suggest 'The ray-finned fishes, including the non-teleost holostei (bowfin, gar), chondrostei...'

Response: Revised as per you suggestion.

Query: Line 311: 'American and European eels diverged from their ancestors about 27.0 million years ago (MYA)' -> common ancestor
Line 364: 'Collectively, the data do not support the presence of 4R-WGD in Japanese' -> Japanese eel

Response: Revised as per you suggestion.

Query: Line 470: 'The duplicated genes might have strengthened immune-related responses against different pathogens, contributing to the decline of eel populations (Danne et al. 2022; 472 Bandin et al. 2014; Kennedy 2007). Presumably, physiological fitness for adaptation might have been weakened by changes in the ecological environment, causing these evolutionary novelties (Belyayev 2014). Notably, the positive selection of immune-related genes indicates the adaptive advantages of the additional TD and PD duplication.'
I commented on this before - it is not entirely clear how very recent (decline of populations) and evolutionary changes in deeper time (adaptive advantages of duplication) are causally intertwined with 'changes in the ecological environment'. This suggests the duplications and evolutionary novelties are the result of recent ecological changes associated with population decline, which is probably not what you meant?

Response: I agree that the meaning of the sentence is confusing. We have revised and deleted the phrase "contributing to the decline of populations".
The new paragraph is
"The duplicated genes might have strengthened immune-related responses against different pathogens (Danne et al. 2022; Bandin et al. 2014; Kennedy 2007). These evolutionary novelties could be attributed to changes in the ecological environment challenging physiological fitness for adaptation (Belyayev 2014). Notably, the positive selection of immune-related genes indicates the adaptive advantages of the additional TD and PD duplication."

Close
