# Supplementary material for: A Chromosome-level assembly of the Japanese eel genome, insights into gene duplication and chromosomal reorganization
Source: Gigascience. 2022 Dec 8;11:giac120. doi: 10.1093/gigascience/giac120 (PMC9730501; doi:10.1093/gigascience/giac120)
Supplement: giac120_Reviewer_1_Report_Original_Submission — Christiaan Henkel -- 8/9/2022 Reviewed [file giac120_reviewer_1_report_original_submission.pdf]

Reviewer Report

Title: A Chromosome-level Assembly of the Japanese Eel Genome, Insights into Gene Duplication and Chromosomal Reorganization

Version: Original Submission **Date:** 8/9/2022

Reviewer name: Christiaan Henkel

Reviewer Comments to Author:

This paper describes a new chromosome-level assembly of the Japanese eel, which could finally supersede the various more fragmented assemblies. The assembly process is perhaps overly complex (many data sources and assembly steps, suppl. figure 3), but the result in general appears to be of high quality, as demonstrated by BUSCO (twice) and alignment to a closely related genome (Anguilla anguilla, suppl. figure 4). Figures 1 and 2, however, contain some inconsistencies:Figure 1: track B (nanopore coverage) shows a clear bimodal signal, with large blocks of high (double) coverage. These appear possibly correlated with areas low in gene content (track E). Are these possibly collapsed duplicate regions? That would have a strong effect on the analyses of genome duplication. Do other somewhat comparable data sources, for example PacBio CLR, show this feature?Figure 2, right panel: the new A. japonica assembly appears to have many unclustered genes (brown), similar to the fragmented draft assembly of A. rostrata and unlike the other included chromosome-level assemblies. This appears to be related to the annotation process? Or are there other problems that preclude orthology assignment for these genes? And how does A. rostrata get its gain of 11756 genes in this analysis? (By the way, line 323 has genus Anguilla as +919/-531, the figure +919/-631).Some other questions and comments I would like the authors to address:The discussion of previous and current eel sequencing efforts in the Introduction is not complete. For example, I miss the assemblies by Kai et al (2014) and Nakamura et al (2017) of the Japanese eel genome. In addition, the Introduction and Discussion (lines 415-417) present the current assembly as the first chromosome-scale Anguilla genome, which is not the case. At least two high-quality assemblies of Anguilla anguilla (European eel) are available, and should be acknowledged: one is by the Vertebrate Genome Project, and this assembly is even used in the manuscript for comparative purposes (line 199). The other has been described in a preprint (Parey et al 2022). Some of the mentioned papers include similar analyses (mostly on evolution after genome duplication and ancestral genome reconstruction, see figure 5).* Kai et al (2014) A ddRAD-based genetic map and its integration with the genome assembly of Japanese eel (Anguilla japonica) provides insights into genome evolution after the teleost-specific genome duplication. BMC Genomics 15, 233. https://doi.org/10.1186/1471-2164-15-233* Nakamura et al (2017) Rhodopsin gene copies in Japanese eel originated in a teleost-specific genome duplication. Zoological Lett 3, 18. https://doi.org/10.1186/s40851-017-0079-2* Parey et al. (2022) Genome structures resolve the early diversification of teleost fishes. BioRxiv https://doi.org/10.1101/2022.04.07.487469The different statistics listed for each alternative assembly in the Introduction make comparisons difficult.The statement in line 79, that eels as the most basal teleost group are 'close' to non-teleosts, is incorrect. They are just as close to non-teleosts as any other teleost. (The rest of the sentence, up to line 82, could

use rephrasing).The statement in line 307 that 'Japanese eels are phylogenetically closer to American than European eels' contradicts the phylogeny presented (fig. 2), or is this based on some additional analysis (a density plot not shown), or even on figure 2 right panel (see comment earlier)? Even if they are incrementally 'closer' by some metric, I would not interpret this a phylogenetic distance, given the inferred divergence dates. In any case, the American eel assembly is still highly fragmented, and not the best basis for inferences which otherwise rely on chromosome-scale assemblies.Similarly, the statements on divergence between teleost groups in lines 495-500 need rephrasing. Anguilla species did not diverge from Megalops etc.Figure 2 & lines 205-213/310-313: These divergence times are calibrated using a few intervals taken from TimeTree.org (red dots). I wonder how reliable this is, as I get quite different intervals when checking now: for Anguilla-Megalops it is 162.2-197.3 (the paper has 179.3-219.3). Also TimeTree appears to have arowana (Scleropages) as the most basal branch among the teleosts, the paper has a combined Osteoglossomorpha(arowana)/Elopomorpha(eels) branch. Has the phylogenetic tree topology been inferred or imposed? Why have the specific calibration points been chosen? The early branching among teleosts (see line 310-312) is somewhat controversial, see the preprint by Parey et al.Line 346-348: This uses the eel genome size (~1 Gbp) and the further (4R) duplicated salmon genome (3 Gbp) to argue against a such further genome duplication in eels. Although I agree that the eel 4R probably did not occur, comparing genome sizes presents no evidence in this case. Genome size changes by other processes as well, and more dramatically (e.g. transposon proliferation). In addition, salmon and eel are not closely related, at all. Compare this to the genomes of the (much more closely related) common carp and zebrafish, both ~1.5 Gbp: the carp genome, but not zebrafish, has experienced an additional duplication, but the zebrafish genome contains a higher transposon density.The second argument against 4R (lines 352-356, figure 4b) also does not really work. With 8 Hox clusters, the eel genome appears duplicated with respect to the gar (4 clusters), and not quadruplicated. However, with 8 clusters and 70+ genes, eels actually have more than all established 3R teleost genomes (max. 7 clusters, 42-50 genes). So the question is then whether these 8 clusters form nice 3R WGD ohnolog pairs, or if some clusters have been lost (as in nearly all other teleosts) and re-duplicated. The former hypothesis is consistent with the high level of retained WGD genes (line 369), the latter with the inferred high level of local duplication (line 363). The observation of duplicate eel Hox clusters goes back to the initial European eel genome assembly (Henkel et al 2012), but there the draft status precluded confident assignment to 3R for some clusters.The eel olfactory receptors have previously been identified using an assembled transcriptome (Churcher et al. 2015, not cited). How do the analyses of line 214-229/324-333/420-434/figure 3 compare?* Churcher et al (2015) Deep sequencing of the olfactory epithelium reveals specific chemosensory receptors are expressed at sexual maturity in the European eel Anguilla anguilla. Molecular Ecology 24, 822-834. https://doi.org/10.1111/mec.13065Lines 460-467 state eels have retained duplicates of immune genes, which have been under positive selection. So how does this translate to a (very recent) negative effect on eel fitness (line 460-462)?The discussion of line 482-502 on chromosome numbers invokes ecological explanations (freshwater vs. marine habitats, 482-489), but subsequently does not translate this to the low Anguilla chromosome numbers. As these ecological factors are highly applicable to Anguillidae, this connection should be explored here - including their evolutionary history (e.g. Inoue et al, 2010, Deep-ocean origin of the freshwater eels. Biology Letters 6, https://doi.org/10.1098/rsbl.2009.0989)In this discussion: how do the numbers of line 482/3 (modal 2n 54/48 chromosomes in fish) correspond to

those of line 492 (peak chromosome number n = 24/25 in extant teleosts)?The supplementary figures/tables lack legends (just mentions in the main text).Line 109: which ONT flowcell, kit, and basecaller versions have been used? In the M&M, please list software versions.

Level of Interest

Please indicate how interesting you found the manuscript: Choose an item.

Quality of Written English

Please indicate the quality of language in the manuscript: Choose an item.

Declaration of Competing Interests

Please complete a declaration of competing interests, considering the following questions:

- Have you in the past five years received reimbursements, fees, funding, or salary from an organisation that may in any way gain or lose financially from the publication of this manuscript, either now or in the future?
- Do you hold any stocks or shares in an organisation that may in any way gain or lose financially from the publication of this manuscript, either now or in the future?
- Do you hold or are you currently applying for any patents relating to the content of the manuscript?
- Have you received reimbursements, fees, funding, or salary from an organization that holds or has applied for patents relating to the content of the manuscript?
- Do you have any other financial competing interests?
- Do you have any non-financial competing interests in relation to this paper?

If you can answer no to all of the above, write 'I declare that I have no competing interests' below. If your reply is yes to any, please give details below.

I am a co-author on a preprint that I think the authors should have cited (Parey et al. (2022) Genome structures resolve the early diversification of teleost fishes. BioRxiv https://doi.org/10.1101/2022.04.07.487469). Other than that, I declare that I have no competing interests.

I agree to the open peer review policy of the journal. I understand that my name will be included on my report to the authors and, if the manuscript is accepted for publication, my named report including any attachments I upload will be posted on the website along with the authors' responses. I agree for my report to be made available under an Open Access Creative Commons CC-BY license (http://creativecommons.org/licenses/by/4.0/). I understand that any comments which I do not wish to

be included in my named report can be included as confidential comments to the editors, which will not be published.

Choose an item.

To further support our reviewers, we have joined with Publons, where you can gain additional credit to further highlight your hard work (see: https://publons.com/journal/530/gigascience). On publication of this paper, your review will be automatically added to Publons, you can then choose whether or not to claim your Publons credit. I understand this statement.

Yes Choose an item.
