# Supplementary material for: A Chromosome-level assembly of the Japanese eel genome, insights into gene duplication and chromosomal reorganization
Source: Gigascience. 2022 Dec 8;11:giac120. doi: 10.1093/gigascience/giac120 (PMC9730501; doi:10.1093/gigascience/giac120)
Supplement: giac120_Reviewer_1_Report_Revision_1 — Christiaan Henkel -- 10/6/2022 Reviewed [file giac120_reviewer_1_report_revision_1.pdf]

**Reviewer Report**

**Title: A Chromosome-level Assembly of the Japanese Eel Genome, Insights into Gene Duplication and Chromosomal Reorganization**

**Version: Revision 1** **Date:** 10/6/2022

**Reviewer name: Christiaan Henkel**

**Reviewer Comments to Author:**

I thank the authors for thoroughly addressing all the issues I raised. I am satisfied with their responses, and at this point only have a few small suggestions on phrasing:Line 79: 'The non-teleost teleost ray-finned fishes, including holostei (bowfin, gar), chondrostei (sturgeon, paddlefish, starlet), and cladistia (bichir, ropefish), diverged from lobe-finned fishes (coelacanth, lungfish) about 450 million years ago (Hurley et al. 2007).'I suggest 'The ray-finned fishes, including the non-teleost holostei (bowfin, gar), chondrostei...'Line 311: 'American and European eels diverged from their ancestors about 27.0 million years ago (MYA)' -> common ancestorLine 364: 'Collectively, the data do not support the presence of 4R-WGD in Japanese' -> Japanese eelLine 470: 'The duplicated genes might have strengthened immune-related responses against different pathogens, contributing to the decline of eel populations (Danne et al. 2022; 472 Bandin et al. 2014; Kennedy 2007). Presumably, physiological fitness for adaptation might have been weakened by changes in the ecological environment, causing these evolutionary novelties (Belyayev 2014). Notably, the positive selection of immune-related genes indicates the adaptive advantages of the additional TD and PD duplication.'I commented on this before - it is not entirely clear how very recent (decline of populations) and evolutionary changes in deeper time (adaptive advantages of duplication) are causally intertwined with 'changes in the ecological environment'. This suggests the duplications and evolutionary novelties are the result of recent ecological changes associated with population decline, which is probably not what you meant?

**Level of Interest**

Please indicate how interesting you found the manuscript: Choose an item.

**Quality of Written English**

Please indicate the quality of language in the manuscript: Choose an item.

**Declaration of Competing Interests**

Please complete a declaration of competing interests, considering the following questions:

- Have you in the past five years received reimbursements, fees, funding, or salary from an organisation that may in any way gain or lose financially from the publication of this manuscript, either now or in the future?

- Do you hold any stocks or shares in an organisation that may in any way gain or lose financially from the publication of this manuscript, either now or in the future?
- Do you hold or are you currently applying for any patents relating to the content of the manuscript?
- Have you received reimbursements, fees, funding, or salary from an organization that holds or has applied for patents relating to the content of the manuscript?
- Do you have any other financial competing interests?
- Do you have any non-financial competing interests in relation to this paper?

If you can answer no to all of the above, write 'I declare that I have no competing interests' below. If your reply is yes to any, please give details below.

I have no competing interests.

I agree to the open peer review policy of the journal. I understand that my name will be included on my report to the authors and, if the manuscript is accepted for publication, my named report including any attachments I upload will be posted on the website along with the authors' responses. I agree for my report to be made available under an Open Access Creative Commons CC-BY license (http://creativecommons.org/licenses/by/4.0/). I understand that any comments which I do not wish to be included in my named report can be included as confidential comments to the editors, which will not be published.

Choose an item.

To further support our reviewers, we have joined with Publons, where you can gain additional credit to further highlight your hard work (see: https://publons.com/journal/530/gigascience). On publication of this paper, your review will be automatically added to Publons, you can then choose whether or not to claim your Publons credit. I understand this statement.

Yes Choose an item.
