# Supplementary material for: A Chromosome-level assembly of the Japanese eel genome, insights into gene duplication and chromosomal reorganization
Source: Gigascience. 2022 Dec 8;11:giac120. doi: 10.1093/gigascience/giac120 (PMC9730501; doi:10.1093/gigascience/giac120)
Supplement: giac120_Reviewer_2_Report_Original_Submission — Zhong Li -- 8/22/2022 Reviewed [file giac120_reviewer_2_report_original_submission.pdf]

Reviewer Report

Title: A Chromosome-level Assembly of the Japanese Eel Genome, Insights into Gene Duplication and Chromosomal Reorganization

Version: Original Submission **Date:** 8/22/2022

Reviewer name: Zhong Li

Reviewer Comments to Author:

This manuscript by WANG et al. titled "A Chromosome-level Assembly of the Japanese Eel Genome, Insights into Gene Duplication and Chromosomal Reorganization " provides a high quality genome assembly of Japanese Eel, and economically important fish. The authors have used for kinds of sequencing technologies, and assembling strategies, and provided well annotated genomes. This genome provides useful information for the genome organization and evolution and other fields of this species.Overall, the manuscript is sufficiently descriptive and easy to follow. I have three major concerns:1. The genome annotation rely on the transcriptome. No detailed information was given the the method section.2. The analyses do not include command lines or software versions and thus are not repeatable easily. A document that include these information is higly recommended included as a supplementary file.3. The genome assembly seems has not been released on NCBI database (https://www.ncbi.nlm.nih.gov/bioproject/?term=PRJNA852364). Besides, the gene models (nucleotide, protein, and GFF files) should also be made available and included in the Data Availability section when the manuscript is accepted.

Level of Interest

Please indicate how interesting you found the manuscript: Choose an item.

Quality of Written English

Please indicate the quality of language in the manuscript: Choose an item.

Declaration of Competing Interests

Please complete a declaration of competing interests, considering the following questions:

- Have you in the past five years received reimbursements, fees, funding, or salary from an organisation that may in any way gain or lose financially from the publication of this manuscript, either now or in the future?
- Do you hold any stocks or shares in an organisation that may in any way gain or lose financially from the publication of this manuscript, either now or in the future?
- Do you hold or are you currently applying for any patents relating to the content of the manuscript?

- Have you received reimbursements, fees, funding, or salary from an organization that holds or has applied for patents relating to the content of the manuscript?
- Do you have any other financial competing interests?
- Do you have any non-financial competing interests in relation to this paper?

If you can answer no to all of the above, write 'I declare that I have no competing interests' below. If your reply is yes to any, please give details below.

I declare that I have no competing interests

I agree to the open peer review policy of the journal. I understand that my name will be included on my report to the authors and, if the manuscript is accepted for publication, my named report including any attachments I upload will be posted on the website along with the authors' responses. I agree for my report to be made available under an Open Access Creative Commons CC-BY license (http://creativecommons.org/licenses/by/4.0/). I understand that any comments which I do not wish to be included in my named report can be included as confidential comments to the editors, which will not be published.

Choose an item.

To further support our reviewers, we have joined with Publons, where you can gain additional credit to further highlight your hard work (see: https://publons.com/journal/530/gigascience). On publication of this paper, your review will be automatically added to Publons, you can then choose whether or not to claim your Publons credit. I understand this statement.

Yes Choose an item.
